# Genetic ancestry and monogenic disease risk in the Scottish Traveller founder population

**DOI:** 10.1038/s41467-026-74969-y

**Published:** 2026-07-15

**Authors:** Ashwini Shanmugam, Benjamin S. Fletcher, Maria Pala, Lucija Klaric, Shona M. Kerr, Samantha Whyte Donaldson, Gannie Tzoneva, Alan R. Shuldiner, Martin B. Richards, Russell L. McLaughlin, Gianpiero L. Cavalleri, Ross P. Byrne, Edmund H. Gilbert, James F. Wilson

**Affiliations:** 1 School of Pharmacy and Biomolecular Sciences, Royal College of Surgeons in Ireland, Dublin, Ireland; 2 The SFI Research Ireland Centre for Research Training in Genomics Data Science, School of Mathematics, Statistics and Applied Mathematics, University of Galway, Galway, Ireland; 3 The FutureNeuro Research Ireland Centre for Translational Brain Science, Dublin, Ireland; 4 Centre for Global Health Research, Usher Institute, University of Edinburgh, Edinburgh Bioquarter, Scotland; 5 School of Applied Sciences, University of Huddersfield, Huddersfield, UK; 6 C/o Centre for Global Health Research, Usher Institute, University of Edinburgh, Edinburgh Bioquarter, Scotland; 7 Regeneron Genetics Center, Tarrytown, NY USA; 8 Complex Trait Genomics Laboratory, Smurfit Institute of Genetics, School of Genetics and Microbiology, Trinity College Dublin, Dublin, Ireland

**Keywords:** Population genetics, Clinical genetics

## Abstract

The Scottish Travellers are a traditionally nomadic community in Scotland that has historically been marginalised, and remained socially isolated from the settled Scottish population until recently. Little, however, is known about their genetic origins, population structure and risks of Mendelian disease. After an approach from the community to address this gap and increase representation, we analyzed array genotypes and whole-exome sequencing data from up to 125 Gypsy/Traveller individuals, alongside settled British and Irish references. We demonstrate that Scottish Travellers are genetically distinct from Irish Travellers, English Gypsies and European Roma, as well as the settled British and Irish populations. However, they do share autosomal and mitochondrial genetic ancestry with settled Scots. Two genetic subgroups are detectable: one which is more drifted and one more admixed. High levels of autozygosity are apparent, consistent with consanguinity. We detect signals of bottlenecks in autosomal and mitochondrial data. Importantly, we identified an enrichment of rare, pathogenic variants, including at least five putative founder variants associated with recessive Mendelian disorders. These findings provide insights into the genetic history of the Scottish Traveller population and highlight the opportunity and need for community-driven clinical genetics screening initiatives to decrease the scope for further health disparities.

## Abstract (web summary)

A community-driven genetic study of the Scottish Travellers reveals them to be distinct from the Romani, as well as the settled British, but having Scottish ancestry. Targeted research and healthcare will be required, due to the increased risk of certain inherited diseases.

## Introduction

The Scottish Travellers are a historically nomadic community from Scotland with a strong sense of their cultural traditions. Some identify as Highland Travellers and some as Lowland Travellers, although most use the name Scottish Travellers^1,2^. They were recognised as a distinct ethnic group in 2008 under the terms of the Race Relations Act 1976 (K MacLennan v Gypsy Traveller Education & Information Project S/13272/07f599/132^3^;). A trust gap between some in the community and the state likely leads to under-counting, but 3333 people identified as Gypsy/Traveller in Scotland in the 2022 census^4^. Whilst there is inevitably conflation with other Traveller populations (such as English Gypsies and Irish Travellers), the Roma (from Mainland Europe) were able to identify as such in 2022—and in the 2011 census ~3200 of 4202 people in Scotland declaring Gypsy/Traveller identity were born in Scotland^5^. Therefore, it is likely that the great majority are Scottish Travellers.

The Scottish Travellers preserve a strong oral culture of songs and ballads, with many famous musicians and story-tellers. In the Highlands they spoke a Gaelic-based argot or cant called Beurla Reagaird, whereas in the Lowlands, Scottish Cant was spoken, which has Scottish Gaelic, Scots and Romani influences^6^. Originally living in skin or canvas bow tents, made with willow or hazel branches, extended families of Travellers moved around Scotland following their traditional trades, including berry picking, pearl fishing, basket making, hawking, horse dealing and itinerant tin smithing^7–9^. This last occupation gave rise to the name “tinker”, considered derogatory by most, but sometimes used by the community^10^ and originating from the Gaelic tinceard, tin craftsman. Some Scottish Travellers identify themselves as Nawken or Nachin^11,12^ and many today no longer travel, but live in trailers/caravans, some on permanent sites, or in houses.

There is sparse historical documentation of the Scottish Travellers – they have been largely absent or misrepresented in the history of Scotland^13^ and often are not clearly distinguished from other nomadic peoples^14^. The first mention of travelling people in Scotland, for example, is the farandman law enacted in the 12th century which allowed faring or travelling men a degree of legal protection^15^. Another early reference is to payments to the King of Rowmais from King James IV of Scotland in the fifteenth century^1^. There are thus conflicting narratives regarding Traveller origins. Some sources suggest that the Scottish Travellers are a branch of the European Romani, others say they originated from the indigenous Scots^16^ (or even Picts^6^), for instance after the crackdown on Highland clan culture following the Battle of Culloden in 1746—tinsmiths were reputedly armourers of clansmen who some say then took to the road^1,17^.

Regardless of their origins, the Scottish Travellers have been marginalised throughout history—they remained socially isolated from the settled communities for centuries, a feature that only changed recently (https://www.gov.scot/publications/gypsy-travellers-in-scotland-an-analysis-of-scotlands-census-2022/pages/health/)^17^. Conflict between Travellers and the state led to centuries of discrimination, for example, the 1571 Act of Stringency and 1609 Act Regarding the Egyptians, both of which legalised hanging of Gypsies^18^, the 1865 Trespass (Scotland) Act^19^ which criminalised use of traditional campgrounds, among others^1^. From the 1940s to the 1980s, the persecution included forcible settlement and in some cases removal of children^20,21^. Bullying is widespread^22^ and 62% of Gypsy/Travellers reported racial insults, property damage or physical attacks in a recent large survey^23^.

There is also significant poverty among the Gypsy/Traveller population in Scotland: 51% of working age Gypsy/Travellers are in the lowest social grades (DE), versus 26% of the population as a whole and only 7% are in the highest grades (AB), compared to 19% of the population. Over half of Gypsy/Travellers ≥16 years of age have no qualifications (51% versus 3% for white British)^24^. The history of socio-economic disadvantage and discrimination is associated with poor health outcomes among Gypsy/Travellers in Scotland. For example, age-standardised rates of long-term limiting health problems or disabilities were twice that among white Scottish, being the highest of all ethnic groups, for both men and women. Gypsy/Travellers were twice as likely to report ≥3 categories of long-term health condition and had >3.5X the rate of bad or very bad general health than the white Scottish^5^. In fact, a small survey of a Scottish Traveller community by a GP in Argyll reported their life expectancy in 2001 to be only 55, last observed in Scotland as a whole in 1932^25^. Gypsy/Travellers also had the highest rate of hospitalisation and death in Scotland, after testing positive for COVID-19^26^. These injustices have led to human rights commitments from the Scottish Government to improve the representation, access to services, accommodation, and incomes of the community, while tackling racism and discrimination^27^, with extensive recommendations also by the UK Government^28^.

The genetics of the Scottish Travellers have not been previously characterised. The community has been hesitant to participate in scientific studies, given the historical persecution and discrimination they have faced^26,29,30^. Genetic studies could, however, be beneficial to the population. Given the practice of endogamy and the small population size^1,2^, and like the Irish Travellers^31^, they may have a higher incidence of specific hereditary disorders, particularly recessive conditions, due to the effects of genetic drift (https://www.gov.scot/publications/gypsy-travellers-in-scotland-an-analysis-of-scotlands-census-2022/pages/health/)^32^. Genetic research has the potential therefore to elucidate both the population history and risk of certain genetic disorders, enabling population-specific healthcare choices.

We agreed the aims of the study through discussion with the Traveller Genes Participant & Public Involvement (PPI) committee and developed the study documents together, including the participant information sheet and protocol. We aimed to (a) infer whether the Scottish Traveller community is genetically distinct; (b) clarify the genetic origins of the Scottish Travellers and the extent of genetic relatedness to the settled Scottish populations and other Gypsy/Traveller communities; (c) test whether there is any genetic structure within the Scottish Traveller community; and (d) identify known pathogenic variants causing monogenic diseases, including any founder effects, to better understand community-specific health risks.

The study thus addresses a significant gap in genetic and health data for this community, shaped by unique social and cultural contexts, and could inform healthcare strategies and policy, while decreasing the scope for further health disparities^33^. We followed American Society of Human Genetics and UK Standards for Public Involvement (https://sites.google.com/nihr.ac.uk/pi-standards/) guidance through engagement and partnering with this historically marginalised community, aiming to increase their representation in genetic research and contribute to addressing health inequities, so that Scottish Travellers are able to realise the benefits of genetic and genomic research^34^.

## Results

### Participants and data

After an approach from the community and in consultation with the Participant & Public Involvement (PPI) committee, the Traveller Genes project recruited 148 participants with at least two grandparents with any kind of self-reported Traveller background (principally Scottish Traveller) between 2021 and 22 (see “Methods”) (Table 1 and Supplementary Data 1). In total, 125 of the 148 remained for analysis after sample QC. The mean age was 56 years and 65% were female. All four grandparents of 45 participants were of Gypsy/Traveller background, and 33 of this 45 had all Scottish Traveller grandparents (including Highland Traveller, Lowland Traveller or Scottish Traveller). Participants with all 4 Scottish Traveller grandparents were labelled SctTrv, those with all Irish Traveller grandparents were labelled IrlTrv, those with English Gypsies as GypTrv, and finally those with Traveller and also settled (non-Traveller) population ancestry as Mixed. We note a report by the UK House of Commons Women and Equalities Select Committee, which highlighted that “while some find the term ‘Gypsy’ to be offensive, many…were proud to associate themselves with this term”^28^. Our volunteers also self-identified using the ethnonym ‘English Gypsies’ and therefore we did not consider it pejorative—unlike its use associated with the Continental European Romani^35^. All Traveller Genes participants with DNA passing QC were exome sequenced, and 48 unrelated participants representing the different ancestries with four Traveller grandparents (*n* = 30), three Traveller grandparents (*n* = 13) or two Traveller grandparents (*n* = 5) had additional genome-wide SNP array genotypes generated, for joint analysis with reference populations.

**Table 1:** Ancestry of Traveller genes participants with at least 2 Gypsy/Traveller grandparents

Traveller Ancestry	4 grandparents	3 grandparents	2 grandparents	Total
Scottish Travellers	33	11	54	98
Irish Travellers	5	0	5	10
English Gypsies	1	5	8	14
Other Traveller ancestry	0	0	3	3
Total	39	16	70	125

Scottish Traveller includes Highland Traveller, Lowland Traveller and Scottish Traveller.

Only two participants with samples passing QC declared partial Lowland Traveller ancestry, which, assuming similar levels of engagement with the study, suggests either decreased numbers identifying as such, or widespread assimilation into the settled population. Among 78 Travellers with full grandparental data in our sample and who were born in Scotland, there is an apparent increase in admixture with settled Scottish individuals in later decades: only 19% of participants born after 1970 have four Traveller grandparents, versus 55% for those born before 1970 (one-tailed p-value < 0.0001, χ^2^). In analysis of parental and grandparental data, focussing on 49 marriages taking place in Scotland between Scottish Travellers and settled people (known as country people or scaldies to Travellers), there was no strong directionality to this assimilation, with 23 marriages between a Traveller groom and a settled bride, against 26 with a Traveller bride and settled groom.

### Surnames and genealogies

An analysis of 260 distinct grandparents (occasionally parents) of our participants, declared to be Scottish, Highland, Lowland or Border Travellers, and born in Scotland 1851–1950, revealed a total of 69 surnames, 38 of which only appeared once (not shown for privacy reasons). At the other end of the scale, seven surnames accounted for a total of 58% of Scottish Traveller grandparents: Stewart, McPhee, Williamson, Townsley, Reid, MacDonald and Whyte. There was little differentiation between the two most common declared groups (Highland and Scottish), but some surnames were more frequent in one group and rarer in the other, for instance Williamson among declared Highland Travellers, and Reid and Townsley among declared Scottish Travellers. The latter group also included individuals with surnames that were also found among declared Lowland Travellers.

Traveller grandparents were born in almost all the historic counties of Scotland, from Shetland to Roxburghshire, but certain surnames showed particular geographic associations, e.g., 47% of Stewart grandparents were from Aberdeenshire, whereas 61% of McPhee grandparents were from Caithness. The highest counts of Traveller grandparents came from Aberdeenshire, Perthshire, Caithness, Ross-shire, the Western Isles and Argyll, and we also recruited from a diaspora in the USA/Canada. There was no clear geographic boundary between declared Highland Traveller and Scottish Traveller grandparents, but 95% of Highland Traveller grandparents were born in counties in the Highlands of Scotland. However, among declared Scottish Traveller grandparents, 22% were from Highland counties, 31% from Lowland counties and 47% from counties at the boundary of the two, e.g., Perthshire and Aberdeenshire. There was also some fluidity across categories, for example, where children reporting the identity of the same parent or grandparent declare different identities, Scottish Traveller or Highland Traveller. Given this variability in description, we did not separate declared Highland Travellers and Scottish Travellers in the genetic analyses.

Four pedigrees of now-deceased Scottish Travellers from different families were traced, revealing an endogamous community with high levels of isonymy (surname sharing) and cousin marriage. As an example, in an individual from Orkney, 6/8 great-grandparents and 8/16 great-great-grandparents had the surname Newlands, while 3/16 each are Williamsons and McPhees. The proband’s parents share the unusual relationship of quadruple first cousins once removed and double second cousins once removed (F_ped_ = 0.156). The consanguinity arises from pedigree loops induced by a sibset exchange in the generation born in the 1860s–70s: five siblings from one family married five siblings from another sibship, all of whom were first cousins to one another. We note that sibset exchange was also practiced extensively among the Irish Travellers^36^. Similar patterns are seen in Williamson and Whyte pedigrees, with Caithness and Perthshire origins, respectively. In the former case, 6/8 great grandparents are Williamsons, as are 6/16 great great grandparents, while 4/16 are Stewarts. In the latter case, 5/8 great grandparents are Whytes, as are 7/16 great great grandparents, while 5/16 were Stewarts, with 2/16 untraced. Finally, the pedigree of a Stewart individual revealed that while only 1/16 great great grandparents carried the Stewart surname, 3/16 were McPhees and 3/16 were Whytes (with 1/16 untraced).

### Genetic Ancestry of the Scottish Travellers

**Fig. 1:**
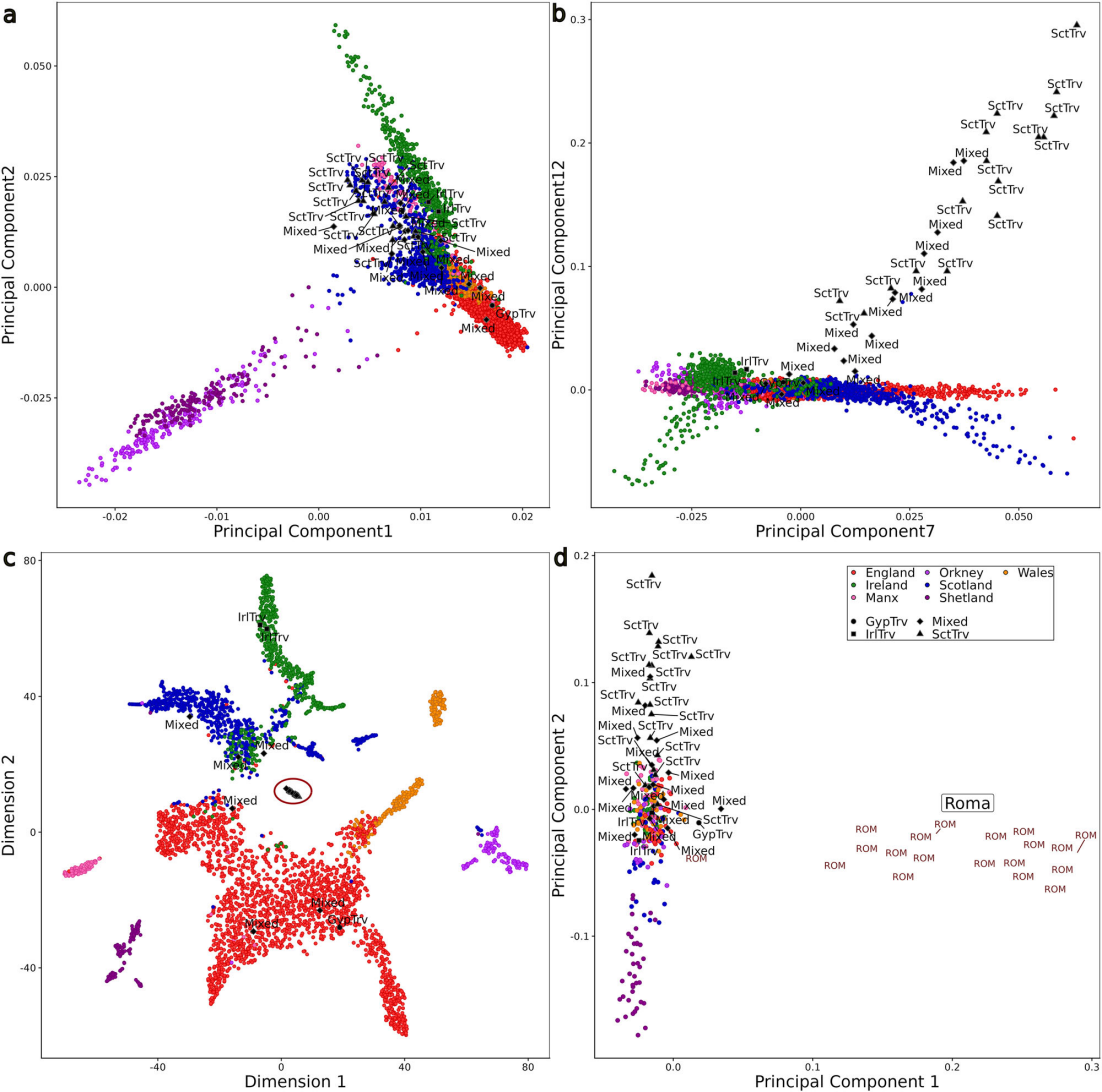
Scottish Traveller population structure. **a** The first and second principal components of a pbwt-paint chunkcounts matrix recording haplotype sharing between Traveller (black points) and settled British or Irish reference samples; **b** two higher principal components of the same PCA as (**a**), showing the separation of Scottish Travellers; **c** t-SNE dimension reduction of the top 15 principal components from the same PCA as (**a**) and (**b**). The cluster of Scottish Travellers is highlighted in a red circle; **d** the first and second principal components of an unlinked PLINK PCA combining Traveller and settled references with Iberian Roma references.

Due to conflicting narratives around the origins of the Scottish Travellers, we first sought to confirm whether they were genetically similar to the settled Scots and other Traveller ancestries. To investigate population structure and genetic relatedness, we combined this dataset with settled British and Irish references (see “Methods”). Principal components analysis (PCA) of the co-ancestry matrix generated using pbwt-paint^37^, showed that the Scottish Travellers are genetically most similar to the reference Scottish samples, while the Irish Traveller samples cluster with the reference Irish samples, and the English Gypsies with the English references (Fig. 1a). Thus, the Scottish Travellers appear genetically distinct from the Irish Travellers and English Gypsies, and share some affinity with settled Scots (Fig. 1a). Further, in higher PCs (Fig. 1b) and using a t-SNE analysis of the top 15 pbwt-paint PCs (Fig. 1c), we observed that the Scottish Travellers also form their own very distinct cluster, indicating that they are genetically differentiated from the settled Scots, despite broad similarity. Repeating these analyses without the outlier Orcadian, Manx and Shetlander samples reveals very similar patterns, but with Scottish Travellers now separating on PC8 (Supplementary Figs. 5, 6). Complementing this, we investigated the evidence of genetic links between the Scottish Travellers and the European Roma by combining the Scottish Traveller and British and Irish reference genotypes with Iberian Roma genotypes (see “Methods”). In PCA of these genotypes, there is a cline on PC1 (Fig. 1d) that separates the Iberian Roma from the British, Irish and Traveller genotypes, while PC2 separates Scottish Travellers from Orcadians and Shetlanders. Two samples show slightly more genetic affinity with the Roma, being nearer the main Roma cluster on PC1 than any of the other samples, with both individuals reporting at least three English Gypsy grandparents and no Scottish Traveller grandparents. There is no evidence of a genetic relationship between the Scottish (or Irish) Travellers and the European Roma.

### Genetic structure within the Scottish Travellers

**Fig. 2:**
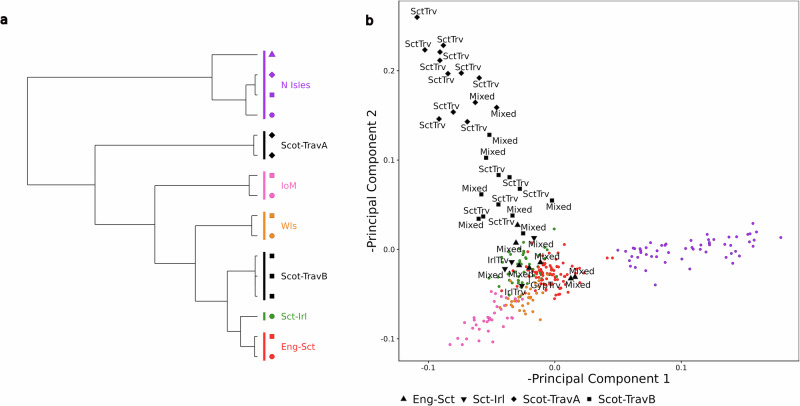
Fine-scale genetic structure within the Scottish Travellers. **a** The fineSTRUCTURE dendrogram of the Traveller dataset and British and Irish references. Merged final clusters are shown with the same colour. N Isles, Northern Isles of Scotland (Orkney and Shetland); IOM, Isle of Man; Wls, Wales; *Sct-Irl* and *Eng-Sct* are clusters observed across Scotland and Ireland, and England and Scotland, respectively; *Scot-TravA* and *Scot-TravB* are clusters observed among the Scottish Travellers. **b** PCA of the chunkcounts matrix from CHROMOPAINTER. The reference samples in the PCA have colours corresponding to the higher order cluster (see colours in **a**) and are plotted as circles. The shape of Traveller samples (coloured in black) indicates the fineSTUCTURE cluster they are grouped into. Labels on the Traveller samples are based on their self-reported Traveller ancestry. Supplementary Data 10 presents counts of samples by population in each fineSTRUCTURE cluster.

We next investigated signals of population structure within the Scottish Traveller community itself using CHROMOPAINTER and fineSTRUCTURE^38^. We randomly subsampled the reference datasets to create a balanced representation of Scotland, England, Wales, the Northern Isles of Scotland, the Isle of Man, and Ireland (see Methods). PCA of the co-ancestry matrix from CHROMOPAINTER shows that PC2 separates Scottish Travellers from the settled populations (Fig. 2b). We detected two Scottish Traveller genetic clusters using fineSTRUCTURE (Fig. 2a), which we labelled *Scot-TravA* and *Scot-TravB* (representations of the underlying IBD network are plotted in Supplementary Fig. 7). From principal components one and two (Fig. 2b), *Scot-TravA* has drifted farther from the settled populations than *Scot-TravB*. *Scot-TravB* falls on a cline on PC2 between *Scot-TravA* and the settled Scots, consistent with the placement on the fineSTRUCTURE dendrogram (Fig. 2a), suggesting that *Scot-TravB* is more similar to the settled Scottish, Irish, and English genetic groups than *Scot-TravA*, which appears to be more genetically distinct from the settled populations (Fig. 2a).

Interestingly, Traveller participants who report non-Scottish Traveller ancestry (with the labels IrlTrv, GypTrv or Mixed) are clustered outside of *Scot-TravA* or *Scot-TravB*, clustering with either the *Eng-Sct* or *Sct-Irl* fineSTRUCTURE clusters, which account for the majority of settled British people (Fig. 2b).

We extended this analysis by modelling proportions of haplotypic similarity between the Scottish Traveller clusters and the settled populations using an adaptation of the Non-negative Least Squares (NNLS) method^39^, which instead used proportions of identical-by-descent (IBD) segment sharing as input. Complementing this NNLS method, we also estimated F_ST_ between genetic clusters (Supplementary Data 2). For the NNLS approach, we first identified fine-scale clusters in the large reference dataset using the Leiden network community detection algorithm (Supplementary Fig. 2, Supplementary Data 3)^40,41^, using the amount of IBD segment sharing as input (see Methods). This detected two tiers of clusters, larger groups which correspond to previous observations of genetic structure in Britain and Ireland^39,42–44^, and finer-scale communities within these (Supplementary Fig. 2). Using these reference clusters as donor populations allowed us to explore regional genetic affinities between the Scottish Traveller fineSTRUCTURE clusters and genetic regions of Britain and Ireland, with a focus on Scotland (Supplementary Data 4).

**Fig. 3:**
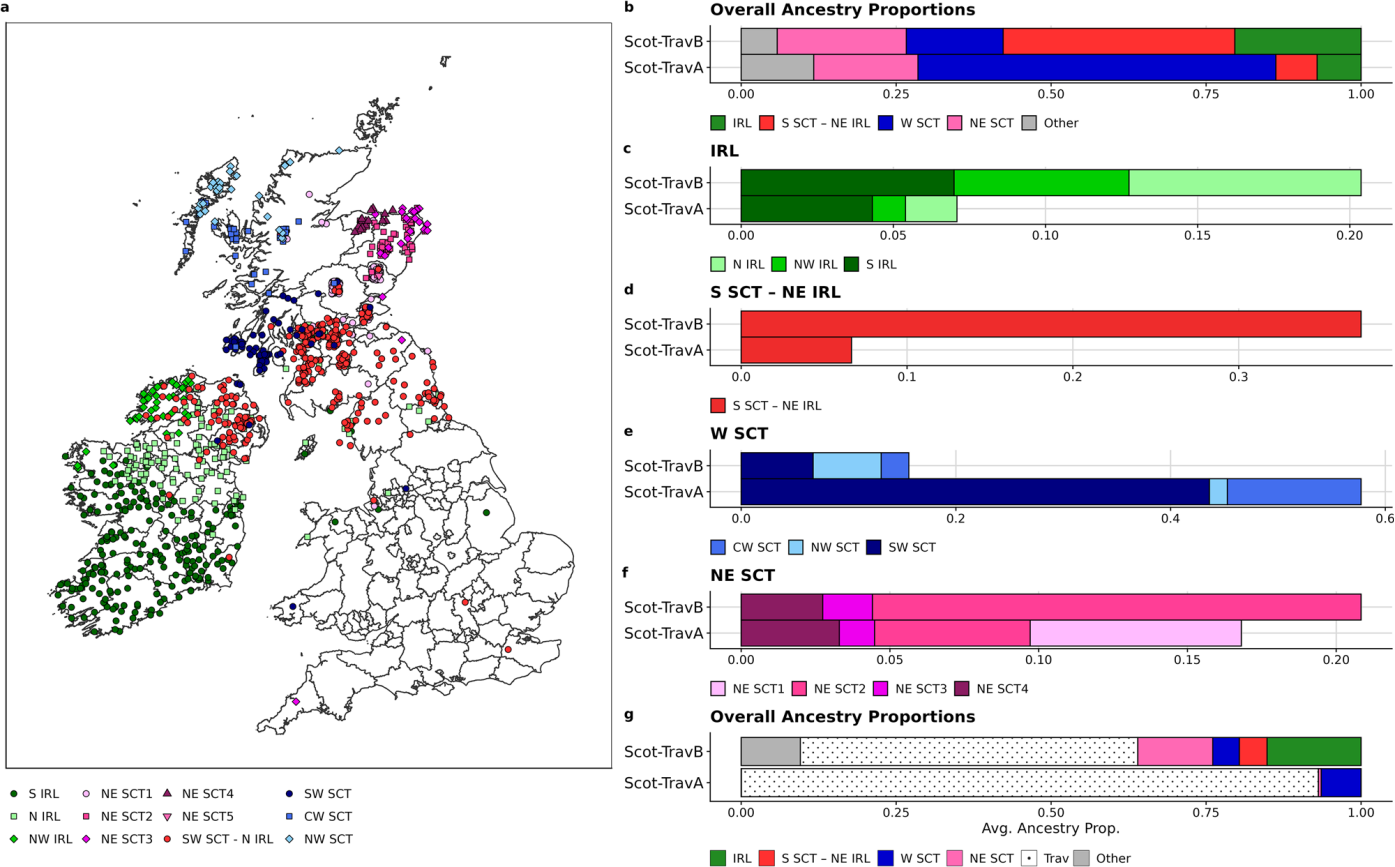
Haplotype similarity with reference British, Irish, and Scottish Traveller populations. **a** Each point on the map is the geographic origin of a participant from the reference Irish and Scottish datasets. The shape and colour of the point indicate the third-level Leiden genetic community to which they belong. The administrative boundaries for the UK and Ireland were downloaded as shapefiles from the Database of Global Administrative Areas (GADM, gadm.org, 2021). The stacked bar plots indicate the proportions of haplotypes contributed to *Scot-TravA* or *Scot-TravB* from (**b**–**f**) reference genetic communities identified using the Leiden community detection algorithm and (**g)** the other Scottish Traveller cluster (i.e. *Scot-TravA* to *Scot-TravB* and vice versa); **b** overall contributions from second-level genetic communities, **c** Ireland, **d** south Scotland, north England and north-east Ireland **e** north and west Scotland, **f** north-east Scotland and **g** overall contributions from reference communities and Scottish Traveller clusters. The colour of the bar indicates the genetic community and the maps show the geographic origin of samples in these genetic communities, with the colour of bars matching the colour of points on the map. The Scottish Traveller clusters are represented by stippling within the bar plots. Only individuals with all four grandparents declared to be Travellers were included in this analysis.

We observed that *Scot-TravA* is most genetically similar to reference genetic clusters with a predominantly northern and western Scotland membership (a total of 73% from *SW SCT*, *NE SCT*, and *CW SCT* clusters; Fig. 3b). *Scot-TravB*, in contrast, is more genetically similar to clusters with southwest Scotland and Northern Ireland membership (a total of 51.7% from *SW SCT—NE IRL*). We observed minimal sharing with England (11.3% in *Scot-TravA* and 0% in *Scot-TravB*), Wales (0% and 0.9%), and with Ireland (10.3% and 15.3%). In analysis allowing *Scot-TravA* and *Scot-TravB* to contribute to one another (Fig. 3e), *Scot-TravA* could be modelled as 93% *Scot-TravB*, while the latter only derived 54% from *Scot-TravA* (Fig. 3e), potentially suggesting *Scot-TravA* has experienced further genetic drift, after originating from a *Scot-TravB*-like ancestral Traveller group. We further tested this by computing F_ST_ between the Scottish Traveller clusters and the British and Irish reference communities. *Scot-TravA* is more genetically distant from the settled populations (mean F_ST_ of 0.006 ( ± 0.0012)), while *Scot-TravB* is closer to them (mean F_ST_ of 0.002 ( ± 0.0003)).

### Demographic history

**Fig. 4:**
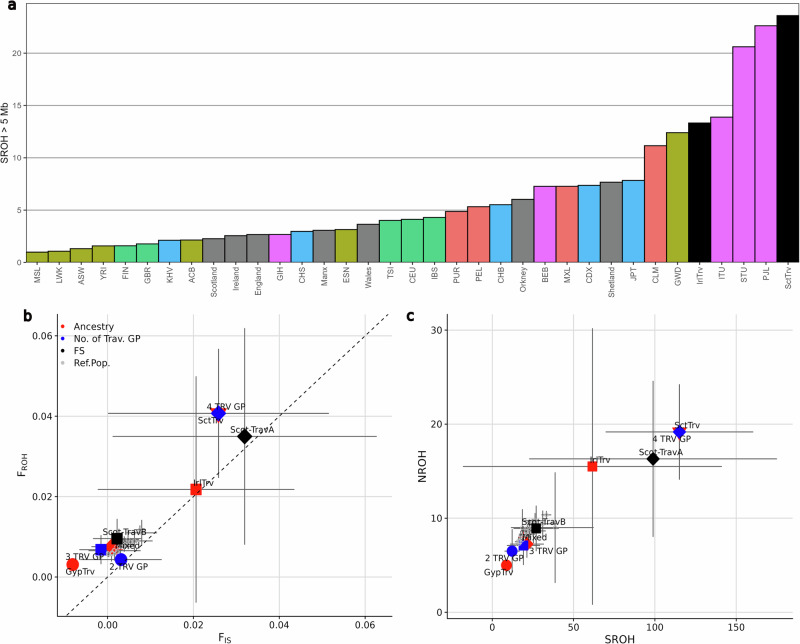
Genomic homozygosity in the Scottish Travellers. **a** We first compare the sum of the runs homozygosity over 5 Mb (S_ROH_) of Scottish Travellers, Irish Travellers, settled Scottish and Irish and reference samples from the 1000 Genomes Project. British and Irish references are coloured grey, Traveller samples are in black, and 1000 Genomes Project samples are coloured olive green for African-heritage, pink for South Asian, blue for East Asian, green for European and red for admixed American. **b**, **c** To understand the patterns of homozygosity in the Traveller community better, we divided individuals in the Traveller dataset three different ways, indicated by the colour of points and then labels for subdivisions in that specific breakdown of the dataset. Blue points are Traveller participants split by the number of self-reported Scottish Traveller grandparents (2, 3 or 4 TRV GP). The red points split the samples by the type of self-reported Traveller ancestry (SctTrv, Scottish Traveller; IrlTrv, Irish Traveller; GypTrv, English Gypsy Traveller). The black points are the Scottish Traveller genetic clusters inferred using fineSTRUCTURE (FS): Scot-TravA and Scot-TravB. The values were averaged for each category and plotted. The settled Mainland reference samples are in grey. **b** Mean F_ROH_ and F_IS_ for the Travellers and the reference populations. Mean F_ROH_ is a measure of inbreeding relative to an unknown generation from tens of generations in the past. F_IS_ measures inbreeding within the current generation. An F_IS_ value of 0 indicates random mating, F_IS_ > 0 reflects consanguinity, and F_IS_ < 0 suggests inbreeding avoidance. **c** Average sum (SROH) and number of ROH (NROH) segments (minimum length of 1500 kb) observed within each cluster mentioned earlier. The numbers in each subgroup are given in Supplementary Data 5. Error bars for (**b**, **c**) include 95% confidence intervals around the means. Abbreviations used in panel (**a**) are given in Supplementary Data 5, along with the numbers of samples per population.

Having detected population structure within the community, we investigated patterns of genomic homozygosity, to contextualise further the observed differences in ancestral affinities between the *Scot-TravA* and *Scot-TravB* clusters, and settled Scots. We wanted to assess if the differences are solely due to population structure, or alternatively elevated isolation in *Scot-TravA* compared to *Scot-TravB*. We characterised signals of isolation in the Scottish Travellers by quantifying Runs of Homozygosity (ROH) (long homozygous segments of the genome which indicate kinship between parents) and the F_ROH_ and F_IS_ statistics^45^ (Fig. 4).

Comparing the Scottish Travellers to 34 global and local populations (Fig. 4a) revealed substantial burdens of long homozygous tracts (mean sum of ROH over 5 Mb: 23.8 Mb), similar to those seen in Punjabis from Lahore, Pakistan and Sri Lankan Tamils. For ROH between 1.5 Mb and 5 Mb, the Scottish Travellers fall within the range seen for other British populations (Supplementary Fig. 4). We split the Traveller dataset in three ways: self-reported Traveller ancestry (red points), the number of Traveller grandparents (blue points), and fineSTRUCTURE cluster membership (black points). Scottish Travellers showed signals of a small effective population size consistent with genetic isolation – the average sum of ROH_≥5Mbp_ in full Scottish Travellers was even higher, at 84.9 Mb, over an average of 6.7 segments, with an F_IS_ of 0.0258, compared to the average sum of ROH_≥5Mbp_ of 2.8 Mb in British and Irish mainland reference populations over an average of 0.3 segments and an F_IS_ of 0.0016 (Supplementary Data 5). The rapid reduction in the burden of ROH in participants with 3 or 2 Scottish Traveller grandparents (average sum of ROH_≥5Mbp_ was 3.4 Mb long, from an average of 0.3 ROH), further confirms this signal (Fig. 4b, c). Within the full Scottish Travellers, we observed a trend of higher ROH in the *Scot-TravA* group (average SROH_≥5Mbp_ = 83.3 Mb) in comparison to *Scot-TravB* (average SROH_≥5Mbp_ = 23.8 Mb, Student’s t = 0.94, *p* = 0.18) that suggests greater isolation in *Scot-TravA*, consistent with our observations in PCA (Fig. 2b). The ROH burden observed in *Scot-TravB* was still ~8-fold greater compared to the reference populations. *Scot-TravA* also appears to be more consanguineous compared to *Scot-TravB*, with a high F_IS_ value (0.036), similar to the F_ROH_ (0.037), thus following the linear relationship expected under consanguinity^45^. To focus more clearly on ROH arising from recent pedigree loops, we excluded runs below 10 Mb in size. Full Scottish Travellers have on average 62.5 Mb of their genome in ROH above this size, compared to only 1.7 Mb for Mainland reference populations (35 times higher sum of very long ROH; not shown), consistent with recent consanguinity.

**Fig. 5:**
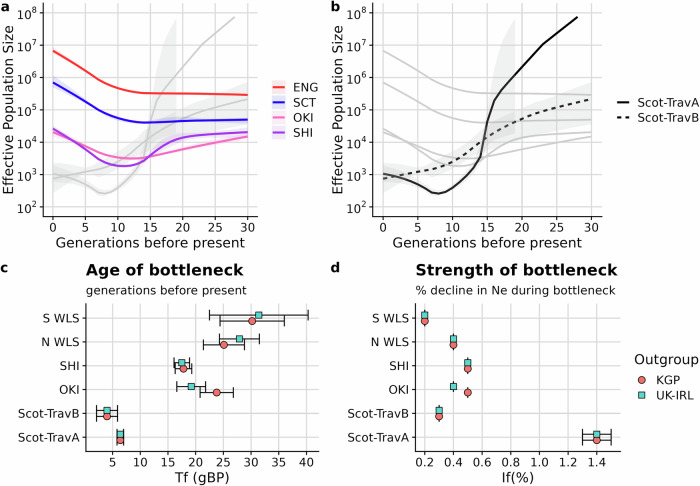
Signals of a bottleneck in Scottish Travellers. **a**, **b** The effective population size trajectories show changes in N_*e*_ over generations from the most recent (Gen. 0) to 30 generations ago, including bottlenecks in the populations. We show (**a**) representative reference English, Scottish, Orcadian and Shetlandic populations, and **b** the Scottish Traveller genetic communities. Shaded regions around each line represent the 95% upper and lower confidence intervals around the means. The estimates of age (**c**) and intensity (**d**) of bottlenecks in representative populations and Scottish Traveller genetic communities are represented in the plots. The two colours indicate the outgroup used for the analysis—1000 Genomes project (KGP) Non-Finnish European references in red and a combination of the 1000 Genomes project and British-Irish references in blue (UK-IRL). S WLS, South Wales (*n* = 160); N WLS, North Wales (*n* = 115); SHI, Shetland Islands (*n* = 155); OKI, Orkney Islands (*n* = 160); Scot-TravB, Scottish Traveller group B (*n* = 10); Scot-TravA, Scottish Traveller group A (*n* = 13). Tf is the age of the bottleneck in generations before present (gBP), and If is the Intensity of the bottleneck, assessed as the duration / 2N_*e*_. Error bars for (**c**, **d**) include 95% confidence intervals around the means.

Lastly, we further investigated these signals of isolation in the Scottish Travellers by estimating changes in effective population sizes (N_*e*_) over time^46^ (Supplementary Data 6). Using IBDNe^46^, we observed a recent population bottleneck with the estimated N_*e*_ of *Scot-TravA* dropping from ~18,000 17 generations ago to 278 individuals 9 generations ago (a 98% decrease in N_*e*_), whereas in *Scot-TravB* the N_*e*_ reduces from ~17,800 14 generations ago to 887 seven generations ago (a 95% decrease in N_*e*_) (Fig. 5b). However, this signal has potentially been diluted by recent admixture with settled Scots (Supplementary Fig. 1). This signal of a bottleneck observed in the Scottish Travellers is stronger and more recent in comparison to known population isolates such as Orkney and Shetland, in agreement with our observations regarding homozygosity (Fig. 4). Using an independent approach, implemented in ASCEND^47^, we generated alternative estimates for the age and intensity of bottlenecks in *Scot-TravA* (T_*f*_ = 6 [95% CI: 5, 8], I_*f*_ = 1.4% [95% CI: 1.2, 1.6]), but *Scot-TravB* has a poor model fit (Fig. 5c, d, Supplementary Fig. 3).

### Mitochondrial DNA lineages

Complete mtDNA sequences were available for 91 individuals declaring their mother’s mother to be a Traveller and who were not first- or second-degree relatives. We identified 48 mitochondrial haplogroups among the Traveller groups (Supplementary Data 7, Supplementary Data 11). None of the mitochondrial lineages typically associated with the South Asian origin of the Romani people (e.g., M5a1, M18, M25, and M35)^48,49^ are present among the Gypsy/Traveller samples. Instead, 100% of the haplogroups identified are of Western European origin. Moreover, 81% of all Scottish Traveller samples have direct or indirect matches among the Traveller groups and with the local settled population, with shared haplogroups and haplotypes in particular with published Scottish mitogenomes, but also with individuals across the British Isles, and more widely in Europe (Supplementary Data 12). The small numbers of Irish Travellers and English Gypsies carried a variety of European mitochondrial haplogroups, including three English Gypsies with the H7a1a haplogroup, a potential founder effect, and one with U3b1c, another known European Roma lineage^49–51^ (Supplementary Data 13).

A major founder effect was noted among the 67 declared Scottish, Highland or Lowland Traveller mtDNAs for the T2f1a1 lineage, which occurred 16 times in the dataset, accounting for 24% of the matrilines. The lineage was observed across both *Scot-TravA* and *Scot-TravB* fineSTRUCTURE subgroups of the Scottish/Highland Travellers and in one participant with a Lowland Traveller grandmother. T2f1a1 was particularly prevalent among Scottish/Highland Travellers with Central or Northern Scottish grandmothers, but was observed in the South, West and Northeast regions as well. Further potential founder effects were noted for J1c2e, H16, K1c1b, U4b1b1, V2, J1b1a1a and U1a1a3, each accounting for 4–7% of the sample. We note that both H16 and K1c1b were also found across both *Scot-TravA* and *Scot-TravB*, whereas the others were observed in one or other group in these small samples. These eight lineages sum to 64% of the Scottish Traveller mitochondrial pool, with the remainder being made up mostly of singletons (Supplementary Data 7).

Further insights into the origins of the population come from the phylogeography of the mtDNA haplotypes. A local Scottish origin is suggested by the T2f1a1 major founder, as well as the K1c1b, J1b1a1a and U4b1b1 founders, with identical or near-identical matches to Scottish samples, branching among other northern European samples (Supplementary Data 12). The Scottish Traveller-specific branch of T2f1a1, defined by m.14319 T > C, appears specific to Scotland, with a single settled match originating in Caithness. Similar patterns are observed for numerous rarer haplogroups (e.g., U5a1b1a, I2, H1bb, H31a, U5b2b3b), with variation rooting in the British Isles and more widely Northern Europe (Supplementary Data 12). A different pattern is observed for the U1a1a3 and V2 founders (Supplementary Data 13), with the former rooting with a Spanish lineage, among Eastern European and Near Eastern (Assyrian and Mazanderani) haplotypes, and the latter branching from a clearly Mediterranean clade (with Italian, Southern French, Sardinian and Canarian members). A Mediterranean distribution is also seen for the H1b1g Scottish Traveller mtDNA, branching with Italian haplotypes (Supplementary Data 13). Further details of Traveller mitochondrial DNA lineages are given in the Supplementary Information. Notably, the Southern European connections, show-cased by mitolineages U1a1a3, V2, and H1b1g appear unique to the Scottish Travellers, with no matches among mitogenomes from the British Isles.

The evidence from autosomal and mitochondrial genetics for both isolation and a historical bottleneck suggest the potential for an enrichment of otherwise rare functional variants in the Scottish Traveller community, which we now explore.

### Rare clinically significant variants in Scottish Travellers

Exome sequences from 123 participants passed sequencing and genotype quality control thresholds (see Methods). From these sequences, we ran the variants which passed QC through a ClinVar-based pipeline^52^ to identify rare clinically significant variants and better understand community-specific health risks. To date, there is no scientific literature on the prevalence of Mendelian disorders among the Scottish Travellers. All of the variants identified through the analysis pipeline in the Scottish Traveller samples had either 3-star (reviewed by an expert panel) or 2-star (criteria provided, multiple submitters, no conflicts) evidence status in ClinVar.

### Summary of clinically significant variants

We identified 166 pathogenic/likely pathogenic (P/LP) variants (Supplementary Data 9) which were 2 star or higher: 119 singletons, 25 doubletons, and 22 variants occurring in more than two samples. These 22 variants are detailed in Table 2, all were found in a heterozygous state and the great majority act recessively. Upon closer inspection, approximately half of these variants are known to cause metabolic disorders, or dysplasia/hyperplasia, when two copies are present in an individual.

**Table 2:** List of clinically significant variants identified in > 2 Scottish Travellers ordered by uplift in frequency

Disease	Gene	Gene list	HGVSc	HGVSp	Pathogenicity	Inheritance	Uplift in ST
Ventriculomegaly with cystic kidney disease (MIM#219730); focal segmental glomerulosclerosis 9 (MIM#616220)	*CRB2*	Genome	c.3089_3104dup	p.Gly1036AlafsTer43	P/LP	AR	2500
Desbuquois dysplasia (MIM#251450)	*CANT1*	Genome	c.902_906dup	p.Ser303AlafsTer21	P/LP	AR	2371
Lymphatic malformation 6 (MIM#616843); Dehydrated hereditary stomatocytosis 1 (MIM#194380)	*PIEZO1*	Irish Traveller	c.3331 C > T	p.Gln1111Ter	P/LP	AR;AD	2075
Hyperoxaluria, primary, type III (MIM#613616)	*HOGA1*	Genome	c.569 C > T	p.Pro190Leu	P/LP	AR	1389
Myasthenic syndrome, congenital, 10 (MIM#254300)	*DOK7*	Genome	c.1124_1127dup	p.Ala378SerfsTer30	P	AR	28
von Willebrand disease, types 2 A, 2B, 2 M, and 2 N (MIM#613554)	*VWF*	Genome	c.2561 G > A	p.Arg854Gln	P/LP	AR	12
Argininosuccinic aciduria (MIM#207900)	*ASL*	Genome	c.35 G > A	p.Arg12Gln	P/LP	AR	11
Autosomal recessive deafness 8 (MIM#601072)	*TMPRSS3*	Genome	c.413 C > A	p.Ala138Glu	LP	AR	10
Combined malonic and methylmalonic aciduria, (MIM#614265)	*ACSF3*	Genome	c.1672 C > T	p.Arg558Trp	P/LP	AR	3
Fructose intolerance (MIM#229600)	*ALDOB*	Genome	c.448 G > C	p.Ala150Pro	P/LP	AR	3
Albinism, oculocutaneous (MIM#203200)	*OCA2*	Genome	c.1327 G > A	p.Val443Ile	P/LP	AR	3
Icthyosis vulgaris (MIM#146700); Atopic dermatitis (MIM#605803)	*FLG*	Genome	c.2282_2285del	p.Ser761CysfsTer36	LP	AR, AD	3
Adrenal hyperplasia, congenital; hyperandrogenism, non-classic; due to 21-hydroxylase deficiency (MIM#201910)	*CYP21A2*	Irish Traveller	c.1360 C > T	p.Pro454Ser	P/LP	AR	2
Short-rib thoracic dysplasia 14 with polydactyly (MIM#616546)	*KIAA0586*	Genome	c.392del	p.Arg131LysfsTer4	P/LP	AR	2
Ichthyosis vulgaris (MIM#146700)	*FLG*	Genome	c.1501 C > T	p.Arg501Ter	P/LP	AR;AD	2
Nonsyndromic genetic hearing loss (MIM#220290)	*GJB2*	Genome	c.101 T > C	p.Met34Thr	P	AR	2
Cystic fibrosis (MIM#219700)	*CFTR*	Genome	c.1521_1523del	p.Phe508del	P	AR	2
Hereditary haemochromatosis (MIM#235200)	*HFE*	ACMG	c.845 G > A	p.Cys282Tyr	P	AR	1
Emphysema-cirrhosis, due to AAT deficiency (MIM#613490)	*SERPINA1*	Genome	c.863 A > T	p.Glu288Val	P	AR	1
Biotinidase deficiency (MIM#253260)	*BTD*	ACMG	c.1270 G > C	p.Asp424His	P/LP	AR	0.4
Emphysema-cirrhosis, due to AAT deficiency (MIM#613490)	*SERPINA1*	Genome	c.1096 G > A	p.Glu366Lys	P	AR	0.4
Hereditary pancreatitis (MIM#167800)	*PRSS1*	Genome	c.47 C > T	p.Ala16Val	LP	AD	0.2

The variants are listed with their associated diseases, HGVSc or coding sequence change, HGVSp or protein sequence change, pathogenicity according to Clinvar where LP is likely pathogenic and P is pathogenic, whether the disorder is autosomal dominant (AD) or autosomal recessive (AR) (or has reports of both modes of inheritance), and the relative increase, or uplift, of their frequency in the Scottish Travellers (ST) compared to the frequency in non-Finnish Europeans as listed in gnomAD^87^. ACMG, American College of Medical Genetics. Genome refers to all the genes in the rest of the genome, not on either the ACMG or Irish Traveller lists. Variants in *CRB*2, *CANT*1, *ALDOB*, *KIAA0586* and *CFTR* were seen in Travellers with two or three Traveller grandparents, but not yet in those with four Traveller grandparents. As described in Methods, the frequency uplift estimates took into account the number of grandparents who were Travellers.

To identify actionable variants, as defined by ACMG^53,54^, we first focused the pipeline on a list of 81 genes, in which ACMG recommends reporting secondary findings in clinical exome and genome sequencing^55^. Seven variants were found, all in heterozygotes (see below for more details). Next, we ran the pipeline on a list of 78 genes identified to cause monogenic disorders in the Irish Travellers^31^. Fifteen variants causing recessive disease were observed, all in heterozygous state, therefore not affecting the health of the volunteer. Finally, we ran the pipeline including all other coding genes.

Given the small effective population size of the Scottish travellers, we expected to find co-occurrences of drifted P/LP variants in the participants, due to independent assortment. Among the 82 participants with at least 2 grandparents of Scottish Traveller ancestry, 68 of them carried ≥2 P/LP variants. These are all heterozygous variants causing disorders that act recessively. Five participants with at least two Irish Traveller grandparents also had ≥3 P/LP variants.

### Drifted actionable variants on the ACMG gene list

The ACMG list focuses on specific genetic disorders for which established interventions can prevent or significantly reduce morbidity and mortality^55^. We identified a total of seven variants in genes on the ACMG list, of which 4 were singletons, one a doubleton and 2 variants occurred in more than two samples. The *HFE* and *BTD* variants which were detected in >2 samples are those which are commonly observed in northwest European populations—we observed no change in their frequencies when compared to non-Finnish Europeans, in accordance with the Scottish origins of the Scottish Travellers. These variants were observed in similar numbers of samples from both *Scot-TravA* and *Scot-TravB* clusters. No obvious founder effects were observed for actionable variants that act dominantly in genes such as *BRCA1*^56,57^ *BRCA2* ^58^, *LDLR* and *TTN*^52^, and indeed no carriers of such variants were observed in our sample.

### Drifted pathogenic variants in genes known to cause disease in Irish Travellers

The Irish Travellers are an ethnically Irish, traditionally nomadic community within Ireland^36^. As part of recruitment for the Traveller Genes project, several individuals with Irish Traveller ancestry were recruited to explore links with the Scottish Travellers—but also allowing a glimpse into the clinically important variants present in the Irish Traveller population. Due to the preference for consanguinity within the Irish Traveller community, there is a high prevalence of well-recognised autosomal recessive disorders. We tested if participants in our dataset, including 10 with at least two grandparents of Irish Traveller ancestry, carried pathogenic variants previously reported in members of the Irish Traveller community^31^. We detected 15 such variants across our Traveller samples: 9 singletons, 3 doubletons, and 3 variants which occurred in more than two samples (Supplementary Data 9).

Two doubletons were detected at *THOC6*, each with an uplift of over ~450-fold in the Irish Travellers, and both were absent in the Scottish Travellers. While these variants, known to cause Beaulieu-Boycott-Innes syndrome [MIM #613680], were previously observed in the Irish Traveller variant catalogue within one nuclear family^31^, they were identified in unrelated Irish Traveller samples in our dataset, indicating that they are likely found in Irish Travellers at frequencies higher than previous reports, through genetic drift. The two *THOC6* variants co-occurred in the same two Irish Traveller samples, suggesting they are present on the same haplotype. A further three variants from the Irish Traveller list were observed once each in our Irish Traveller samples, as were a further nine singletons and nine multiply observed variants that were not reported by Lynch et al.^31^.

We observed a *PIEZO1* c.3331 C > T truncating variant, which causes lymphatic malformation 6 [MIM #616843], a form of generalised lymphatic dysplasia. The variant was detected only in Scottish Traveller samples (*n* = 5), with 2075-fold uplift compared to non-Finnish Europeans, indicating a strong founder effect. Two of these five samples were inferred to be kin (parent-offspring), while the others were unrelated up to 4th degree kinship. The *PIEZO1* variant might be specific to *Scot-TravA*—two samples were classified into this cluster in the fineSTRUCTURE analysis, while none belonged to *Scot-TravB*. The variant we identified is different to the one reported in the Irish Traveller Catalogue^31^, which was private to a nuclear family. The remaining variants in genes in the Irish Traveller list were also different to those previously published, excepting the common *CFTR* p.Phe508del (ΔF508) variant, causing cystic fibrosis, and which was observed at typical northwest European frequency.

### Drifted clinically significant variants in the rest of the coding genes

We extended our search for P/LP variants to the rest of the protein-coding genes and identified 107 singletons, 22 doubletons, and 17 variants occurring in more than two samples (Supplementary Data 9). Despite the small sample size, we were able to identify strong founder effects for four recessive P/LP variants which were uplifted in Scottish Travellers at least 30-fold compared to the non-Finnish Europeans, and two additional potential founder variants.

We observed a 2500-fold uplift of the *CRB2* c.3089_3104dup frameshift variant, which causes focal segmental glomerulosclerosis 9 [MIM #616220] in homozygotes, leading to proteinuria and progressive decline in renal function. This variant is ultra-rare, i.e., was absent in gnomAD. Two of the three carriers of this variant are inferred to have 4th degree kinship, but are unrelated to the third carrier. The second most enriched variant is a *CANT1* c.902_906dup frameshift variant which, in homozygous state, causes Desbuquois dysplasia 1 [MIM # 251450], a chondrodysplasia in the multiple dislocation group, with a 2371-fold uplift. Two of the three carriers of this variant are full siblings but are unrelated to the third carrier. The third variant, a *HOGA1* c.569 C > T variant, which causes primary hyperoxaluria, type III [MIM #613616] when homozygous, was observed at a 1,389-fold uplift in the Scottish Travellers. There are two pairs of kin among the 5 carriers of the variant—a parent-offspring duo and a 4th degree kin pair. The two kin pairs are unrelated to each other and to the final carrier of the *HOGA1* variant. The final founder variant is a *DOK7* c.1124_1127dup frameshift variant that causes congenital myasthenic syndrome 10 [MIM #254300] in homozygotes, a disorder of the neuromuscular junction causing muscle weakness, with a 30-fold uplift. All carriers of this variant were inferred to be unrelated to the fourth degree, however, we inferred two carriers might be 5th degree kin. We identified two other potential founder variants. The first one was *VWF* c.2561 G > A which was estimated to be 12-fold higher in Scottish Travellers, and is known to cause Von Willebrand Disease [MIM #193400, 277480, 613554], a bleeding disorder. Another potential founder variant we detected was *ASL* c.35 G > A, which is approximately 14-fold uplifted and causes argininosuccinic aciduria, a metabolic disorder of the urea cycle. Mildly increased frequencies were seen for a number of variants including a fructose intolerance variant at *ALDOB*.

Finally, as the clinically significant variants which were enriched in the Scottish Travellers are unique to them and have not been reported in the Irish Travellers, it corroborates our findings based on genome-wide relatedness that the two communities are indeed genetically distinct.

## Discussion

After a request from a representative Scottish Traveller, we designed the Traveller Genes study protocol with input from community members and received a favourable opinion from a Research Ethics Committee. Recruitment of nearly 150 volunteers with self-declared Traveller grandparents allowed us to explore the ancestry using both genealogies and genetics. Genome-wide SNP array data were used to investigate the genetic ancestry of the population and relationships to settled Scots and other Gypsy/Traveller communities in the British Isles. We assessed the degree of genetic structure among Scottish Travellers, their demographic history and mitochondrial variation. Finally, we used whole exome sequence data to identify recessive pathogenic variants which have undergone founder effects and are in some cases thousands of times more common in this population than in reference datasets.

This is the sole genetic study of the Scottish Travellers and should therefore help to clarify the debate around their ancestral ties. Using principal components analysis, we clearly show genetic differentiation from the European Roma (and English Gypsies), refuting suggestions of shared origins. Our results establish that Scottish Travellers are also genetically distinct from Irish Travellers, who, as previously shown^59^, are closely related to the settled Irish population. This differentiation underscores the unique genetic identity of the Scottish Travellers within the broader context of Gypsy/Traveller and Roma communities.

A close relationship to the settled Scottish population is evident from multiple analyses, including clustering of Scottish Travellers with reference settled Scottish individuals in factor analysis and significant haplotype sharing between these populations. The major phylogeographic pattern in the mtDNA also confirms the ancient Scottish descent of the lineages. The Scottish Travellers therefore have a Scottish genetic ancestry, but at the same time they form a unique and cohesive cluster in high resolution analysis, such as t-SNE analysis of shared haplotypes, showing their gene pool to be distinct from all other British and Irish populations. The distinct genetic pool is also reflected in mitochondrial DNA variation, such that 24% of maternal lineages belong to one founder haplotype, and eight such lineages account for 64% of Scottish Traveller matrilines.

We also discovered genetic structure within the Scottish Traveller population. Using fineSTRUCTURE, we identified two genetic subclusters: a larger one with predominantly western and north-eastern Scottish-like ancestry (*Scot-TravA*) and another with predominantly western and north-eastern Scottish, as well as southern Scottish/north-eastern Irish, ancestries (*Scot-TravB*). Analyses allowing each of the Scottish Traveller clusters to be the donor (ancestor) of the other reveals that *Scot-TravA* can be modelled as almost entirely from *Scot-TravB* haplotypes, whereas only half the variation in *Scot-TravB* can be represented from *Scot-TravA*. One interpretation is that *Scot-TravA* represents a more strongly drifted descendant population, while *Scot-TravB* remains nearer to the ancestral gene pool. Another possibility is that *Scot-TravA* is more similar to the original population, retaining a more distinctive signature through isolation and stronger drift, while *Scot-TravB* received more gene flow.

PCA of the CHROMOPAINTER coancestry matrix suggests further complexity: *Scot-TravA* has drifted further from the settled populations, while *Scot-TravB* samples lie on a cline between *Scot-TravA* and the reference British samples (Fig. 2b), suggesting that *Scot-TravB* is an admixed population. The signal of admixture in *Scot-TravB* is reflected in the increased southern Scottish/north-eastern Irish ancestry inferred for this group through identity-by-descent, a similar signal to the fact that there is a substantial ancestry component in *Scot-TravB* that is not present in *Scot-TravA*. The branching of *Scot-TravB* within the reference Mainland British and Irish genetic branches in the fineSTRUCTURE tree (Fig. 2a) also suggests admixture with similar groups, whereas *Scot-TravA* forms a distinct clade, indicating greater genetic differentiation from the settled populations. Furthermore, F_ST_ estimates show that the difference between *Scot-TravB* and the settled populations are at levels (~0.0018) we have previously observed between genetic clusters among the reference British and Irish populations^42,43^. In contrast, *Scot-TravA*, exhibits greater genetic distance from these populations with a higher mean F_ST_ value (0.006), similar to that observed between Scotland and Italy^59^, and suggesting the cluster has experienced a large amount of genetic drift.

Full Scottish Travellers have a considerable burden of long autozygous segments in their genomes – over 30 times more than the Mainland British and Irish reference populations. The genomic inbreeding coefficient, F_ROH1.5_, is similar to that observed in the Pakistani community in Bradford, England^45^, consistent with frequent cousin marriage among an endogamous community, as suggested by the pedigree structures and elevated isonymy. The signals differ substantially between the *Scot-TravA* and *Scot-TravB* groups, with *Scot-TravA* being the most autozygous, and also the most consanguineous, as measured by F_IS_ – again mirroring the Bradford Pakistani community. Focussing on longer ROH, for instance those above 10 Mb in length, corroborates that much of this burden (mean sum 59 Mb in full *Scot-TravA*) has arisen from recent consanguinity^60^. Full Traveller members of *Scot-TravB*, on the other hand, have a mean (18 Mb) ~ 3 times lower than *Scot-TravA*, but 11 times that of the Mainland British and Irish reference populations, and comparable to Irish Travellers and European Roma^59^.

Lastly, model-based demographic reconstruction further supports this picture. Analysis of trajectories of effective population sizes indicates that both *Scot-TravA* and *Scot-TravB* historically had low effective population sizes and underwent recent demographic bottlenecks. The reduction in N_e_ and bottleneck strength is higher in *Scot-TravA* than *B*, and both are stronger and considerably more recent (estimated to be 7-9 generations ago) than bottlenecks in the history of the Northern Isles of Scotland.

The recorded Scottish Traveller history is, of course, sparse. However, among the traced pedigrees, we note a number of examples of Scottish Traveller bride-groom surname pairs involving McPhee, Townsley, Newlands, Whyte and Stewart from the 1760s-90s, evidencing the existence of within-community marriage (endogamy) by that time. This information was captured in the death certificates of their children who survived until after the onset of Scottish civil registration of deaths in 1855.

In addition to the strong identity-by-descent relationships and cohesive clustering on multidimensional representations of autosomal variation, mitochondrial DNA provides further evidence for a common origin of both Scottish Traveller groups, with sharing of three founder haplotypes. Scottish Travellers also show the classic signature of an isolated population, where a small number of mitochondrial founder lineages account for a large proportion of the population, in this case, 64%. This is similar to that seen in the Iberian Roma (65%) and the 75% estimated for Ashkenazi Jews^61^.

While we have noted a level of fluidity in reported identities between Highland and Scottish Traveller grandparents, *Scot-TravA* includes more individuals with grandparents identifying as Highland Travellers, and/or coming from the northern half of Scotland, while *Scot-TravB* includes fewer such individuals and more grandparental origins in the Lowlands of Scotland, leading to the hypothesis that the *Scot-TravA* cluster predominantly captures a Highland Traveller-like ancestry while *Scot-TravB* captures a Lowland Traveller-like ancestry. Indeed, one *Scot-TravB* individual reports partial Lowland Traveller ancestry.

Overall, our results suggest a predominantly indigenous Scottish origin for the Scottish Travellers, followed by a shared history of isolation and subsequent differentiation over the centuries, with differing levels of admixture with surrounding populations. These differences may have arisen because of differing conditions across Scotland, or variability in the strength of the proscription of marriage between Travellers and settled people in different areas. Increased genetic drift through greater reduction in population size is seen for the *Scot-TravA*, likely Highland Traveller, group.

While we sampled very few Irish Travellers and English Gypsies, we were able to confirm the Irish origins of the Irish Travellers^59^ and show that both groups were distinct from each other and the Scottish Travellers. Some English Gypsies showed autosomal evidence for minority European Roma ancestry. However, the H7a1a mitochondrial lineage observed in three English Gypsies matches a Spanish Roma sample, and is one step away from two Lithuanian Roma samples; indeed, H7a1 has been recognised as a Roma founder lineage, as has U3b1c^62^. The situation is similar to that reported for the Welsh Kale (who are a Romany group), who have little evidence of Roma ancestry in autosomal analyses, yet some carry South Asian (and hence Roma) mitochondrial lineages^63^. We therefore provide evidence for partial European Roma ancestry among the English Gypsies, which is potentially maternally biased.

Interestingly, the second pattern of Scottish Traveller mtDNA phylogeography, with lineages branching among Southern and Eastern European haplotypes, but not matching any European Roma lineages, contrasts with the fact that there is no autosomal signal of Continental European or Roma ancestry. This may be an analogous situation to that in the Welsh Kale and English Gypsies, where there is little or no detectable European Roma autosomal ancestry, yet mitochondrial lineages have preserved evidence of ancient Mediterranean maternal contributions to the Scottish Traveller gene pool.

In light of this inferred population history of the Scottish Travellers, we expected an enrichment of rare, clinically significant variants within the population^52,56,63–65^ Whole-exome sequencing (WES) of 123 participants revealed 166 mostly autosomal recessive disease-causing variants associated with severe phenotypes. Most of these variants were singletons (*n* = 119) observed in the heterozygous state among participants.

Despite the small sample size, we identified five pathogenic or likely pathogenic (P/LP) putative founder variants with notable enrichment in the Scottish Traveller population, each occurring in at least three individuals (at *PIEZO1*, *DOK7*, *HOGA1*, *CANT1* and *CRB2*). These variants demonstrated from ~30 to >2000-fold frequency increases compared to non-Finnish European populations, where their frequencies range from 0 to 0.001 in gnomAD. Two further variants (at *VWF* and *ASL*) were increased in frequency by ~15-fold. Interestingly, none of these founder variants were located in genes included in the ACMG list for reporting secondary findings in clinical exome or genome sequencing^55^. However, actionable variants in the *BTD* and *HFE* genes were identified, exhibiting frequencies comparable to those in non-Finnish Europeans, as did the most common pathogenic cystic fibrosis variant. We observed a high individual burden of rare P/LP variants in the Scottish Travellers, due to the multiple variants which have drifted to higher frequencies; indeed 84% of individuals carried two or more different recessive actionable variants, and 13% carried five or more. In the Irish Traveller samples, we observed five clinical variants previously reported from that population^31^ and 18 variants hitherto not reported among Travellers. A pathogenic haplotype at *THOC6* is likely to be a founder variant among Irish Travellers.

Our results show that it is vital to characterise and catalogue the list of inherited disorders in the Scottish Travellers. In particular, a high number of normally rare recessive variants in our participants—some reaching over 5% carrier frequency—together with the elevated levels of autozygosity: Scottish Travellers with four Traveller grandparents are on average as homozygous as the offspring of first cousins once removed (F_ROH_ = 3.8%)—combine to greatly increase the risk of recessive genetic diseases in the population. The paucity of genetic and biomedical studies about the community is likely to further negatively impact health outcomes for the Scottish Travellers. Given the historic and systemic discrimination they have faced, the Traveller community already has the worst outcomes in education, health, employment, and criminal justice in Scotland^29,66^. In order to prevent further disparities from arising as genomic medicine enters standard practice in the mainstream population, there is therefore an opportunity to engage with this population regarding a bespoke community-based reproductive carrier screening programme, similar to J-netics^67^, a charity organisation in the UK whose purpose is to prevent and diagnose 47 predominantly Jewish recessive disorders, and raise awareness about them. In the meantime, clinicians who care for Scottish Travellers should be made aware of the predicted enrichment for the five most uplifted disorders in Table 2, and the scope for otherwise rare recessive Mendelian diseases generally to be more prevalent than is usually the case in Scotland.

In conclusion, this study provides confirmation of an indigenous Scottish origin for the Scottish Traveller population, offering a comprehensive analysis of their population genetic structure and demographic history using dense genome-wide genotype data. We identified genetic substructure within the community, observed elevated levels of autozygosity, strong founder effects on mtDNA lineages and detected multiple, otherwise rare, recessive variants associated with severe disorders that have drifted to high frequencies. Scottish Travellers therefore take their place along with other indigenous non-Romany Traveller groups in Europe, such as the Woonwagenbewoners of the Netherlands, the Yenish of Germany and surroundings^68^, and the Irish Travellers.

These findings provide insights into understanding the genetics of the Scottish Traveller founder population. The evidence of both community engagement with genetics through the Traveller Genes study and the discovery of a number of pathogenic founder variants suggests an opportunity and also the need to translate these population-specific insights into educational and screening initiatives to prevent further health disparities and ensure genomic medicine is equitably delivered to all.

## Methods

### Ethical approval

The Traveller Genes study was given a favourable opinion by the NHS NRA London - London Bridge Research Ethics Committee (21/PR/1229), as well as Research and Development (3021/0222), and Caldicott Guardian (CRD21123), NHS Lothian. All participants gave written informed consent for research into health and ancestry.

### Participant recruitment, DNA extraction and QC

Participants (*n* = 148) with at least two grandparents self-reported to be of Traveller ancestry were recruited after public announcements. Traveller membership was broadly defined, including those who identify as Scottish Travellers (including Highland Travellers, Lowland Travellers, Scottish Travellers), Irish Travellers, Romanichal or Romany Travellers, English Gypsy or Welsh Kale communities. The data dictionary is at: 10.7488/ds/3155. After giving consent online and completing a short questionnaire, saliva samples (Oragene) collected from the participants were processed and stored using standard operating procedures and managed through a laboratory information management system at the Edinburgh Clinical Research Facility, University of Edinburgh. The protocol followed closely that for Viking Genes^69^. Whole-exome sequencing was performed at Regeneron Genetics Centre. To provide a large SNP overlap with existing British and Irish reference datasets, 48 samples were chosen for genotyping on the Illumina OmniExpress chip at the Edinburgh Clinical Research Facility. This OmniExpress dataset (henceforth SNP-array data) included all volunteers with four Scottish Traveller grandparents or with four Irish Traveller grandparents (n = 29), the remainder being made up of Travellers with fewer Traveller grandparents.

Pedigrees were traced using civil records of birth, marriage and death available from ScotlandsPeople, in conjunction with data available from family members.

### Exome sequencing

Exome sequencing was performed at the Regeneron Genetics Centre using an automated sample preparation approach. Briefly, DNA libraries were created by enzymatically shearing DNA to a mean fragment size of 200 bp, and a common Y-shaped adapter was ligated to the ends of the fragments. Unique, asymmetric 10-bp barcodes were added to the DNA fragments during library amplification to facilitate multiplexed capture and sequencing. Samples were captured with Twist Bioscience’s Comprehensive Exome Panel, which targets 33 Mb of the human genome, including all protein-coding regions and UTRs, and also with Regeneron’s panel of genotyping-by-sequencing probes. Captured libraries were sequenced on Illumina sequencers using 75 bp paired-end reads and two index reads.

Genotypes were called using a custom pipeline that combines Google’s DeepVariant genotyper (for the analysis of exome sequencing data), GATK’s HaplotypeCaller (for calculation of genotype likelihoods at common variants that overlap genotype-by-sequencing probes), and GLIMPSE software (for genotype refinement using a reference panel of haplotypes). DeepVariant was run with customized parameters tailored to Regeneron Genetics Centre exome sequence data. GATK and GLIMPSE were run with default parameters. Samples were excluded based on duplicates, low coverage, mismatched sex, contamination/heterozygosity, and genotyping discrepancies. The joint genotyping tool, GLnexus, was used for project-wide VCF files to filter variants based on stringent read depth (DP) and allele balance (AB) thresholds.

### Reference datasets

We combined genotype data from individuals of British and Irish ancestry from a number of studies to create a dataset with comprehensive sampling across the UK and Ireland: the Generation Scotland study^70^ (491 Scots, 160 Irish), ORCADES^71^ (111 Orcadians), VIKING I^72^ (172 Shetlanders), SCOTVAR^73^ (34 from Western Scotland), Avon Longitudinal Study of Pregnancy and Childhood in the Isle of Man (ALSPAC IOM) study^74^ (40 Manx), the Irish DNA Atlas^42^ (350 Irish), the Trinity Student Study, TSS^75^ (2,232 Irish), and an updated version of the People of the British Isles dataset, PoBI^39^ with additional genotypes (3568 English, Welsh, Scottish and Northern Irish) after excluding duplicate samples, non-British ancestry samples, and genotypes from urban areas. Genotypes for Iberian Roma^76^ were available and used to represent European Roma populations.

### Quality control and phasing

Using PLINK (ver. 1.90b)^77^ and Traveller Genes SNP-array data, we first processed individual datasets by excluding any strand-ambiguous SNPs and included only autosomal SNPs. We combined the references with the Traveller Genes genotype data using the --merge flag in PLINK (ver. 1.90b)^77^. We removed SNPs with minor allele frequency (MAF) < 2%, missingness >5%, and a Hardy-Weinberg Equilibrium (HWE) *p* value < 1×10^−6^. We excluded individuals with SNP missingness >5%. Pairs of related individuals (3rd degree and closer) were identified using KING (ver. 2.2)^78^ and one random individual from each pair was removed. To calculate an unlinked PCA, we utilised a set of SNPs that were pruned with respect to linkage disequilibrium using the PLINK command --indep-pairwise 1000 50 0.2, performing PCA using the --pca PLINK command. After this quality control, the Traveller Genes genotype data included 44 samples and 606,114 markers while the combined dataset of Travellers and British-Irish references had 4,535 samples and 336,530 markers.

Unphased genotypes from this combined dataset were converted to VCF files using PLINK and separated by autosome. Using SHAPEIT v.4^79^, we phased the samples with default settings and the GRCh37 build genetic map for recombination distances generated from HapMap. Identical-by-descent (IBD) segments ≥1 cM in length were detected using hap-ibd v 14/06/2023^80^. The extent of IBD shared between pairs of individuals was estimated by calculating the total length of IBD shared and the total number of IBD segments. After excluding regions with high IBD sharing (defined as more than 3 standard deviations from the genomic mean IBD coverage), we generated summary statistics for every genetic community for the reference populations and the fineSTRUCTURE clusters for the Scottish Travellers. Additionally, we ran the phased haplotype data through pbwt-paint v 14/06/2023^37^ to generate a coancestry matrix, and then generated principal components from the data as calculated by the *prcomp* function in R v4.4.2

### Population genetic structure and relatedness

Using the summarised IBD data, we first constructed a network graph using every individual as a node and the total length of autosomal IBD segments from 1 to 120 cM shared between a pair of individuals as the edge length (igraph package v2.1.4 in R^81^). We exclude oversharing outliers to account for cryptic relatedness, excluding connections with a sum of all segments ≥99.995 percentile of IBD length. The Leiden community detection algorithm^40,41^ was then applied on this network. We used the *rleiden.community* function within the leidenAlg v1.1.5^41^ package in R with a *max.depth* of 3 (i.e. 3 recursions of the clustering process) and a *min.community.size* of 20 (i.e. communities of 20 individuals were not divided in a subsequent recursion). The communities were labelled to reflect their geographic or self-declared origins.

To avoid biases arising from over-representation of one population or ancestry over another, for fineSTRUCTURE v2^38^ analyses, we created a balanced subset of the merged phased data: we randomly subsampled ~40 samples each from the reference populations (*n* = 260 samples from Scotland, Ireland, England, Wales, Orkney, Shetland and the Isle of Man, together with 36 Traveller participants with at least 2 Traveller grandparents), so that there was equal representation of participants from populations of interest. We then ran CHROMOPAINTER^38^ on the balanced dataset to generate the co-ancestry matrix that captures pairwise shared haplotype chunk data. CHROMOPAINTER was run with default settings, with the exception of specifying the number of ‘chunks’ per region to 50, similar to other analyses that have found British and Irish samples share longer IBD haplotypes than is common in other parts of the world^42,43^. We perform PCA on the co-ancestry matrix. We ran fineSTRUCTURE’s MCMC analysis on the co-ancestry matrix output from CHROMOPAINTER; with 500,000 burnin iterations, and default values for sample iterations and thinning intervals, and finally performed 50 tree-building iterations. The resulting fineSTRUCTURE clusters were analysed, and we combined small uninformative clusters together.

We investigated the genetic relatedness between the Travellers and the reference populations using a modification of a non-negative least squares (NNLS) regression-based method to estimate ancestry profiles^39^. Using the pairwise IBD shared as the input, we estimated haplotype sharing profiles for each reference Leiden community and Traveller fineSTRUCTURE cluster. Specifically, we modelled each Traveller cluster as a target cluster, estimating the proportion of haplotype sharing donated from P source clusters, where we treated every reference Leiden community as a potential source. For further details about the calculations, please refer to Gilbert et al.^82^.

### Demographic history

To estimate genetic isolation and consanguinity in the Traveller genetic communities, we estimated runs of homozygosity (ROH) within every genetic community using the PLINK --homozyg algorithm, with a minimum SNP count of 50, a scanning SNP widow size of 50 SNPs, minimum length of 1500 kb, maximum internal gap of 1000 kb length, a maximum inverse density of 50 kb/SNP, maximum missing genotypes in the scanning window of 5, and a maximum number of heterozygous genotypes in the scanning window of 1.

We estimated the F_ST_ statistic, and the F_ROH_^45^ and F_IS_ statistics for every Traveller fineSTRUCTURE cluster and reference genetic communities. We also stratified the Travellers by their number of Traveller grandparents, and then estimated ROH levels per group. Additionally, we calculated the total length and number of IBD segments shared between pairs of individuals in the dataset. Summary statistics were generated for every pair of reference genetic communities and the Traveller fineSTRUCTURE clusters.

We tested for admixture in the Scottish Traveller genetic clusters by calculating f3 statistics with genetic clusters of reference British-Irish populations and the Scottish Traveller clusters as parent populations using ADMIXTOOLS v2^83^. Admixture graphs were then generated using the second level clusters of reference populations and the Scottish Traveller clusters. To investigate any signals of population bottlenecks, effective population sizes (N_*e*_) were estimated using IBDNe v 23/04/2020, on the IBD data from Travellers. We selected IBD segments ≥4 cM shared between pairs of individuals within the fineSTRUCTURE Traveller clusters and then ran IBDNe with *nboots* = 100 to generate confidence intervals of the N_*e*_ estimates. For comparison, we performed the same analysis on the English, Scottish, Orcadian, and Shetlandic genetic communities. We confirmed the bottleneck signals in the Scottish Traveller clusters using ASCEND v10.1.1 —we estimated the age and intensity of the bottlenecks with the default parameters. Fifteen random samples from (a) the 1000 Genomes project, and (b) a combination of the 1000 Genomes project and the reference British-Irish dataset were used as outgroups.

### Mitochondrial DNA analysis

We generated a maximum parsimony (MP) phylogenetic tree using 121 complete mitogenomes belonging to 67 Scottish individuals with Traveller ancestry on the maternal line of descent. These included 62 Scottish/Highland Travellers and five Lowland Travellers; we also included 14 English Gypsies, two English Romanichals, and eight Irish Travellers (Supplementary Data 7, Supplementary Data 11). The remaining 30 mitogenomes derive from settled individuals from Scotland (*n* = 16), England (*n* = 12) and Ireland (*n* = 2).

Haplogroup nomenclature follows PhyloTree database build 17 (at http://www.phylotree.org/)^84^, and has been revised according to Haplogrep 3^85^. Variants are scored *vs* the revised Cambridge Reference Sequence (rCRS)^86^, and are shown on the branches. These are transitions unless the base change is explicitly indicated. The suffix “@” designates reversions *vs* rCRS, underlined positions represent parallel mutations that appear more than once on the phylogenetic tree. Prefixes d, and ins indicate deletions, and insertions, respectively. The following positions have been excluded from the tree construction: np 309-310, np 315, insertions and deletions at np 522-525, transversions at np 16182 and 16183, and insertions at np 16,192 and 16,193, if the m.16189 T > C is present, and np 16519.

We choose a phylogeographic approach to investigate possible matrilineal links between the Traveller groups and other European populations (see Supplementary Information). We built maximum parsimony (MP) trees for all relevant haplogroups (Supplementary Data 11–13).

### Clinical variants analysis

To identify carriers of pathogenic and likely pathogenic (P/LP) variants in the Scottish Traveller dataset, we modified the pipeline described by Kerr et al.^52^. Briefly, we extracted all variants that mapped to genes listed in:

A. the ACMG v3.2 list of 81 genes in which actionable secondary findings are recommended to be returned^55^
B. the list of genes from a catalogue of inherited disorders found in the Irish Traveller community^31^
C. a list of all of the remaining known coding genes (excluding those in categories A and B above).

We then annotated these variants with the ClinVar data (variant_summary.txt.gz from ClinVar in August 2024) to determine their pathogenicity and their review status on ClinVar. This list of variants was further filtered to include only variants annotated as P/LP, that had star status (weight of evidence), 2* or greater in ClinVar (multiple submitters, no conflicts). Homozygous and heterozygous carriers of these variants were then extracted from the exome sequence VCF files. The ClinVar-exome pipeline is available on GitHub: https://github.com/vikinggenes/clinvar_pipeline.

These data were further annotated with gnomAD (ver.2.2)^87^ non-Finnish European (NFE) frequencies. We estimated the minor allele frequencies (MAF) for the Scottish Travellers and Irish Travellers using Traveller genome equivalents. Participants with 4 Traveller grandparents contribute one genome equivalent, whereas those with 3 or 2 Traveller grandparents contribute 0.75 or 0.5 genome equivalents, respectively (which assumes for these otherwise vanishingly rare variants that none come from the non-Traveller component in these mixed individuals). We identified pathogenic variants enriched in the Travellers by calculating the uplift or fold change in the frequencies in the Travellers in comparison to non-Finnish Europeans.

### Reporting summary

Further information on research design is available in the Nature Portfolio Reporting Summary linked to this article.

## Supplementary information

Supplementary Information

Description of Additional Supplementary Files

Supplementary Data 1

Supplementary Data 2

Supplementary Data 3

Supplementary Data 4

Supplementary Data 5

Supplementary Data 6

Supplementary Data 7

Supplementary Data 8

Supplementary Data 9

Supplementary Data 10

Supplementary Data 11

Supplementary Data 12

Supplementary Data 13

Reporting Summary

Transparent Peer Review file

## Data Availability

There is neither Research Ethics Committee approval, nor consent from participants, to permit open release of the individual level research data underlying this study. Instead, the research data are available through managed access, after application to each population cohort. For Traveller Genes, information is provided at https://traveller-genes.ed.ac.uk/our-research/access. All requests by researchers are reviewed by the Traveller Genes Data Access Committee and each approved project is subject to a data or materials transfer agreement, or commercial contract with the University of Edinburgh. The exome sequencing data generated in this study are also available through the same managed access process to non-commercial researchers. Information and samples from volunteers can only be used by researchers if there is relevant scientific and ethical approval for the research. We are happy to consider requests from researchers working in countries outside the UK, or in commercial companies. Personal volunteer details (such as names, email or postal address) will never be shared with researchers. Please contact TravellerGenes@ed.ac.uk for more information. The expected time-frame for responses is two weeks and data will be available for a two-year period. Please contact Prof Gianpiero Cavalleri (gcavalleri@rcsi.ie) to access the Irish DNA Atlas genotype data^42^. For the other datasets used in this study, please see the published articles for details; Trinity Student Study dataset^75^, the People of the British Isles dataset^39^, Avon Longitudinal Study of Pregnancy and Childhood in the Isle of Man study^74^, ORCADES^71^, SCOTVAR^73^, Generation Scotland^70^, and VIKING I^72^. The Iberian Roma data are available from doi.org/10.6084/m9.figshare.7594730 and the 1000 Genomes Project data from https://www.internationalgenome.org/data-portal/data-collection/phase3. All other data supporting the findings of this study are available in the article or in supplementary files.
