# Supplementary material for: Genetic ancestry and monogenic disease risk in the Scottish Traveller founder population
Source: Nat Commun. 2026 Jul 15;17:5843. doi: 10.1038/s41467-026-74969-y (PMC13373179; doi:10.1038/s41467-026-74969-y)
Supplement: Supplementary file 1 — Supplementary Information [file 41467_2026_74969_MOESM1_ESM.pdf]

Supplementary Information.

Shanmugam, A. *et al*. Genetic ancestry and monogenic disease risk in the Scottish Traveller founder population. *Nature Commun* (2026)

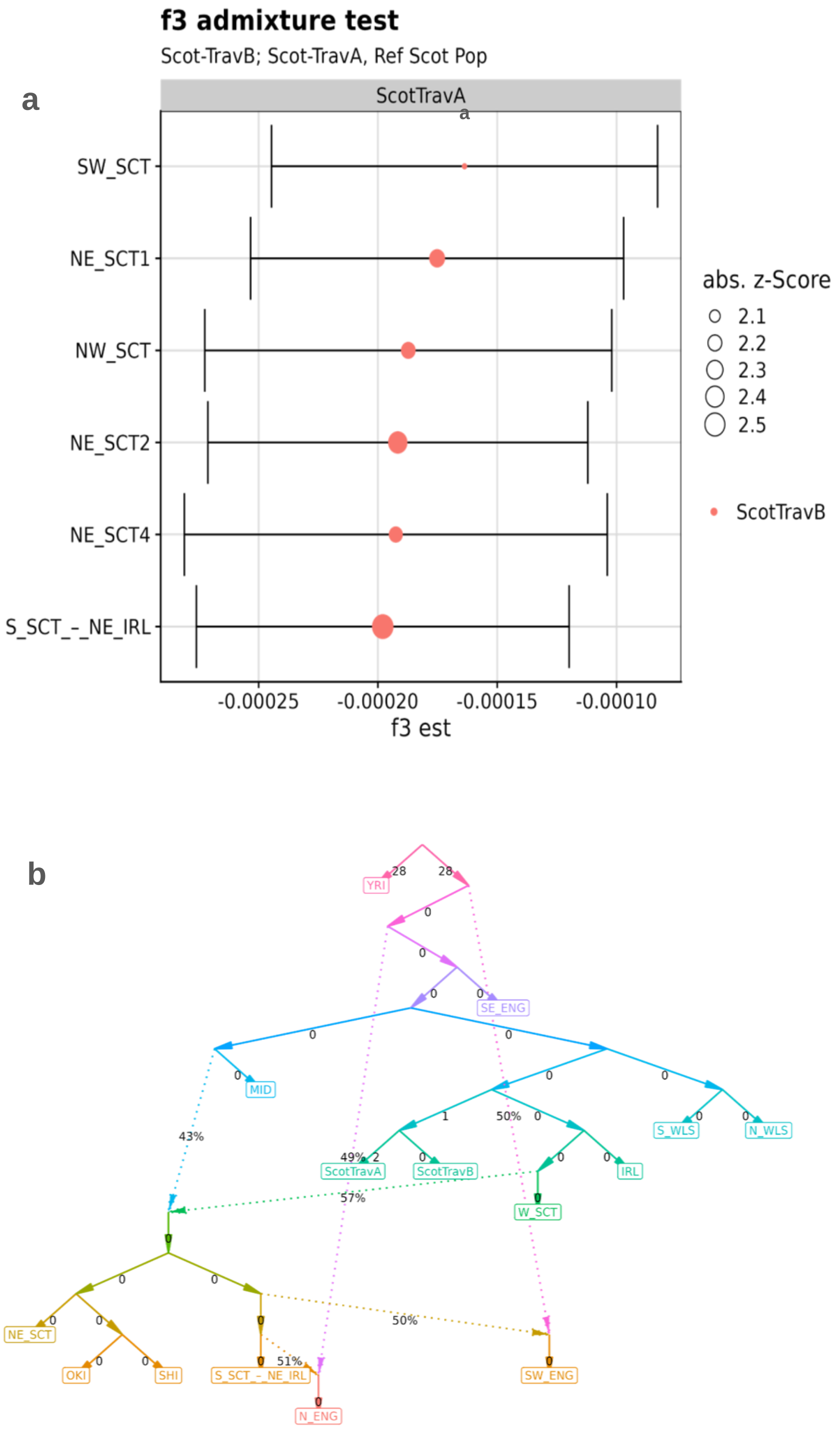

Supplementary Figure 1 Admixture test of Scot-TravB. (a) The plot depicts the f3 values calculated to test if Scot-TravB is an admixture between Scot-TravA and settled Scottish genetic communities (y-axis). The size of the point indicates the z-score. While non-significant, the f3 values suggest that Scot-TravB is admixed between Scot-TravA and settled Scottish populations. (b) An admixture graph generated using *qpGraph* shows that Scot-TravA and Scot-TravB group together, and are a part of a clade with west Scottish and Irish genetic clusters.

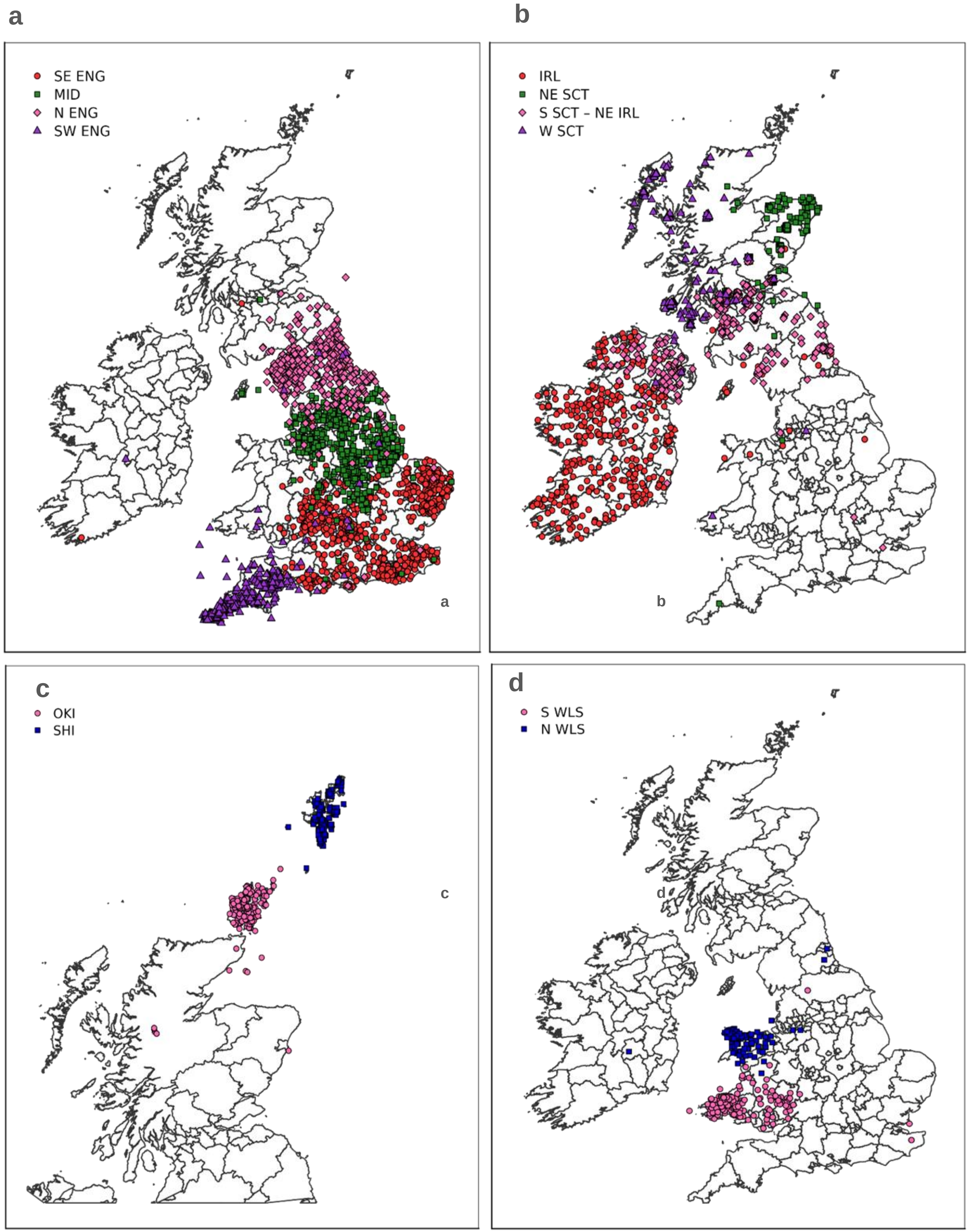

Supplementary Figure 2 Genetic communities detected in British and Irish reference populations. Using the Leiden community detection algorithm, we show the 2nd level communities detected in (a) England (b) Scotland and Ireland, (c) the Northern Isles and (d) Wales. The colours for the points have been re-used and indicate the 2nd level genetic communities and the maps show the geographic origin of samples.

a. Random outgroup from UK-IRL+ 1000 Genomes Project **b. Random outgroup from 1000 Genomes Project**

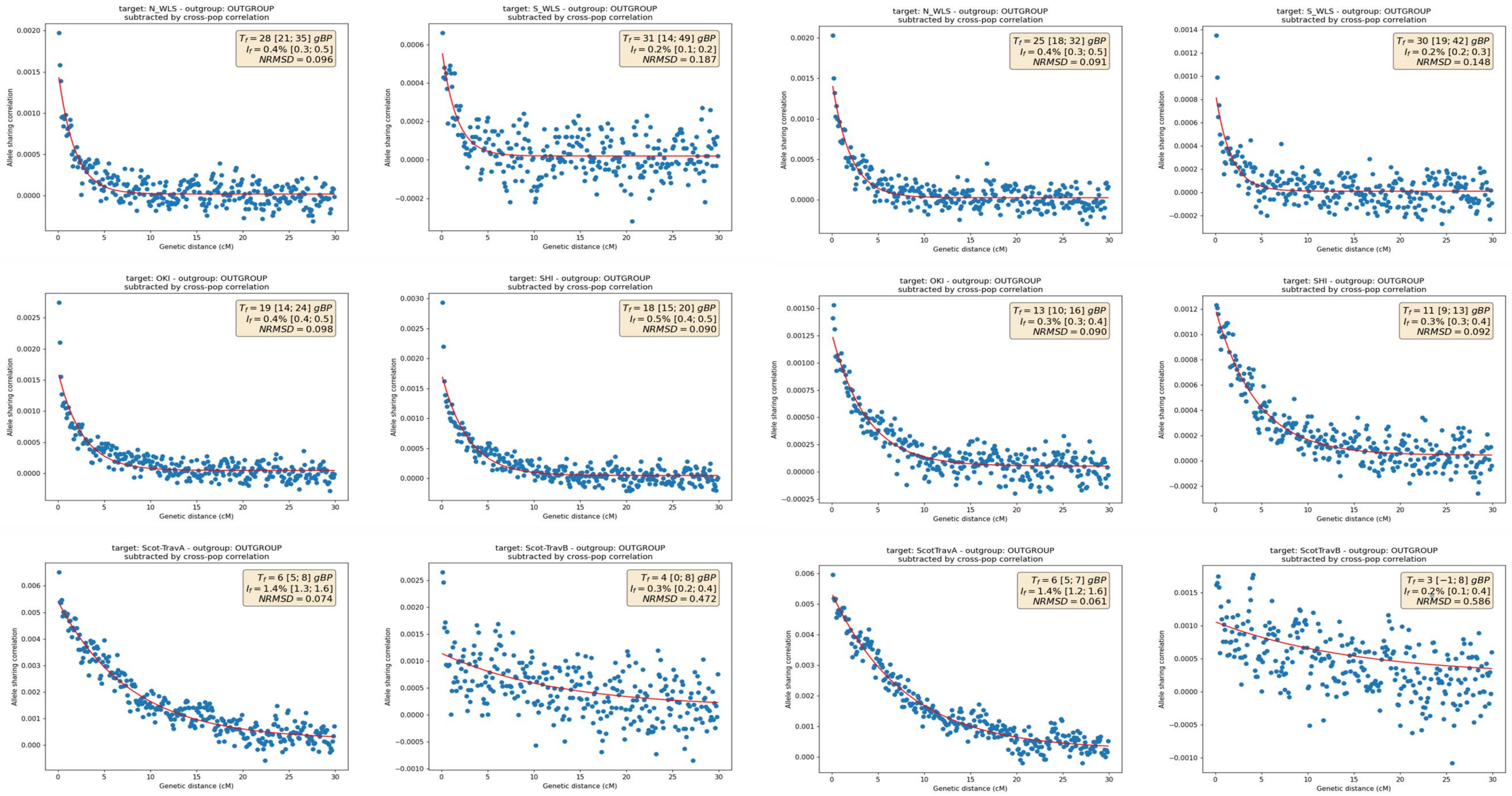

Supplementary Figure 3 Estimates of bottlenecks in known population isolates and Scottish Traveller genetic clusters. The figures show the decay of LD in the Orcadian (OKI), Shetlandic (SHI), Welsh (N.WLS and S.WLS), and the Scottish Traveller genetic clusters (Scot-TravA and Scot-TravvB). Fifteen random samples from (a) combination of genotypes from UK-Irish references and 1000 Genomes Project, and (b) just the 1000 Genomes project references. The estimates of age and intensity of bottlenecks were comparable in both cases.

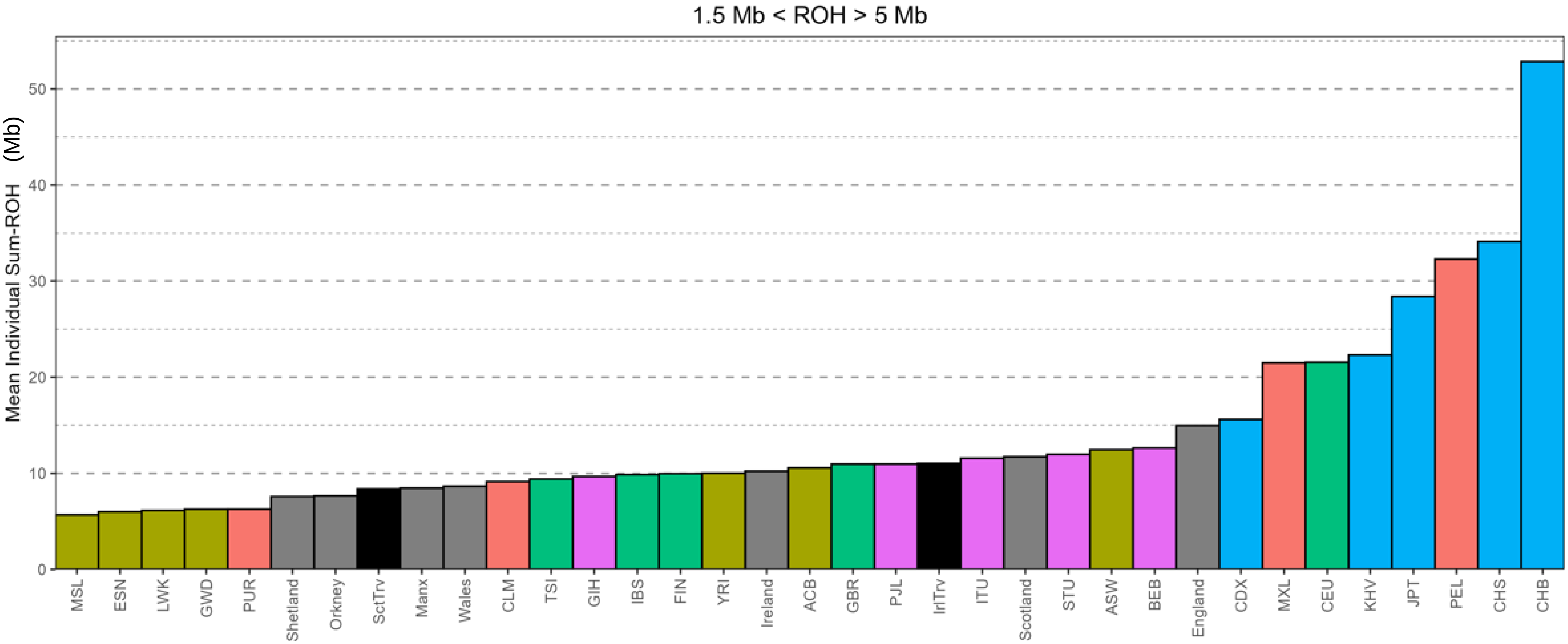

Supplementary Figure 4 Mean sum of runs of homozygosity (SROH) between 1.5 Mb and 5 Mb in length. ROH segments were detected using the PLINK --homozyg algorithm, processed in R, and visualised using the R package ggplot2. Bar colours and labels are as for Figure 4, as are sample sizes (see Supplementary Data 5).

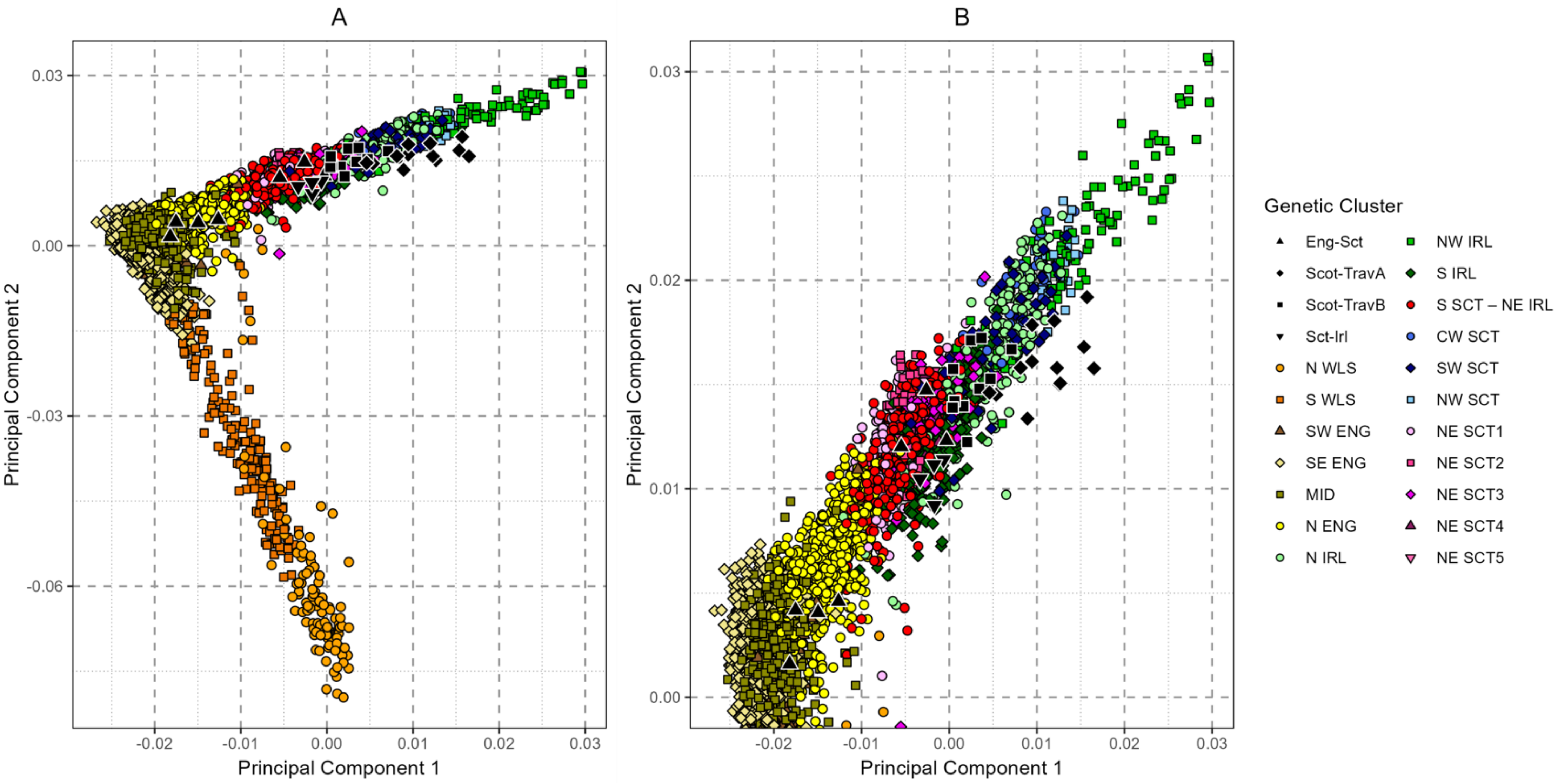

Supplementary Figure 5 Principal components analysis without islander individuals. (a) The first and second principal components of a pbwt-paint chunk counts matrix between British and Irish ancestry references and Traveller individuals. Individual points are colour-coded by the genetic clusters that they were assigned to. Black points represent Traveller samples grouped by fineSTRUCTURE cluster, with remaining individuals colour-coded according to their IBD-network cluster membership. The PCA was carried out in R, and the graph plotted using the ggplot2 R package.; (b) Detail of Anglo-Irish ancestry cline, showing overlap of Scottish Travellers with the Scottish part of the cline.

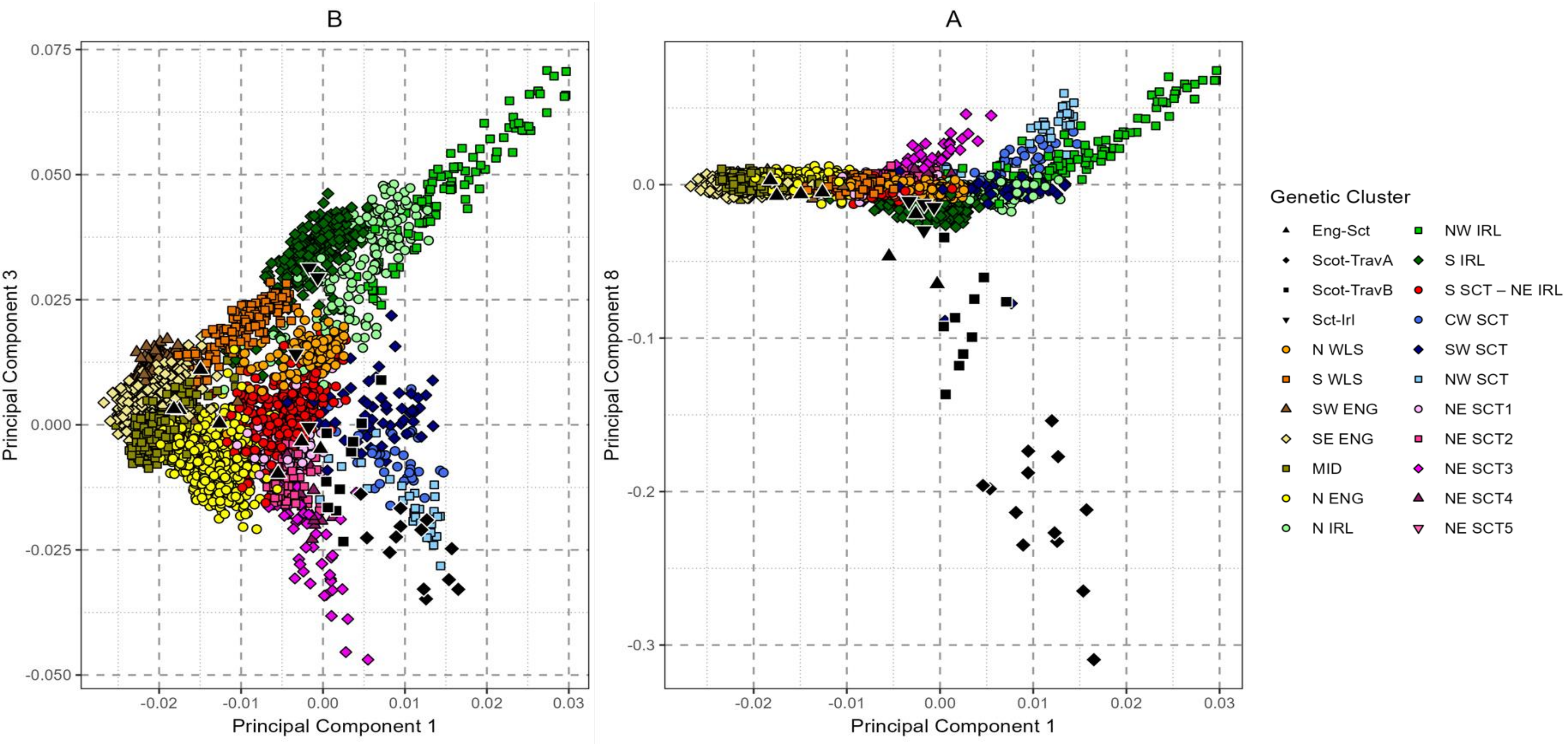

Supplementary Figure 6 Principal components analysis without islander individuals. (a) The first and third principal components of a pbwt-paint chunk counts matrix between British and Irish ancestry references and Traveller individuals. Individual points are colour-coded by the genetic clusters that they were assigned to. Black points represent Traveller samples grouped by fineSTRUCTURE cluster, with remaining individuals colour-coded according to their IBD-network cluster membership. The PCA was carried out in R, and the graph plotted using the ggplot2 R package.; (b) The first and eighth principal components.

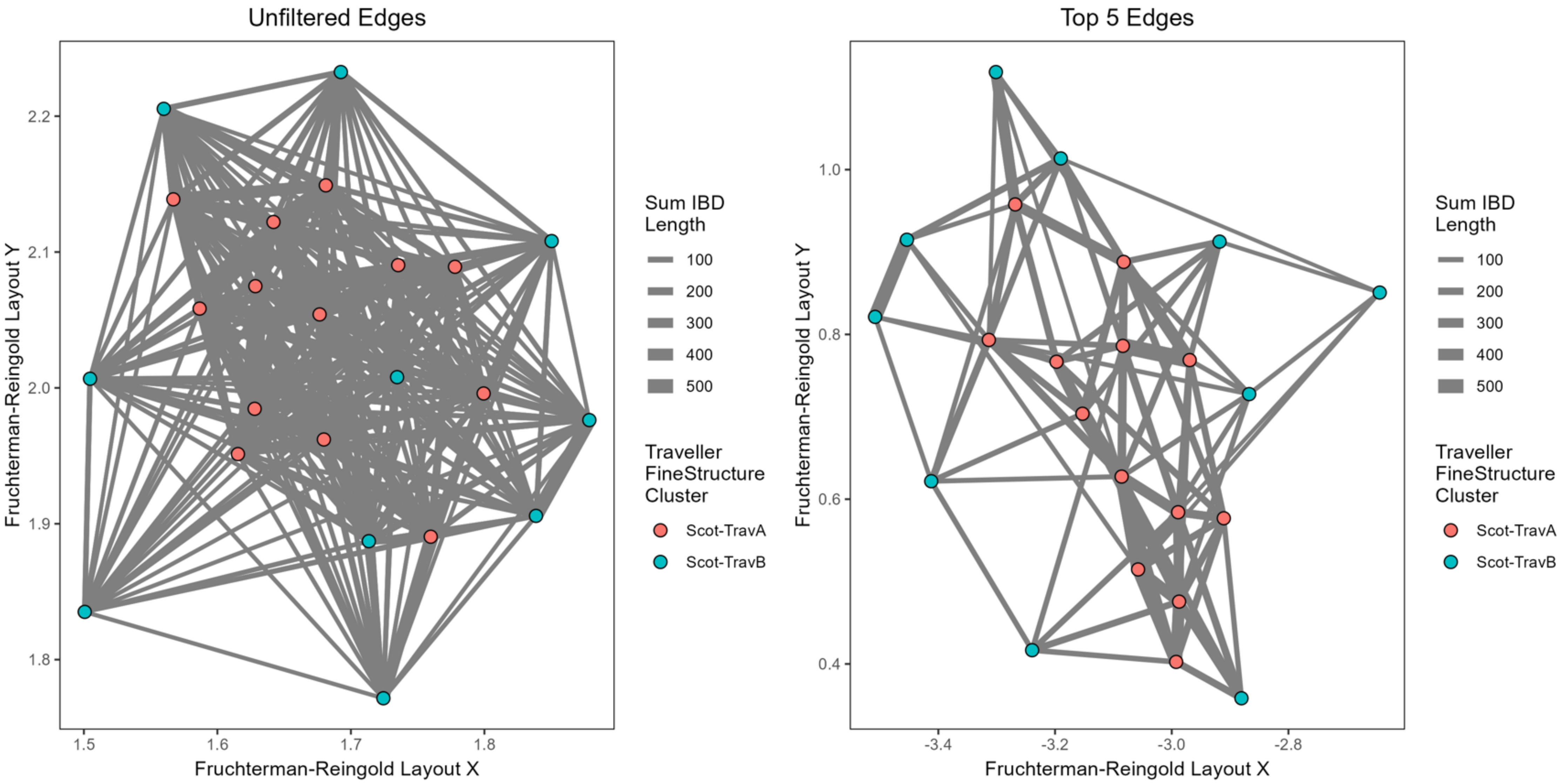

Supplementary Figure 7 Network representation of identity-by-descent sharing among Scottish Traveller samples placed in the Scottish Traveller fineSTRUCTURE genetic clusters. Each node (vertex) in the network graph represents one Scottish Traveller individual, with each connection (edge) representing the sum total of IBD segments from 1 - 120 cM in length that are shared between them. Network layout was achieved with the force-directed algorithm from Fruchterman-Reingold. Pairs of relatives closer than 2nd degree have been pruned. On the left is the layout when using every edge recorded. On the right is the layout when only the five edges with the greatest sum IBD length ("weight") for each node are used. Individuals are colour-coded by their fineSTRUCTURE cluster membership, and edge width is proportional to the sum IBD length shared between two Traveller individuals. The graph was created in R, using the igraph and ggplot2 R packages. Tight clustering (high IBD sharing) can be observed for members of the Scot-TravA cluster, with looser connections between members of Scot-TravB; and inter-cluster connections as well.

Supplementary Note 1 - Mitochondrial DNA analysis.

Approach. Taking into consideration the small sample size, we choose a phylogeographic approach to investigate possible matrilineal links between the Gypsy/Traveller and settled groups and other European populations (Supplementary Figs 4-6). We searched diagnostic mitochondrial DNA variants for all lineages identified among the Traveller groups using the allele search function on Mitomap (Lott et al., 2013) to find a list of published sequences matches available on NCBI, Nucleotide (Home - Nucleotide - NCBI). We extracted all available geographic information and compiled a dataset of 904 published mitogenomes belonging to the relevant haplogroups (Supplementary Table 8). We built maximum parsimony (MP) trees for all haplogroups with the exclusion of those that were either too widely distributed, the Traveller mitogenome too nodal or with no match to give any meaningful information (H2a1a1, J2a1a1, H1e1a, T1a, T1a1, and T2b). When the available published data was insufficient to identify any clear patterns in the phylogenetic data, we searched our in-house dataset of unpublished mitogenomes of European origin (N = 2628), including 670 mitogenomes from the British Isles, for further matches.

Local haplotypes. The phylogeographic analysis of the individual haplogroups shows that the majority of the haplogroups identified among the six traveller groups, including 75% of all samples, and 69% of all haplogroups, have direct or indirect matches in the local settled population, with shared haplogroups and haplotypes with published British, Irish, and Scottish mitogenomes (mostly from Orkney and Shetland, as these are over-represented in the Scottish data). 81% (54/67) of Scottish/Highland/Lowland Traveller samples are of local origin, as are 6/8 Irish Traveller individuals, but only 50% (7/14) of English Gypsy samples. These include haplogroups H16, H1bb, H1e1a, H1g1, H1i1, H1q1a, H31a, H5a1d, H6a1b4, H9a, I1a1b, I1a1e, I2, J1b1a1a, J1c2e, J2a1a1, K1a24a, K1c1b, K2a6, K2b1a1, T2b23a, T2b2b1, T2b3, T2b3b, T2b5, T2f1a1, U4a1b1, U4b1b1, U5a1c, U5a1b1a*, U5b2b1a1, U5b2b3b, W1c1, and X2c2 (Supplementary Tables 7 and 8). Trees have been generated for all haplogroups but H1e1a and J2a1a.

Among the “local” haplogroups, several display features of founder effect with multiple individuals sharing the same haplotype or very closely related haplotypes. The most frequent lineage is T2f1a1, defined by m.14319T>C. This haplogroup is carried by seven Scottish Travellers (17%), eight Highland Travellers (36%), and one Lowland Traveller (25%), thus representing 24% of all analysed traveller individuals from Scotland (Supplementary Table 7 & Supplementary Figure 5). Published mitogenomes belonging to T2f1a1 originate from the British Isles and Europe (with the single occurrence of a Uyghur from China). Among ancient samples, a single sample from Scotland matches the m.14319T>C Traveller branch, thus further supporting the local origin of this haplogroup (Supplementary Figure 5). Intriguingly, the oldest T2f1a1 mitogenomes belong to a Viking individual from Sweden (VK35.SG), dating back to 900-1200 CE (Margaryan et al., 2020).

A similar pattern is observable for K1c1b, which appears mostly centred in Northern Europe (Supplementary Figure 5), with two exceptions, one sample from Italy and one from Poland. However, the haplotype shared by the four traveller individuals from Scotland, defined by m.3736G>A, appears exclusive to individuals from Orkney and Shetland.

J1b1a1a appears to be specific to the British Isles, in particular Orkney and Shetland, but also England (including a medieval sample from London), Ireland, with a single match from Sweden (same haplotype as the Scottish Travellers), and several matches across samples from the US without further geographic information (Supplementary Figure 5). A search among published ancient genomes reveals matches among Vikings from Norway and the Faroe Islands (VK422.SG, and VK24.SG), Medieval Norway (VK113.SG).(Margaryan et al., 2020), and pre- and early Christian Iceland (SSG-A-4_38.SG, and TSK-A-26_38.SG) (Ebenesersdóttir et al., 2018).

U4b1b1 has a much wider distribution, with matches across Asia, Europe, Caucasus and the Near East (Supplementary Figure 5). Despite this wide distribution, the clade also includes several branches (U4b1b1a, U4b1b1b and the node defined by m.7939C>T & m.15760C>T)

which appear exclusive to Scottish individuals from Orkney and Shetland, and show founder effects there. Notably, none of these are a direct match to the haplotype shared by the four Traveller individuals from Scotland and one English Gypsy, which appears nodal and is only shared by a single mitogenome lacking geographic affiliation data.

Undefined haplotypes. The second largest set of haplogroups does not appear to have a clear geographic affiliation as these haplogroups are either too widely distributed or the haplotypes carried by the Traveller samples do not have matches either in published or unpublished data. These include H1ba, K1a3a3*, H2a2a1, T1a1, T1a1'3, and T2b. Trees have been generated for the first two haplogroups (Supplementary Figure 6).

Non-local haplotypes. Four haplogroups, H1c1+16093, H1bw, J2b1a1, and V2 appear to have a possible non-local origin, as they do not seem to have any obvious match in the local British or Irish mitochondrial variation, but seem to have either a stronger association with different Northern European populations, or populations from the Mediterranean area (Supplementary Figure 6).

In particular, H1c1+16093, carried by one English Gypsy, does not appear to have any matches among published or unpublished mitogenomes from the British Isles, but shares m.16093T>C with samples from other European populations, mostly Denmark (16 out of 24 samples), but also Germany, Poland and Italy (Supplementary Figure 6).

H1bw, carried by one Romanichal individual, includes samples from Iberia (Portugal, Spain, and the Canary Islands), Italy and Sardinia, Lithuania, and a single individual from Sweden. The Romanichal individual clusters with a French individual from Toulouse, and an Ashkenazi Jew individual of unknown European origin that shares the same haplotype with a Lithuanian (Supplementary Figure 6).

J2b1a1 carried by one Irish Traveller, includes samples from Italy (one individual from Umbria carrying the nodal haplotype, and one ancient individual dating to the Longobard period, from

Piedmont), Spain, France, Russia and even one sample from Tibet. This branch also includes two samples with known origin from Ireland and Scotland, but who belong to a separate branch, J2b1a1a, together with samples of unclear geographic origin. The Irish Traveller haplotype (defined by three variants: m.14178T>C, m.16291C>T, m.16304T>C) does not belong to this branch and in fact does not have any matches among the available mitogenomes (Supplementary Figure 6).

V2 detected among Scottish Travellers, includes all mitogenomes that do not fit in any of the currently identified V2 sub-branches (V2a-c). This heterogenous group includes mitogenomes from Italy, a French mitogenome directly related to one individual from Slovakia, a Belgian, and two branches showing evidence of founder effect restricted to the Canary Islands, and a Sardinian branch. The Scottish Traveller branch (represented by four samples) shows also evidence of founder effect but has no direct match with any available mitogenome (Supplementary Figure 6). There is a single occurrence of this haplogroup in the German Corded Ware (UNTA121_FK61), dating to 2906-2704 cal BCE (Mittnik et al., 2019).

Two haplogroups, exclusive to the travellers from Scotland, H1b1g, and U1a1a3, also seem to have a non-local origin but this time with more robust links to the Mediterranean area.

H1b1g, carried by one Scottish Traveller, is extremely rare. The tree includes only three additional samples, two from Central Italy (Umbria), while the third one, matching the Traveller haplotype defined by m.64C>T, has no available geographic origin (Supplementary Figure 6). However, search of H1b1g+64 in the FamilyTreeDNA public view dataset shows a match with an additional Italian sample. If correct, this would suggest a direct connection between the Scottish Traveller mitogenome and the Italian Peninsula.

U1a1a3 has a very different pattern, and although also rare, is a widely distributed haplogroup, including, in addition to the three traveller mitogenomes (two Scottish Travellers and one Lowland Traveller), four mitogenomes from Iran, two from Poland, and one, sharing m.16362T>C with the Traveller branch, from Spain (Supplementary Figure 6). There are three

matches to this haplogroup among published ancient genomes, a Kurgan from the North Caucasus (PG2002), dating to 2483-2342 cal.BCE (Wang et al., 2019), a Bronze Age sample from Meggido, Israel (I10771) (Agranat-Tamir et al., 2020), and one individual (I1955) from Iran dating to 1480-1640 calCE (Lazaridis et al., 2016).

Finally, two haplogroups, H7a1a, and U3b1c, previously described among European Roma, in particular Spanish (Aizpurua-Iraola et al., 2022; Gómez-Carballa et al., 2013; Martínez-Cruz et al., 2016), have also been detected exclusively among the English Gypsies pointing to a direct link with the Spanish Roma.

The three English Gypsies belonging to haplogroup H7a1a share the same exact haplotype (defined by m.3613C>T) with one Spanish Roma and two Lithuanian Roma. The branch also includes samples from Portugal, Italy, and ancient mitogenomes from Early Bronze Age Bulgaria, and Medieval Poland (Supplementary Figure 6).

The single English Gypsy individual carrying the U3b1c haplotype does not have a direct haplotype match but is well nested within a Romani branch defined by m.7759T>C, and including 47 Spanish Roma mitogenomes and a single Lithuanian Roma (Supplementary Figure 6).

Notably, none of the mitochondrial lineages (such as M5a1, M18, M25, and M35) typically associated with the South Asian origin of the Romani people are present among the Gypsy/Traveller samples from the British Isles (Ena et al., 2022; Gómez-Carballa et al., 2013).

References

1. Agranat-Tamir, L., Waldman, S., Martin, M. A. S., Gokhman, D., Mishol, N., Eshel, T., Cheronet, O., Rohland, N., Mallick, S., Adamski, N., Lawson, A. M., Mah, M., Michel, M., Oppenheimer, J., Stewardson, K., Candilio, F., Keating, D., Gamarra, B., Tzur, S., . . . Reich, D. (2020). The Genomic History of the Bronze Age Southern Levant. *Cell*, *181*(5), 1146-1157.e1111. https://doi.org/10.1016/j.cell.2020.04.024
2. Aizpurua-Iraola, J., Giménez, A., Carballo-Mesa, A., Calafell, F., & Comas, D. (2022). Founder lineages in the Iberian Roma mitogenomes recapitulate the Roma diaspora and show the effects of demographic bottlenecks. *Scientific Reports*, *12*(1), 18720. https://doi.org/10.1038/s41598-022-23349-9
3. Ebenesersdóttir, S. S., Sandoval-Velasco, M., Gunnarsdóttir, E. D., Jagadeesan, A., Guðmundsdóttir, V. B., Thordardóttir, E. L., Einarsdóttir, M. S., Moore, K. H. S., Sigurðsson, Á., Magnúsdóttir, D. N., Jónsson, H., Snorradóttir, S., Hovig, E., Møller, P., Kockum, I., Olsson, T., Alfredsson, L., Hansen, T. F., Werge, T., . . . Helgason, A. (2018). Ancient genomes from Iceland reveal the making of a human population. *Science*, *360*(6392), 1028-1032. https://doi.org/10.1126/science.aar2625
4. Ena, G. F., Aizpurua-Iraola, J., Font-Porterias, N., Calafell, F., & Comas, D. (2022). Population Genetics of the European Roma—A Review. *Genes*, *13*(11), 2068. https://www.mdpi.com/2073-4425/13/11/2068
5. Gómez-Carballa, A., Pardo-Seco, J., Fachal, L., Vega, A., Cebey, M., Martinón-Torres, N., Martinón-Torres, F., & Salas, A. (2013). Indian signatures in the westernmost edge of the European Romani diaspora: new insight from mitogenomes. *Plos One*, *8*(10), e75397. https://doi.org/10.1371/journal.pone.0075397
6. Lazaridis, I., Nadel, D., Rollefson, G., Merrett, D. C., Rohland, N., Mallick, S., Fernandes, D., Novak, M., Gamarra, B., Sirak, K., Connell, S., Stewardson, K., Harney, E., Fu, Q., Gonzalez-Fortes, G., Jones, E. R., Roodenberg, S. A., Lengyel, G., Bocquentin, F., . . . Reich, D. (2016). Genomic insights into the origin of farming in the ancient Near East. *Nature*, *536*(7617), 419-424. https://doi.org/10.1038/nature19310
7. Lott, M. T., Leipzig, J. N., Derbeneva, O., Xie, H. M., Chalkia, D., Sarmady, M., Procaccio, V., & Wallace, D. C. (2013). mtDNA Variation and Analysis Using Mitomap and Mitomaster. *Curr Protoc Bioinformatics*, *44*(123), 1.23.21-26. https://doi.org/10.1002/0471250953.bi0123s44
8. Margaryan, A., Lawson, D. J., Sikora, M., Racimo, F., Rasmussen, S., Moltke, I., Cassidy, L. M., Jørsboe, E., Ingason, A., Pedersen, M. W., Korneliussen, T., Wilhelmson, H., Buś, M. M., de Barros Damgaard, P., Martiniano, R., Renaud, G., Bhérer, C., Moreno-Mayar, J. V., Fotakis, A. K., . . . Willerslev, E. (2020). Population genomics of the Viking world. *Nature*, *585*(7825), 390-396. https://doi.org/10.1038/s41586-020-2688-8
9. Martínez-Cruz, B., Mendizabal, I., Harmant, C., de Pablo, R., Ioana, M., Angelicheva, D., Kouvatsi, A., Makukh, H., Netea, M. G., Pamjav, H., Zalán, A., Tournev, I., Marushiakova, E., Popov, V., Bertranpetit, J., Kalaydjieva, L., Quintana-Murci, L., Comas, D., & and the Genographic, C. (2016). Origins, admixture and founder lineages in European Roma. *European Journal of Human Genetics*, *24*(6), 937-943. https://doi.org/10.1038/ejhg.2015.201

10. Mittnik, A., Massy, K., Knipper, C., Wittenborn, F., Friedrich, R., Pfrengle, S., Burri, M., Carlichi-Witjes, N., Deeg, H., Furtwangler, A., Harbeck, M., von Heyking, K., Kociumaka, C., Kucukkalipci, I., Lindauer, S., Metz, S., Staskiewicz, A., Thiel, A., Wahl, J., . . . Krause, J. (2019). Kinship-based social inequality in Bronze Age Europe. *Science*, *366*(6466), 731-734. https://doi.org/10.1126/science.aax6219
11. Wang, C.-C., Reinhold, S., Kalmykov, A., Wissgott, A., Brandt, G., Jeong, C., Cheronet, O., Ferry, M., Harney, E., Keating, D., Mallick, S., Rohland, N., Stewardson, K., Kantorovich, A. R., Maslov, V. E., Petrenko, V. G., Erlikh, V. R., Atabiev, B. C., Magomedov, R. G., . . . Haak, W. (2019). Ancient human genome-wide data from a 3000-year interval in the Caucasus corresponds with eco-geographic regions. *Nature Communications*, *10*(1), 590. https://doi.org/10.1038/s41467-018-08220-8
