# Supplementary material for: Genetic ancestry and monogenic disease risk in the Scottish Traveller founder population
Source: Nat Commun. 2026 Jul 15;17:5843. doi: 10.1038/s41467-026-74969-y (PMC13373179; doi:10.1038/s41467-026-74969-y)
Supplement: Supplementary file 2 — Description of Additional Supplementary Files [file 41467_2026_74969_MOESM2_ESM.pdf]

Description of Additional Supplementary Information

File Name: **Supplementary Data 1**

Description: **Self-reported grandparental ancestries of Traveller Genes participants.** Ancestries are coded into different Gypsy/Traveller and settled groups, and the number of Traveller grandparents is given, along with an overall summary label and the proportion of Scottish Traveller ancestry.

File Name: **Supplementary Data 2**

Description: **Genetic distance (Fst) estimates between pairs of genetic clusters.** Hudson's Fst estimates are given for all pairwise comparisons of different ancestry groupings.

File Name: **Supplementary Data 3**

Description: **Cluster labels for the genetic communities.** Labels for the Leiden genetic communities are given for the first, second and third levels.

File Name: **Supplementary Data 4**

Description: **Genetic contributions from each reference genetic cluster to Scot-TravA and Scot-TravB in NNLS analysis.** Abbreviations are given in Supplementary Data 3.

File Name: **Supplementary Data 5**

Description: **Burden of homozygosity in different Scottish Traveller groupings.** Figures are given for sum and number of runs of homozygosity (ROH) and Fis, a measure of inbreeding. Includes abbreviations and sample sizes for Fig. 4a (and Supplementary Fig. 4).

File Name: **Supplementary Data 6**

Description: **Effective population size (Ne) estimates over the last 50 generations.** Estimates with 95% confidence intervals are given for England (ENG), Orkney (OKI, Scotland (SCT), Shetland (SHI), and Scottish Traveller subgroups A and B.

File Name: **Supplementary Data 7**

Description: **Mitochondrial haplogroups and reconstructed haplotypes for Traveller Genes participants.** See Supplementary Information for details.

File Name: **Supplementary Data 8**

Description: **Published mtDNA haplotypes in the Traveller Genes sample haplogroups and used for tree building.** See Supplementary Note 1 for details. Each sheet is named after the mtDNA branch referred to: H1bb, H1g1, H1i1, H1q1a, H5a1d, H6a1b4, H9a, H16, H31a, I1&2, J1b1a1a, J1c2e, K1a24a, K1c1b, K2b1a1, K2a6, T2b2b1, T2b3, T2b3b, T2b5, T2b23, T2f1a1, U5a1c, U5b2b3b, U4a1b1, U4b1b1, U5a1b1a, U5b2b1a1, W1c, X2c2, H1b1g, U1a1a, U3b1c, H7a1a, H1c1, H1bw, J2b1a1, V2, H1ba, K1a3a3.

File Name: **Supplementary Data 9**

Description: **Clinically significant variants observed in Scottish and Irish Traveller participants.** Pathogenic/Likely pathogenic variants present in ClinVar with a star status of 2 or above are detailed, along with their frequencies and enrichment in Scottish or Irish Travellers.

File Name: **Supplementary Data 10**

Description: **Composition of fineSTRUCTURE clusters in figure 2a.** The numbers of samples in each of the seven fineSTRUCTURE clusters is given. N Isles, Northern Isles; Scot-TravA, Scottish Travellers cluster A; IoM, Isle of Man; Wls, Wales; Scot-TravB, Scottish Travellers cluster B; Sct-Irl, Scottish-Irish cluster; Eng-Sct, Scottish-English cluster.

File Name: **Supplementary Data 11**

Description: **Maximum parsimony tree of mitochondrial variation in the Traveller Genes samples.** Haplogroup nomenclature follows the PhyloTree database, with variants scored versus the revised Cambridge Reference Sequence. Samples are coloured according to their reported matrilineal origins.

File Name: **Supplementary Data 12**

Description: **Mitochondrial trees for 30 haplogroups reflecting inferred local British or Northwest European variation among the Traveller Genes samples.** Traveller Genes samples are included in trees along with samples from GenBank sharing diagnostic variants (Supplementary Data 8), plus unpublished mitogenomes. Each haplogroup is plotted on a separate tab, named after the group: H1bb, H1g1, H1i1, H1q1a, H5a1d, H6a1b4, H9a, H16, H31a, I1&2, J1b1a1a, J1c2e, K1a24a, K1c1b, K2b1a1, K2a6, T2b2b1, T2b3, T2b3b, T2b5, T2b23, T2f1a1, U5a1c, U5b2b3b, U4a1b1, U4b1b1, U5a1b1a, U5b2b1a1, W1c, X2c2.

File Name: **Supplementary Data 13**

Description: **Mitochondrial trees for 10 haplogroups reflecting inferred non-local variation among the Traveller Genes samples.** Traveller Genes samples are included in trees as for Supplementary Data 12. Each haplogroup is plotted on a separate tab, named after the group: H1b1g, U1a1a, U3b1c, H7a1a, H1c1, H1bw, J2b1a1, V2, H1ba, K1a3a3.
