# Supplementary material for: Genetic ancestry and monogenic disease risk in the Scottish Traveller founder population
Source: Nat Commun. 2026 Jul 15;17:5843. doi: 10.1038/s41467-026-74969-y (PMC13373179; doi:10.1038/s41467-026-74969-y)
Supplement: Supplementary file 16 — Reporting Summary [file 41467_2026_74969_MOESM16_ESM.pdf]

nature portfolio

Corresponding author(s): James F Wilson

Last updated by author(s): May 19, 2026

Reporting Summary

Nature Portfolio wishes to improve the reproducibility of the work that we publish. This form provides structure for consistency and transparency in reporting. For further information on Nature Portfolio policies, see our Editorial Policies and the Editorial Policy Checklist.

Statistics

For all statistical analyses, confirm that the following items are present in the figure legend, table legend, main text, or Methods section.

n/a	Confirmed	
☐	☒	The exact sample size (*n*) for each experimental group/condition, given as a discrete number and unit of measurement
☒	☐	A statement on whether measurements were taken from distinct samples or whether the same sample was measured repeatedly
☐	☒	The statistical test(s) used AND whether they are one- or two-sided<br>*Only common tests should be described solely by name; describe more complex techniques in the Methods section.*
☒	☐	A description of all covariates tested
☒	☐	A description of any assumptions or corrections, such as tests of normality and adjustment for multiple comparisons
☐	☒	A full description of the statistical parameters including central tendency (e.g. means) or other basic estimates (e.g. regression coefficient) AND variation (e.g. standard deviation) or associated estimates of uncertainty (e.g. confidence intervals)
☐	☒	For null hypothesis testing, the test statistic (e.g. *F*, *t*, *r*) with confidence intervals, effect sizes, degrees of freedom and *P* value noted<br>*Give P values as exact values whenever suitable.*
☒	☐	For Bayesian analysis, information on the choice of priors and Markov chain Monte Carlo settings
☒	☐	For hierarchical and complex designs, identification of the appropriate level for tests and full reporting of outcomes
☒	☐	Estimates of effect sizes (e.g. Cohen's *d*, Pearson's *r*), indicating how they were calculated

Our web collection on statistics for biologists contains articles on many of the points above.

Software and code

Policy information about availability of computer code

Data collection	No software was used for data collection.
Data analysis	All software used in this work is publicly available. It is described (with versions) throughout the manuscript and repeated here for convenience:<br>R version 4.4.2<br>R package igraph v2.1.4<br>R package leidenalg v1.1.5<br>fineSTRUCTURE v2 (includes CHROMOPAINTER)<br>ADMIXTOOLS v2<br>IBDNe v 23/04/2020<br>ASCEND v10.1.1<br>R package ggplot2 v3.5.1<br>KING v2.2<br>pbwt-paint v 14/06/2023<br>hap-ibd v 14/06/2023<br>PLINK v1.90b<br>SHAPEIT v.4<br>gnomAD ver.2.2re<br>The ClinVar-exome pipeline code is available on GitHub (https://github.com/vikinggenes/clinvar_pipeline)

For manuscripts utilizing custom algorithms or software that are central to the research but not yet described in published literature, software must be made available to editors and reviewers. We strongly encourage code deposition in a community repository (e.g. GitHub). See the Nature Portfolio guidelines for submitting code & software for further information.

Data

Policy information about availability of data

All manuscripts must include a data availability statement. This statement should provide the following information, where applicable:

- Accession codes, unique identifiers, or web links for publicly available datasets
- A description of any restrictions on data availability
- For clinical datasets or third party data, please ensure that the statement adheres to our policy

There is neither Research Ethics Committee approval, nor consent from participants, to permit open release of the individual level research data underlying this study. Instead, the research data are available through managed access, after application to each population cohort. For Traveller Genes, information is provided at https://traveller-genes.ed.ac.uk/our-research/access. All requests by researchers are reviewed by the Traveller Genes Data Access Committee and each approved project is subject to a data or materials transfer agreement, or commercial contract with the University of Edinburgh. The exome sequencing data generated in this study are also available through the same managed access process to non-commercial researchers. Information and samples from volunteers can only be used by researchers if there is relevant scientific and ethical approval for the research. We are happy to consider requests from researchers working in countries outside the UK, or in commercial companies. Personal volunteer details (such as names, email or postal address) will never be shared with researchers. Please contact TravellerGenes@ed.ac.uk for more information. The expected time-frame for responses is two weeks and data will be available for a two-year period.
Please contact Prof Gianpiero Cavalleri (gcavalleri@rcsi.ie) to access the Irish DNA Atlas genotype data(44). For the other datasets used in this study, please see the published articles for details; Trinity Student Study dataset (75), the People of the British Isles dataset (41), Avon Longitudinal Study of Pregnancy and Childhood in the Isle of Man study (74), ORCADES (71), SCOTVAR (73), Generation Scotland (70), and VIKING I (72). All other data supporting the findings of this study are available in the article or in supplementary files.

Research involving human participants, their data, or biological material

Policy information about studies with human participants or human data. See also policy information about sex, gender (identity/presentation), and sexual orientation and race, ethnicity and racism.

Reporting on sex and gender	The genetic variation reported in this manuscript is autosomal and therefore found in both sexes. There is no expectation of differences in allele frequencies between males and females; moreover, as dividing the sample in two would decrease the power of the study, no sex-based analyses were performed.
Reporting on race, ethnicity, or other socially relevant groupings	Participants in this study self reported their identity, that of their parents and their grandparents, according to a number of options:<br>Scottish Traveller<br>Highland Traveller<br>Lowland Traveller<br>Borders Romanichal Traveller<br>Showman/Fairground Traveller<br>Irish Traveller<br>English Gypsy<br>Welsh Kale<br>Settled Scottish<br>Settled Irish<br>Settled English<br>Settled Welsh<br>Mixed or Other, please specify
Population characteristics	The populations analysed were not chosen on the basis of age or any phenotypic characteristic or diagnosis. They were rather adult volunteers with parents or grandparents who identified with one of the Traveller groups listed in the previous box.<br>The mean age was 56 years and 65% were female.
Recruitment	It is difficult to predict how any self-selection may impact results, but as genotype is independent of sex, age, social class and many other common confounders, we do not foresee major biases, certainly not more than are present in all volunteer studies, such as UK Biobank or Our Future Health.<br><br>Recruitment proceeded in a very similar fashion to that described for sister study Viking Genes (described in Kerr et al. (2023) Int J Popul Data Sci. 8, 2121), except the criteria were having two or more Traveller grandparents.<br><br>To paraphrase the above citation: Traveller Genes participants were volunteers with ancestry (two or more grandparents) declared to be Scottish Travellers, Highland Travellers, Lowland Travellers, English Gypsies, Romanichal or Romany Travellers, Irish Travellers or Welsh Kale. There was no need for them to be free of disease, except if such disease would prevent them from giving consent, completing the questionnaire or physically being able to give a saliva sample. All participants were aged 16 years or more. The age of 16 is considered in UKRI Guidance to provide the right to give consent to take part in non-CTIMP research. There was no upper age limit. Males and females were eligible but, although there is no need for equal numbers of each sex, we aimed for the most balanced representation possible. The inclusion criteria were made clear to participants in several places, including the Registration of Interest Form on the study website.<br>• Participant is willing and able to give informed consent for participation in the study.<br>• Male or Female, aged 16 years or above.<br>• Has at least two Traveller grandparents.<br>Exclusion criteria were the lack of capacity to provide informed consent or the lack of Gypsy/Traveller ancestry. Those without access to the internet were also excluded, as the consent and questionnaire were completed online, so this was an

	unavoidable consequence of the recruitment method. The target was to recruit at least 150 adult volunteers with Traveller ancestry. This corresponds to approximately 5% of the total Traveller population in Scotland, not all of whom were eligible due to age or ancestry. People living outside the UK were welcomed to participate in the study. The internet and social media increasingly offer ways to improve the reach, efficiency and effectiveness of recruitment efforts, at low cost. Information about the study, including the eligibility criteria, was widely circulated in a variety of ways using a broad range of methods. This included print/online, broadcast and social media. Facebook, Twitter and Instagram were used by a variety of Traveller groups to share information about the project. This was targeted at potential participants, through posts directing them to the University of Edinburgh Traveller Genes website, after which word-of-mouth spread information further. Potential participants self-identified and as the first step was to complete a short registration of interest form on the Traveller Genes website. The Participant Information Sheet and a unique link for the Consent Form were then emailed in response. If no action was made by the participant, up to two reminder emails were sent by the Traveller Genes team. Many people came forward directly to us to enquire about participation after hearing about the study by any of the routes outlined above, and word of mouth. All participants gave Informed Consent. Participants were given the opportunity to clarify any points they did not understand in the Consent Form or Participant Information Sheet and, if necessary, ask for more information. There was no time limit for participants to consider the information provided. It was made clear that participants may withdraw their consent to participate at any time. The electronic informed consent form, signed and dated by each participant, was stored in the study database, to confirm that consent was obtained. Participants could click a link to view, save or print a copy of their consent form on the last page of the questionnaire, and the entry page of the questionnaire, if they wish to return to it for any reason after they have completed the consent form.
Ethics oversight	Participants in Traveller Genes gave written informed consent for research into health and ancestry and the study was given a favourable opinion by the NHS NRA London - London Bridge Research Ethics Committee (21/PR/1229), as well as Research and Development (3021/0222), and Caldicott Guardian (CRD21123), NHS Lothian.

Note that full information on the approval of the study protocol must also be provided in the manuscript.

Field-specific reporting

Please select the one below that is the best fit for your research. If you are not sure, read the appropriate sections before making your selection.

☒ Life sciences ☐ Behavioural & social sciences ☐ Ecological, evolutionary & environmental sciences

For a reference copy of the document with all sections, see nature.com/documents/nr-reporting-summary-flat.pdf

Life sciences study design

All studies must disclose on these points even when the disclosure is negative.

Sample size	No power calculation was performed. In population genetics the larger the sample size, the greater the ability to investigate rarer alleles, for there are more observations of each allele. The sample size here is constrained by the size of the Scottish Traveller population, but we recruited approximately 5% of the population, which is a sufficient representation.<br>Sample sizes were determined by how many individuals volunteered with the specific grandparental ancestry from these very small populations. The very fact that the Traveller population can be seen to be different on all of the different analysis proves that the sample size was sufficient to determine this. If it hadn't been, then it would not be possible to see that they were distinct.
Data exclusions	Individuals who did not have Gypsy/Traveller grandparents were excluded.
Replication	Without recruiting a new cohort of Travellers, it is not possible to replicate the findings. However, most of the population genetic analyses treat each volunteer individually, so for instance in Fig 1 the fact that the Travellers cluster together is a form of replication, as each individual could be genetically close to anyone else, yet they all appear near to each other.<br>Both Illumina chip genotyping, which most of the paper relies on, and Illumina short read sequencing, which part of it relies on, are extremely accurate technologies, deployed across thousands of peer-reviewed papers and millions of samples in industry. Unlike in clinical practice, it is not usual to verify research-standard genotypes, such as these.
Randomization	The present study does not describe a randomised controlled trial, thus allocation and randomisation are not relevant.<br>Randomisation is only relevant for where there are treatments. There are no treatments here, this is an observational study of genotypes in populations. Randomisation is irrelevant.
Blinding	The present study does not describe a blinded randomised controlled trial, thus it was not necessary to blind researchers. Subjects analysed in this study were volunteers from the population. Allocation, treatment and randomisation are not relevant to this study.<br>There are no treatments in this paper, no allocation to groups, and no randomisation; it is purely observational. There is therefore nothing to blind.

Behavioural & social sciences study design

All studies must disclose on these points even when the disclosure is negative.

Study description	*Briefly describe the study type including whether data are quantitative, qualitative, or mixed-methods (e.g. qualitative cross-sectional, quantitative experimental, mixed-methods case study).*

Research sample	*State the research sample (e.g. Harvard university undergraduates, villagers in rural India) and provide relevant demographic information (e.g. age, sex) and indicate whether the sample is representative. Provide a rationale for the study sample chosen. For studies involving existing datasets, please describe the dataset and source.*
Sampling strategy	*Describe the sampling procedure (e.g. random, snowball, stratified, convenience). Describe the statistical methods that were used to predetermine sample size OR if no sample-size calculation was performed, describe how sample sizes were chosen and provide a rationale for why these sample sizes are sufficient. For qualitative data, please indicate whether data saturation was considered, and what criteria were used to decide that no further sampling was needed.*
Data collection	*Provide details about the data collection procedure, including the instruments or devices used to record the data (e.g. pen and paper, computer, eye tracker, video or audio equipment) whether anyone was present besides the participant(s) and the researcher, and whether the researcher was blind to experimental condition and/or the study hypothesis during data collection.*
Timing	*Indicate the start and stop dates of data collection. If there is a gap between collection periods, state the dates for each sample cohort.*
Data exclusions	*If no data were excluded from the analyses, state so OR if data were excluded, provide the exact number of exclusions and the rationale behind them, indicating whether exclusion criteria were pre-established.*
Non-participation	*State how many participants dropped out/declined participation and the reason(s) given OR provide response rate OR state that no participants dropped out/declined participation.*
Randomization	*If participants were not allocated into experimental groups, state so OR describe how participants were allocated to groups, and if allocation was not random, describe how covariates were controlled.*

Ecological, evolutionary & environmental sciences study design

All studies must disclose on these points even when the disclosure is negative.

Study description	*Briefly describe the study. For quantitative data include treatment factors and interactions, design structure (e.g. factorial, nested, hierarchical), nature and number of experimental units and replicates.*
Research sample	*Describe the research sample (e.g. a group of tagged Passer domesticus, all Stenocereus thurberi within Organ Pipe Cactus National Monument), and provide a rationale for the sample choice. When relevant, describe the organism taxa, source, sex, age range and any manipulations. State what population the sample is meant to represent when applicable. For studies involving existing datasets, describe the data and its source.*
Sampling strategy	*Note the sampling procedure. Describe the statistical methods that were used to predetermine sample size OR if no sample-size calculation was performed, describe how sample sizes were chosen and provide a rationale for why these sample sizes are sufficient.*
Data collection	*Describe the data collection procedure, including who recorded the data and how.*
Timing and spatial scale	*Indicate the start and stop dates of data collection, noting the frequency and periodicity of sampling and providing a rationale for these choices. If there is a gap between collection periods, state the dates for each sample cohort. Specify the spatial scale from which the data are taken*
Data exclusions	*If no data were excluded from the analyses, state so OR if data were excluded, describe the exclusions and the rationale behind them, indicating whether exclusion criteria were pre-established.*
Reproducibility	*Describe the measures taken to verify the reproducibility of experimental findings. For each experiment, note whether any attempts to repeat the experiment failed OR state that all attempts to repeat the experiment were successful.*
Randomization	*Describe how samples/organisms/participants were allocated into groups. If allocation was not random, describe how covariates were controlled. If this is not relevant to your study, explain why.*
Blinding	*Describe the extent of blinding used during data acquisition and analysis. If blinding was not possible, describe why OR explain why blinding was not relevant to your study.*

Did the study involve field work? ☐ Yes ☐ No

Field work, collection and transport

Field conditions	*Describe the study conditions for field work, providing relevant parameters (e.g. temperature, rainfall).*
Location	*State the location of the sampling or experiment, providing relevant parameters (e.g. latitude and longitude, elevation, water depth).*
Access & import/export	*Describe the efforts you have made to access habitats and to collect and import/export your samples in a responsible manner and in*

Access & import/export	*compliance with local, national and international laws, noting any permits that were obtained (give the name of the issuing authority, the date of issue, and any identifying information).*
Disturbance	*Describe any disturbance caused by the study and how it was minimized.*

Reporting for specific materials, systems and methods

We require information from authors about some types of materials, experimental systems and methods used in many studies. Here, indicate whether each material, system or method listed is relevant to your study. If you are not sure if a list item applies to your research, read the appropriate section before selecting a response.

Materials & experimental systems

n/a	Involved in the study
☒	☐ Antibodies
☒	☐ Eukaryotic cell lines
☒	☐ Palaeontology and archaeology
☒	☐ Animals and other organisms
☒	☐ Clinical data
☒	☐ Dual use research of concern
☒	☐ Plants

Methods

n/a	Involved in the study
☒	☐ ChIP-seq
☒	☐ Flow cytometry
☒	☐ MRI-based neuroimaging

Antibodies

Antibodies used	*Describe all antibodies used in the study; as applicable, provide supplier name, catalog number, clone name, and lot number.*
Validation	*Describe the validation of each primary antibody for the species and application, noting any validation statements on the manufacturer's website, relevant citations, antibody profiles in online databases, or data provided in the manuscript.*

Eukaryotic cell lines

Policy information about cell lines and Sex and Gender in Research

Cell line source(s)	*State the source of each cell line used and the sex of all primary cell lines and cells derived from human participants or vertebrate models.*
Authentication	*Describe the authentication procedures for each cell line used OR declare that none of the cell lines used were authenticated.*
Mycoplasma contamination	*Confirm that all cell lines tested negative for mycoplasma contamination OR describe the results of the testing for mycoplasma contamination OR declare that the cell lines were not tested for mycoplasma contamination.*
Commonly misidentified lines (See ICLAC register)	*Name any commonly misidentified cell lines used in the study and provide a rationale for their use.*

Palaeontology and Archaeology

Specimen provenance	*Provide provenance information for specimens and describe permits that were obtained for the work (including the name of the issuing authority, the date of issue, and any identifying information). Permits should encompass collection and, where applicable, export.*
Specimen deposition	*Indicate where the specimens have been deposited to permit free access by other researchers.*
Dating methods	*If new dates are provided, describe how they were obtained (e.g. collection, storage, sample pretreatment and measurement), where they were obtained (i.e. lab name), the calibration program and the protocol for quality assurance OR state that no new dates are provided.*

☐ Tick this box to confirm that the raw and calibrated dates are available in the paper or in Supplementary Information.

Ethics oversight	*Identify the organization(s) that approved or provided guidance on the study protocol, OR state that no ethical approval or guidance was required and explain why not.*

Note that full information on the approval of the study protocol must also be provided in the manuscript.

Animals and other research organisms

Policy information about studies involving animals; ARRIVE guidelines recommended for reporting animal research, and Sex and Gender in Research

Laboratory animals	*For laboratory animals, report species, strain and age OR state that the study did not involve laboratory animals.*
Wild animals	*Provide details on animals observed in or captured in the field; report species and age where possible. Describe how animals were caught and transported and what happened to captive animals after the study (if killed, explain why and describe method; if released, say where and when) OR state that the study did not involve wild animals.*
Reporting on sex	*Indicate if findings apply to only one sex; describe whether sex was considered in study design, methods used for assigning sex. Provide data disaggregated for sex where this information has been collected in the source data as appropriate; provide overall numbers in this Reporting Summary. Please state if this information has not been collected. Report sex-based analyses where performed, justify reasons for lack of sex-based analysis.*
Field-collected samples	*For laboratory work with field-collected samples, describe all relevant parameters such as housing, maintenance, temperature, photoperiod and end-of-experiment protocol OR state that the study did not involve samples collected from the field.*
Ethics oversight	*Identify the organization(s) that approved or provided guidance on the study protocol, OR state that no ethical approval or guidance was required and explain why not.*

Note that full information on the approval of the study protocol must also be provided in the manuscript.

Clinical data

Policy information about clinical studies

All manuscripts should comply with the ICMJE guidelines for publication of clinical research and a completed CONSORT checklist must be included with all submissions.

Clinical trial registration	*Provide the trial registration number from ClinicalTrials.gov or an equivalent agency.*
Study protocol	*Note where the full trial protocol can be accessed OR if not available, explain why.*
Data collection	*Describe the settings and locales of data collection, noting the time periods of recruitment and data collection.*
Outcomes	*Describe how you pre-defined primary and secondary outcome measures and how you assessed these measures.*

Dual use research of concern

Policy information about dual use research of concern

Hazards

Could the accidental, deliberate or reckless misuse of agents or technologies generated in the work, or the application of information presented in the manuscript, pose a threat to:

No	Yes	
☐	☐	Public health
☐	☐	National security
☐	☐	Crops and/or livestock
☐	☐	Ecosystems
☐	☐	Any other significant area

Experiments of concern

Does the work involve any of these experiments of concern:

No	Yes	
☐	☐	Demonstrate how to render a vaccine ineffective
☐	☐	Confer resistance to therapeutically useful antibiotics or antiviral agents
☐	☐	Enhance the virulence of a pathogen or render a nonpathogen virulent
☐	☐	Increase transmissibility of a pathogen
☐	☐	Alter the host range of a pathogen
☐	☐	Enable evasion of diagnostic/detection modalities
☐	☐	Enable the weaponization of a biological agent or toxin
☐	☐	Any other potentially harmful combination of experiments and agents

Plants

Seed stocks	n/a
Novel plant genotypes	n/a
Authentication	n/a

ChIP-seq

Data deposition

☐ Confirm that both raw and final processed data have been deposited in a public database such as GEO.

☐ Confirm that you have deposited or provided access to graph files (e.g. BED files) for the called peaks.

Data access links<br>*May remain private before publication.*	*For "Initial submission" or "Revised version" documents, provide reviewer access links. For your "Final submission" document, provide a link to the deposited data.*
Files in database submission	*Provide a list of all files available in the database submission.*
Genome browser session<br>(e.g. UCSC)	*Provide a link to an anonymized genome browser session for "Initial submission" and "Revised version" documents only, to enable peer review. Write "no longer applicable" for "Final submission" documents.*

Methodology

Replicates	*Describe the experimental replicates, specifying number, type and replicate agreement.*
Sequencing depth	*Describe the sequencing depth for each experiment, providing the total number of reads, uniquely mapped reads, length of reads and whether they were paired- or single-end.*
Antibodies	*Describe the antibodies used for the ChIP-seq experiments; as applicable, provide supplier name, catalog number, clone name, and lot number.*
Peak calling parameters	*Specify the command line program and parameters used for read mapping and peak calling, including the ChIP, control and index files used.*
Data quality	*Describe the methods used to ensure data quality in full detail, including how many peaks are at FDR 5% and above 5-fold enrichment.*
Software	*Describe the software used to collect and analyze the ChIP-seq data. For custom code that has been deposited into a community repository, provide accession details.*

Flow Cytometry

Plots

Confirm that:

- [ ] The axis labels state the marker and fluorochrome used (e.g. CD4-FITC).
- [ ] The axis scales are clearly visible. Include numbers along axes only for bottom left plot of group (a 'group' is an analysis of identical markers).
- [ ] All plots are contour plots with outliers or pseudocolor plots.
- [ ] A numerical value for number of cells or percentage (with statistics) is provided.

Methodology

Sample preparation	*Describe the sample preparation, detailing the biological source of the cells and any tissue processing steps used.*
Instrument	*Identify the instrument used for data collection, specifying make and model number.*
Software	*Describe the software used to collect and analyze the flow cytometry data. For custom code that has been deposited into a community repository, provide accession details.*
Cell population abundance	*Describe the abundance of the relevant cell populations within post-sort fractions, providing details on the purity of the samples and how it was determined.*
Gating strategy	*Describe the gating strategy used for all relevant experiments, specifying the preliminary FSC/SSC gates of the starting cell population, indicating where boundaries between "positive" and "negative" staining cell populations are defined.*

- [ ] Tick this box to confirm that a figure exemplifying the gating strategy is provided in the Supplementary Information.

Magnetic resonance imaging

Experimental design

Design type	*Indicate task or resting state; event-related or block design.*
Design specifications	*Specify the number of blocks, trials or experimental units per session and/or subject, and specify the length of each trial or block (if trials are blocked) and interval between trials.*
Behavioral performance measures	*State number and/or type of variables recorded (e.g. correct button press, response time) and what statistics were used to establish that the subjects were performing the task as expected (e.g. mean, range, and/or standard deviation across subjects).*

Acquisition

Imaging type(s)	*Specify: functional, structural, diffusion, perfusion.*
Field strength	*Specify in Tesla*
Sequence & imaging parameters	*Specify the pulse sequence type (gradient echo, spin echo, etc.), imaging type (EPI, spiral, etc.), field of view, matrix size, slice thickness, orientation and TE/TR/flip angle.*
Area of acquisition	*State whether a whole brain scan was used OR define the area of acquisition, describing how the region was determined.*

Diffusion MRI ☐ Used ☐ Not used

Preprocessing

Preprocessing software	*Provide detail on software version and revision number and on specific parameters (model/functions, brain extraction, segmentation, smoothing kernel size, etc.).*
Normalization	*If data were normalized/standardized, describe the approach(es): specify linear or non-linear and define image types used for transformation OR indicate that data were not normalized and explain rationale for lack of normalization.*
Normalization template	*Describe the template used for normalization/transformation, specifying subject space or group standardized space (e.g. original Talairach, MNI305, ICBM152) OR indicate that the data were not normalized.*
Noise and artifact removal	*Describe your procedure(s) for artifact and structured noise removal, specifying motion parameters, tissue signals and physiological signals (heart rate, respiration).*

Volume censoring | *Define your software and/or method and criteria for volume censoring, and state the extent of such censoring.*

Statistical modeling & inference

Model type and settings | *Specify type (mass univariate, multivariate, RSA, predictive, etc.) and describe essential details of the model at the first and second levels (e.g. fixed, random or mixed effects; drift or auto-correlation).*

Effect(s) tested | *Define precise effect in terms of the task or stimulus conditions instead of psychological concepts and indicate whether ANOVA or factorial designs were used.*

Specify type of analysis: ☐ Whole brain ☐ ROI-based ☐ Both

Statistic type for inference | *Specify voxel-wise or cluster-wise and report all relevant parameters for cluster-wise methods.*

(See Eklund et al. 2016)

Correction | *Describe the type of correction and how it is obtained for multiple comparisons (e.g. FWE, FDR, permutation or Monte Carlo).*

Models & analysis

n/a	Involved in the study
☐	☐ Functional and/or effective connectivity
☐	☐ Graph analysis
☐	☐ Multivariate modeling or predictive analysis

Functional and/or effective connectivity | *Report the measures of dependence used and the model details (e.g. Pearson correlation, partial correlation, mutual information).*

Graph analysis | *Report the dependent variable and connectivity measure, specifying weighted graph or binarized graph, subject- or group-level, and the global and/or node summaries used (e.g. clustering coefficient, efficiency, etc.).*

Multivariate modeling and predictive analysis | *Specify independent variables, features extraction and dimension reduction, model, training and evaluation metrics.*
