# Supplementary material for: Genetic ancestry and monogenic disease risk in the Scottish Traveller founder population
Source: Nat Commun. 2026 Jul 15;17:5843. doi: 10.1038/s41467-026-74969-y (PMC13373179; doi:10.1038/s41467-026-74969-y)
Supplement: Supplementary file 17 — Transparent Peer Review file [file 41467_2026_74969_MOESM17_ESM.pdf]

nature portfolio

Peer Review File

Genetic ancestry and monogenic disease risk in the Scottish Traveller founder population

Corresponding Author: Professor James Wilson

This file contains all reviewer reports in order by version, followed by all author rebuttals in order by version.

Version 1:

Reviewer comments:

Reviewer #1

(Remarks to the Author)
This is a good paper and worthy of publication. A couple of points- line 55- is it not better to say Mainland Europe? Continental is not used commonly in scientific literature? I think the Introduction is too long. From line 59 to 92, this is meant to be a genetic paper (I think) rather than a sociological/anthropological review? I would condense these lines. Line 94 on is relevant. I like the surname and geneology piece. I note you mention 2 had ROma origin (& one other had Gypsy origin ? unknown) and you had checked for common Irish Trav known variants- there is a recent publication S Quinn et all EJ Med Genet PMID 39709002 where >100 (mainland!) European Roma variants are characterised and were checked if present in an Indian exome cohort. Did these 2/3 people of Gypsy origin have any of the known Roma variants? When the discussion focusses on homozygosity, in Lynch 2018 paper on Irish Travellers there is a graph [figure 1] illustrating levels of homozygosity in comparison to other populations. It would be nice if the authors could do something similar as its a nice visual image- perhaps comparing the Pakistani paper reports they have already cited, ref 33 they have already cited, Roma data they have already cited and comparing Scottish Travellers degree of homozygosity in comparison to Scottish population and English if they want- in a graph?
line 697 I am not sure the term full-bred is a recognised one? I am not sure what is meant by this??

Reviewer #2

(Remarks to the Author)
The manuscript “Genetic ancestry and monogenic disease risk in the Scottish Traveller founder population," undertakes a genomic analysis of the Traveller population to elucidate its history. Investigating founder populations, especially those under-represented in genetic research, is a valuable endeavor, and it is remarkable the efforts performed by the authors in characterizing the Scottish Traveller population.
While the manuscript presents a series of genetic analyses, several key areas require substantial revision to meet the scientific rigor and scope expected for publication in a journal like Nature Communications. My main feedback focuses on the need to move beyond a purely descriptive account to a more hypothesis-driven investigation that delivers deeper biological and clinical insights.
My main concern is the lack of a central hypothesis and biological question. The manuscript currently reads as a collection of genetic analyses performed on a specific population, rather than a focused study driven by a clear scientific question or hypothesis. While characterizing the population is a valid goal, Nature Communications typically seeks studies that advance fundamental biological understanding or provide significant insights into disease mechanisms or population genetics. The current structure makes it difficult to discern the core message or the primary scientific contribution. For instance, the analysis of surnames, while valid, could be presented as supplementary information, allowing the main text to focus on the population genetic inferences derived from this analysis.
Another concern is the limited and vague sample description. The sample size in some of analyses (especially those involving the Irish Traveller sample and clinical variants) appears relatively small for comprehensive population genetic studies, especially when attempting to infer detailed demographic histories or robustly estimate allele frequencies for rare variants. More importantly, the description of these samples is vague, making it difficult to assess the representativeness or potential biases. It is unclear how robust the results are when some analyses (PCA, fineStructure, clustering analysis, estimation of clinical relevant variants…) include volunteers whose grandparents do not belong to the community. While this inclusion increases the sample size, this issue should be explicitly addressed and corrected to avoid potential artifacts.
Regarding more specific points, the conclusions drawn from the PCA results are overstated. PCA plots are visualizations of

the data, and it is risky to draw definitive conclusions without formal statistical tests. The PCA could also be performed excluding samples from Orkney and Shetland Islands, as these groups appear to be clear outliers. This might help to clarify the relationships among the remaining samples.
More specific details should be provided for the FineStructure analysis. I was unable to find the number of individuals and composition of each genetic cluster represented in Figure 2. Defining the clusters with a highly diverse or very limited number of individuals could bias the results and conclusion of the analyses.
In conclusion, while the manuscript addresses an important and under-researched area, significant revisions are necessary to elevate it to the standard required for Nature Communications. Focusing on a clear biological question, providing deeper insights beyond mere description, and strengthening the sample characterization and comparative analyses will be crucial.

Reviewer #3

(Remarks to the Author)
In this paper Shanmugam and colleagues generate new genome-wide and exome sequencing data from individuals who self-identify as Scottish travellers. This is a historically marginalized and highly understudied group that has far as I understand have never been characterized genomically prior to this work. The authors first perform some analysis using the accompanied demographic data assembled during the DNA collection process of 125 individuals, and use this to examine the extent of enrichment of certain surnames within specific areas of Scotland. Then using a subset of samples (n=48) they generate genome-wide SNP data, and use this to examine the genetic structure and demographic history of the population. They do this using a suite of methods appropriate for genome-wide SNP data such as PCA, chromosome painting, IBD-based analysis, and ancestry clustering. They contexualize the Scottish traveller samples using reference data sets from "settled" British Isle's individuals and other traveller individuals. They also parse the Scottish Traveller population by self-reported ethnicity, such as the number of grandparents being from a particular traveller population. The noteworthy take away from this analysis is that Scottish Traveller populations largely cluster with settled Scottish individuals, while exhibiting some internal population structure and endogamy as measured by ROH and IBD-based analysis. They show no real relationship to Roma gypsy individuals from Europe. The Scottish traveller individuals with substantial self reported grandparental ancestry also form two clusters, with cluster A showing particularly small recent Ne and high levels of ROH.

The authors then generate exome sequencing data for all 125 samples to examine the extent that Scottish travellers might possess an enhanced risk for inherited disease due to enrichment of pathogenic SNP variants due to the increased rate of endogamy and drift. They find some evidence of this for a handful of variants related to diseases, though all in recessive form. They also make use of the fact that they also have a small number of Irish traveller genomes imbedded in their collection, and look for any overlap between variants common between them.

In general this paper is well written. In general I enjoyed reading it and found it interesting. The data generation is well decribed and robustly analyzed using the appropriate population genetic methods. I have no real issues from a technical level in terms of how the data have been analyszed. By modern standards, generation of genome-wide SNP data for 50 individuals and exome data for 125 is not particulaly cutting-edge. However, it must also be taken into account that obtaining the samples themselves is highly challenging, and the authors appear to have gone through a very thoughful process to perform thus study, likely requiring significiant effort. While I would love to have seen high coverage whole genome data for these samples to bring it up to date with the current state-of-the-art and allow the author's to use more powerful ARG-based analyses for the popgen and explore things like polygenic non-exomic variants, I think the fact that this is the first Scottish traveller data of any kind makes the dataset valuable regardless.

The analysis of population genetic structure and demographic history is robust. I do not have many suggested improvements of this section. I think showing the close link to the settled Scottish population is important, given their marginalized status, and may even have important implications socially. From a techinical perspective, I would like to see a visualization of the IBD results. The analysis of its inference is described, but then it is just treated as raw material for methods like IBD-Ne and the clustering analysis. I would be interested in seeing for example what an IBD network looks like amongst the population and if it visualizes the two clusters somehow.

I found the pathogenic variant section less compelling. It finds some risk variants and is largely descriptive, but there is no real take away other than "this is important to know". No link is really made to what is found and what is known about about specific diseases/disorders Scottish traveller's tend to exhibit most. Do the findings appear to fit the clinical history of the population? Also, despite the high levels of homozygosity in the population, the pathogenic variants are usually found in singleton form, making the recessive variants largely harmless. Is this the result of the sample collection, for example only picking healthy individuals somehow? Or is there a broader evolutionary argument being made here about the purging of very deleterious dominant mutations? Given the large exome dataset, it would probably be worth examining how the allele frequency spectrum of the population looks at synonymous vs nonsynomymoys sites, and comparing the distribution of fitness effects to the "settled" Scottish individuals? Is there any evidence of a skew? This would be much more useful I think than the large section on mtDNA haplotypes, which could be shortened as it does not add much. Also beyond the "this is important to know", does the observed distribution of pathogenic variants mean some specific health policy intervention should be explored. There is a brief mention of the similarity to Jewish recessive screening, so a bit more detail here would be useful.

Specific comments
Line 151: "All Traveller Genes participants with DNA passing QC were exome sequenced, and 48 unrelated participants

with maximal Traveller ancestry had additional genome-wide SNP array genotypes generated, for joint analysis with reference populations."
Does "maximal Traveller ancestry" mean four grand parents or is it determined by genetic ancestry?

Line 551: "This is the first genetic study of the Scottish Travellers and should therefore help to clarify the debate around their origins"
I am not really sure the study tells us about the origins of Scottish Travellers. It tells us about the genetic structure, which is interesting, but is very different from "origins". At most the author's have ruled out a Roma connection. But the analysis has not revealed things like the time or place of their origins (which is probably multifactorial). I think the discussion should reflect that

Version 2:

Reviewer comments:

Reviewer #1

(Remarks to the Author)
I think this paper is good and contains some important work. The data is important to publish. With regard to the audience, I still think it might be better to split into two papers, one sociology of the Scottish Travellers and then have a more concise condensed one discussing the results of the array and sequencing?

However, I understand where the authors are coming from. If the journal can afford the space the sociological element takes up then it would be fine but if space is tight I think the authors could still consider getting two papers out of this?

Reviewer #2

(Remarks to the Author)
The authors have made a remarkable effort to refine the manuscript; the updated presentation of the hypotheses and results represents a substantial improvement.
However, I would appreciate further clarification regarding the proposed substructure of Scottish Travellers (Groups A and B), as one of the conclusions appears slightly paradoxical. The manuscript suggests that Group B is closer to the ancestral population, while Group A is a drifted descendant. Yet, the authors also correctly note that Group B is an admixed group that has experienced recent gene flow. Given this admixture, is it possible that Group A actually represents the more 'ancestral' lineage, one that appears more distant simply because it has remained isolated and undergone significant genetic drift, rather than having its distance “reduced” by gene flow as seen in Group B? I would welcome the authors' comments on this alternative interpretation.

Reviewer #3

(Remarks to the Author)
In general the author's have addressed my comments. However, their reply to my comment regarding the allele frequency spectrum is not really sufficient:
"we don’t believe we would have power to detect differences in synonymous: non-synonymous ratios." What is this argument based on?

The authors are claiming that rare deleterious alleles are enriched in the traveller population because of their lower Ne. They are essentially making an argument for higher genetic load when comparing to the non-Finnish Europeans in gnomAD. The relative AFS for non-synonymous versus synonymous sites compared to a control population from 125 individuals with whole exome data is likely to result in some skew in the rare variant category, for example simply comparing to 1000G Europeans. In addition, the authors could also look at the distribution of pathogenic variants within and outside ROH. I would at least like to see these analyses attempted before simply dismissing them, as positive results would very much strengthen the case of increased attention on disease burden.

Version 3:

Reviewer comments:

Reviewer #2

(Remarks to the Author)

Reviewer #3

(Remarks to the Author)
I guess then there's nothing that can be done.

Open Access This Peer Review File is licensed under a Creative Commons Attribution 4.0 International License, which permits use, sharing, adaptation, distribution and reproduction in any medium or format, as long as you give appropriate credit to the original author(s) and the source, provide a link to the Creative Commons license, and indicate if changes were made.
In cases where reviewers are anonymous, credit should be given to 'Anonymous Referee' and the source.
The images or other third party material in this Peer Review File are included in the article’s Creative Commons license, unless indicated otherwise in a credit line to the material. If material is not included in the article’s Creative Commons license and your intended use is not permitted by statutory regulation or exceeds the permitted use, you will need to obtain permission directly from the copyright holder.
To view a copy of this license, visit https://creativecommons.org/licenses/by/4.0/

Reviewer #1

This is a good paper and worthy of publication. A couple of points- line 55- is it not better to say Mainland Europe? Continental is not used commonly in scientific literature?

We thank the reviewer for their positive feedback. We have changed the phrase to Mainland Europe.

I think the Introduction is too long. From line 59 to 92, this is meant to be a genetic paper (I think) rather than a sociological/anthropological review? I would condense these lines. Line 94 on is relevant.

We thank the reviewer for their observation and have shortened the section slightly, as requested. We feel, however, that the introduction provides important context to a poorly known community whose genetic history we have described for the first time. We were approached by the Scottish Travellers, who would like their history to be described impartially, given the limited historical documentation and marginalisation of the community. This includes lines 60-62 describing the *languages* used by the community, lines 59 & 63-65 explaining their *culture*, and lines 65-67 defining how Scottish Travellers *self-identify*; all relevant to a paper describing the population for the first time. Lines 71-76 summarise the sparse documentation in historical texts, also clearly of relevance to a manuscript describing the genetic history of this community for the first time. Lines 77-82 outline various hypotheses for the origins of the Scottish Travellers, which we feel is important, particularly given that reviewer two requested further clarity on the hypotheses being tested. Finally, lines 83-92 describe the marginalisation of the Scottish Travellers, which the community did not want glossed over.

I like the surname and geneology piece. I note you mention 2 had Roma origin (& one other had Gypsy origin ? unknown) and you had checked for common Irish Trav known variants- there is a recent publication S Quinn et all EJ Med Genet PMID 39709002 where >100 (mainland!) European Roma variants are characterised and were checked if present in an Indian exome cohort. Did these 2/3 people of Gypsy origin have any of the known Roma variants?

We thank the reviewer for the positive words and also for pointing us to the paper. Just three of the 111 variants identified in Quinn *et al.* are present in the individuals from our dataset. From look-ups in UK Biobank European-heritage individuals (from the UKB Allele Frequency Browser), they are all low frequency variants which are reasonably "common" in the European pathogenic pool: *ACADM* c.985A>G, n = 1 in the Travellers, n=7607 in UK-EUR; *CFTR* c.1521_1523delCTT, n = 5 in the Travellers, n=15,009 in UKB-EUR; *GJB2* c.35delG, n = 1 in the Travellers, n=6894 in UKB-EUR. We did not find any of the variants from Quinn et al. in participants reporting Roma ancestry.

When the discussion focusses on homozygosity, in Lynch 2018 paper on Irish Travellers there is a graph [figure 1] illustrating levels of homozygosity in comparison to other populations. It would be nice if the authors could do something similar as its a nice visual image- perhaps comparing the Pakistani paper reports they have already cited, ref 33 they have already cited, Roma data they have already cited and comparing Scottish Travellers degree of homozygosity in comparison to Scottish population and English if they want- in a graph?

We thank the reviewer for this suggestion and have now added a bar plot (the current Fig. 4a; line 330 – see below) comparing the levels of homozygosity between reference samples from the 1000 Genomes Project, the Irish Travellers and the Scottish Travellers, as well as settled Scottish and settled Irish. The figure shows that the Scottish Travellers have high levels of homozygosity, similar to the Punjabis in Lahore, Pakistan and Sri Lankan Tamils in the UK. We have also added in a new supplementary figure (4), showing a similar global comparison, but for shorter ROH.

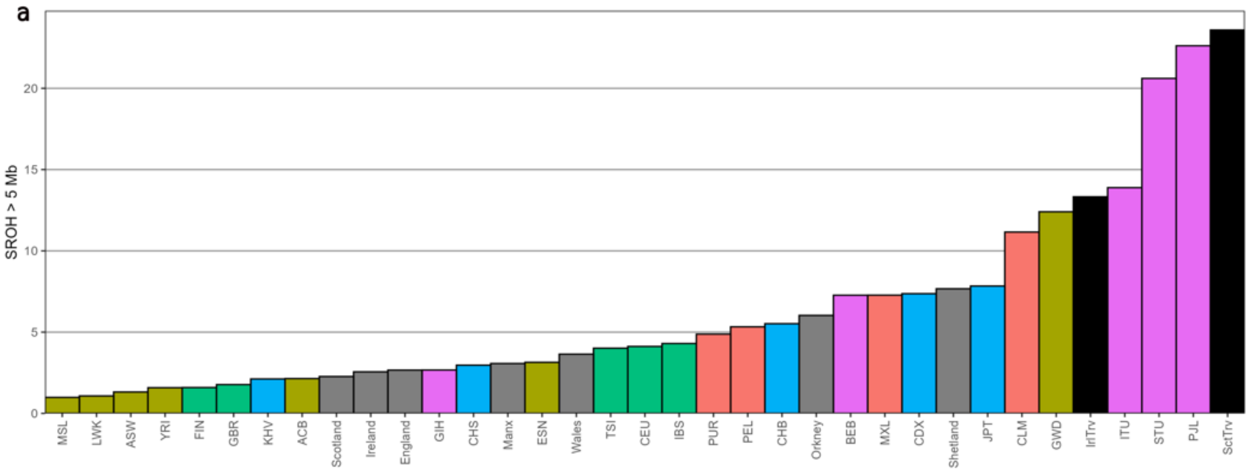

line 697 I am not sure the term full-bred is a recognised one? I am not sure what is meant by this??

Thank you for pointing that out. We have now referred to them as “Scottish Travellers with four Traveller grandparents” (line 735). We have also removed “full-blooded” at line 202.

Reviewer #2

The manuscript “Genetic ancestry and monogenic disease risk in the Scottish Traveller founder population," undertakes a genomic analysis of the Traveller population to elucidate its history. Investigating founder populations, especially those under-represented in genetic research, is a valuable endeavor, and it is remarkable the efforts performed by the authors in characterizing the Scottish Traveller population.

We thank the reviewer for their extremely positive summary of our work.

While the manuscript presents a series of genetic analyses, several key areas require substantial revision to meet the scientific rigor and scope expected for publication in a journal like Nature Communications. My main feedback focuses on the need to move beyond a purely descriptive account to a more hypothesis-driven investigation that delivers deeper biological and clinical insights.
My main concern is the lack of a central hypothesis and biological question. The manuscript currently reads as a collection of genetic analyses performed on a specific population, rather than a focused study driven by a clear scientific question or hypothesis.

The research questions that drove our analysis were developed in partnership with members of the Scottish Traveller community. These are simple, but important questions the community were keen to understand the answers to. We felt we had phrased these hypotheses clearly in the introduction of our original submission, particularly in lines 122-127. But we appreciate the sentiment of this comment, and to make these clearer, we have now revised this section to read: “…we therefore sought to (a) infer whether the Scottish Traveller community is genetically distinct; (b) clarify the genetic origins of the

Scottish Travellers and the extent of genetic relatedness to the “settled” Scottish populations and other Gypsy/Traveller communities; (c) test whether there is any genetic structure within the Scottish Traveller community; and (d) identify known pathogenic variants causing monogenic diseases, including any founder effects, to better understand community-specific health risks.”

The essence of this is a hypothesis test of whether the Scottish Travellers were in fact genetically distinct or were not differentiated from other British populations. As they were distinct, further hypotheses were then tested as to whether they were related to Roma populations or to other Scottish populations. These hypotheses are outlined in the introduction (lines 77-81) and then each is tested in turn, before going on to test whether there is population genetic structure within the Travellers, and whether there have been founder effects on pathogenic variants in the Traveller population, which translate into clinically important risks.

While characterizing the population is a valid goal, Nature Communications typically seeks studies that advance fundamental biological understanding or provide significant insights into disease mechanisms or population genetics.

We feel the manuscript provides substantial insights into the population genetics of an isolated and vulnerable community, in a manner that can have significant positive impact for that community. Moreover, the work was developed and delivered in partnership with the community, providing an important example of the benefits that community-driven research can bring. We also note that a very similar 2019 *Nature Communications* paper which explores the population structure of Spain (https://www.nature.com/articles/s41467-018-08272-w) features in the list of the top 50 biological papers published in the journal that year. Furthermore, comparable approaches are featured regularly in *Nature Communications* papers (e.g., https://www.nature.com/articles/s41467-020-18418-4), as well as in *Science Advances* (e.g., https://www.science.org/doi/10.1126/sciadv.abq2616).

The current structure makes it difficult to discern the core message or the primary scientific contribution.

The core messages of our paper are laid out in the abstract, among other places in the text. For example, quoting directly from the abstract here:

- **“We demonstrate that Scottish Travellers are genetically distinct from Irish Travellers, English Gypsies and European Roma, as well as the settled British and Irish populations.”**
- **“However, they do share autosomal and mitochondrial genetic ancestry with settled Scots.”**
- **“Two genetic subgroups are detectable: one which is more drifted and one more admixed.”**
- **“High levels of autozygosity are apparent, consistent with consanguinity.”**
- **“We detect signals of bottlenecks in autosomal and mitochondrial data.”**
- **“Importantly, we identified an enrichment of rare, pathogenic variants, including at least five putative founder variants associated with recessive Mendelian disorders.”**

For instance, the analysis of surnames, while valid, could be presented as supplementary information, allowing the main text to focus on the population genetic inferences derived from this analysis.

We note that reviewer one complimented our surname and genealogy analysis and we believe that it adds to the understanding of a population that has never been described in the scientific literature before. For instance, population genetics alone cannot inform us that seven surnames account for 58% of the Scottish Traveller grandparents, a rather remarkable statistic, of interest to population geneticists, historians and the public at large, who might be reading this multidisciplinary journal.

Another concern is the limited and vague sample description. The sample size in some of analyses (especially those involving the Irish Traveller sample and clinical variants) appears relatively small for comprehensive population genetic studies, especially when attempting to infer detailed demographic histories or robustly estimate allele frequencies for rare variants. More importantly, the description of these samples is vague, making it difficult to assess the representativeness or potential biases. It is unclear how robust the results are when some analyses (PCA, fineStructure, clustering analysis, estimation of clinical relevant variants…) include volunteers whose grandparents do not belong to the community. While this inclusion

increases the sample size, this issue should be explicitly addressed and corrected to avoid potential artifacts.

We have sampled about 3% of the Traveller community in Scotland, which was no small feat; for instance, it represents a much greater proportion of the Scottish Traveller census population than UK Biobank represents of the UK population. We accept that the Irish Traveller sample is very small, but we were not attempting detailed demographic historic inference or allele frequency estimates in that population. We were only seeking to investigate whether the Scottish Travellers and the Irish Travellers were genetically distinct or alike, which requires fewer samples.

We agree with the reviewer that it is of great importance to be careful which samples from the real-world population who volunteer for a study like this are included in each analysis. We therefore recorded detailed information from each volunteer and this is described in considerable detail in the results, for instance from lines 139-145, explaining their numbers, different community backgrounds and number of Traveller grandparents, also summarised in Table 1 and detailed in Supplementary Table 1. We even include a section on their surnames and genealogies, commended by reviewer one. We go on to describe from lines 146-149: "Participants with all 4 Scottish Traveller grandparents were labelled "SctTrv", those with all Irish Traveller grandparents were labelled "IrlTrv", those with English Gypsy grandparents as "GypTrv", and finally those with Traveller and also "settled" (non-Traveller) population ancestry as "Mixed"."

Thus, throughout these analyses (e.g., Figs. 1-2) a clear distinction is made, labeling individuals *without* all 4 grandparents from the community as Mixed vs those with all 4 grandparents as SctTrv. In the analyses underlying Fig. 3, we only included individuals with 4 Traveller grandparents. To make this clearer, we have now added the following sentence to the Fig. 3 legend (lines 319-230): "Only individuals with all four grandparents declared to be Travellers were included in this analysis."

For the genomic ROH analysis (Fig 4), models were explicitly run comparing Scottish Travellers with 2, 3 and 4 grandparents, again showing our results are not biased by these inclusions, but instead enriched. The inferences are thus robust to any inclusion of volunteers with non-Traveller grandparents.

Finally, we explicitly modelled partial Traveller ancestry when estimating the allele frequencies of clinically important variants. We have also now added a sentence to the Table 2 legend (lines 498-499) to explain that: "as described in Methods, the frequency uplift estimates took into account the number of grandparents who were Travellers." Seventeen of the 22 variants featured in Table 2 have been observed at least once in participants with four Traveller grandparents and we have indicated in the legend (lines 496-497) those which were only seen in participants with two or three Traveller grandparents. We have also now added a column to Supplementary Table 9 indicating for every variant, whether it was observed in participants with four Traveller grandparents.

Regarding more specific points, the conclusions drawn from the PCA results are overstated. PCA plots are visualizations of the data, and it is risky to draw definitive conclusions without formal statistical tests. The PCA could also be performed excluding samples from Orkney and Shetland Islands, as these groups appear to be clear outliers. This might help to clarify the relationships among the remaining samples.

We thank the reviewer for suggesting rerunning the PCA without Orkney and Shetland, which are the most differentiated populations in the British Isles. We have done this and we also removed the Manx, who are likewise islanders. As can be seen below, the simpler PCs continue to show the same pattern, namely that the Scottish Travellers group in genetic space with other Scottish samples in the early principal components (e.g., PC1 vs PC2 or PC3) and then separate out in later components – this time very clearly at PC8, but also partially on PC1 and PC3. We have now included these analyses as Supplementary Figures 8 and 9 and referenced them in the text (lines 230-232). The new analyses accord with the t-SNE dimension reduction analysis (Fig 1c), which shows the Scottish Travellers, the Orcadians, the Shetlanders, and the Manx as separate clusters from one another and from the Mainland British and Irish.

Supplementary Fig. 8 Principal components analysis without islander individuals. *(a) The first and second principal components of a pbwt-paint chunk counts matrix between British and Irish ancestry references and Traveller individuals. Individual points are colour-coded by the genetic clusters that they were assigned to. Black points represent Traveller samples grouped by fineSTRUCTURE cluster, with remaining individuals colour-coded according to their IBD-*

network cluster membership. The PCA was carried out in R, and the graph plotted using the ggplot2 R package.; (b) Detail of Anglo-Irish ancestry cline, showing overlap of Scottish Travellers with the Scottish part of the cline.

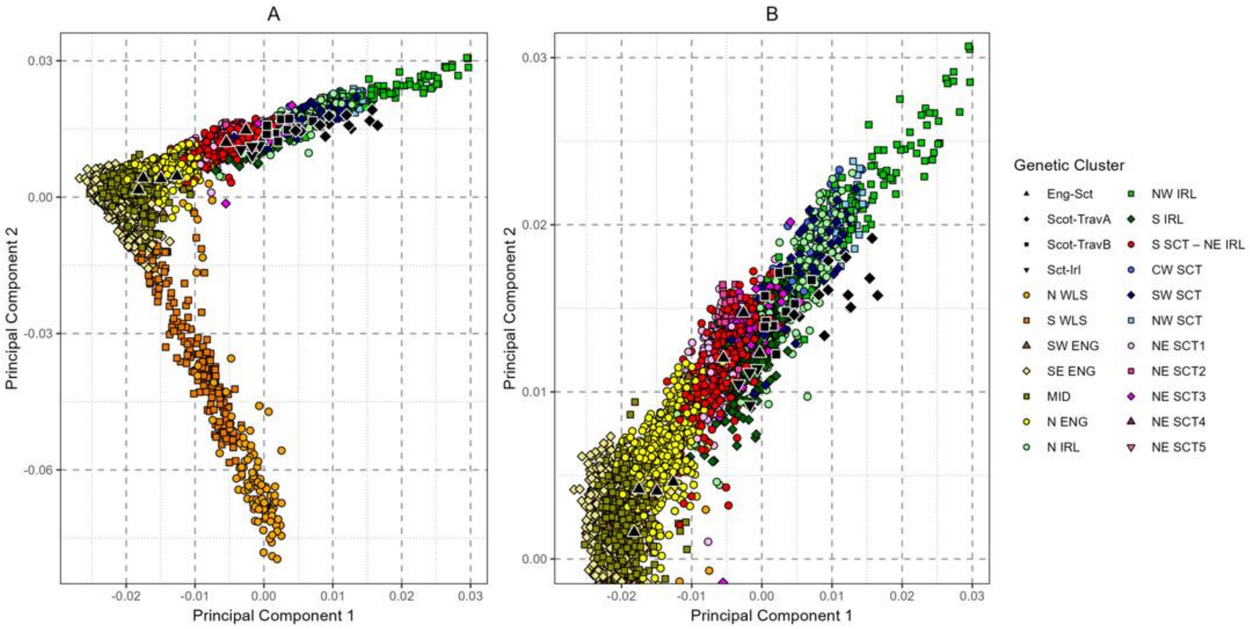

Supplementary Fig. 9 Principal components analysis without islander individuals. *(a) The first and third principal components of a pbwt-paint chunk counts matrix between British and Irish ancestry references and Traveller individuals. Individual points are colour-coded by the genetic clusters that they were assigned to. Black points represent Traveller samples grouped by fineSTRUCTURE cluster, with remaining individuals colour-coded according to their IBD-network cluster membership. The PCA was carried out in R, and the graph plotted using the ggplot2 R package.; (b) The first and eighth principal components.*

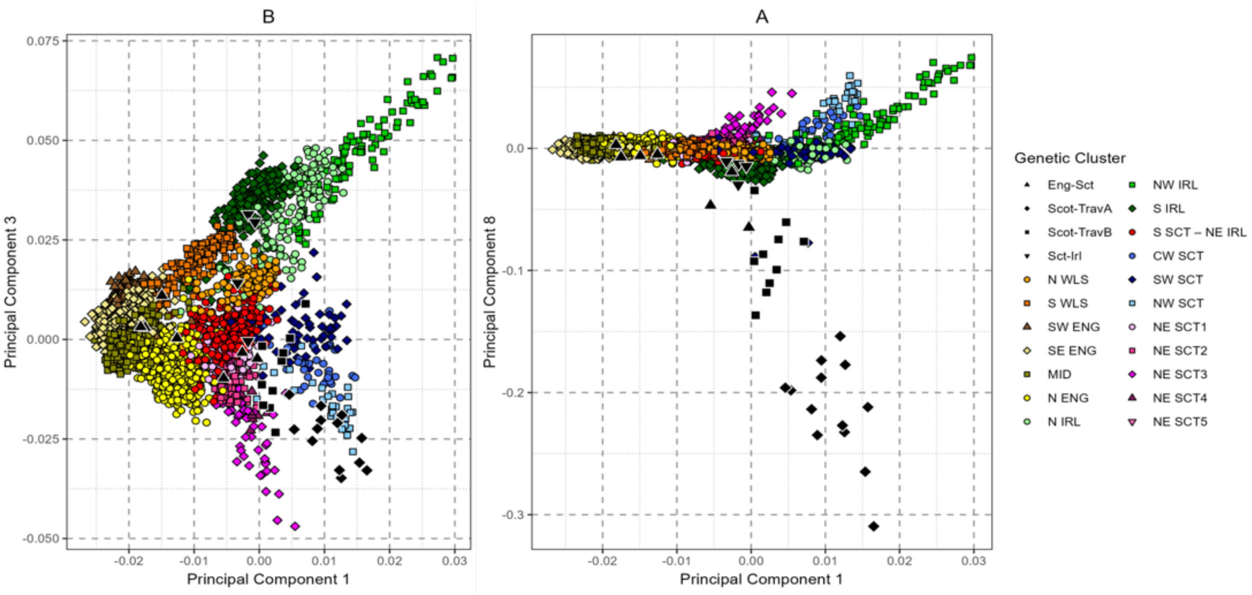

We have also pasted in Fig. 1 for comparison:

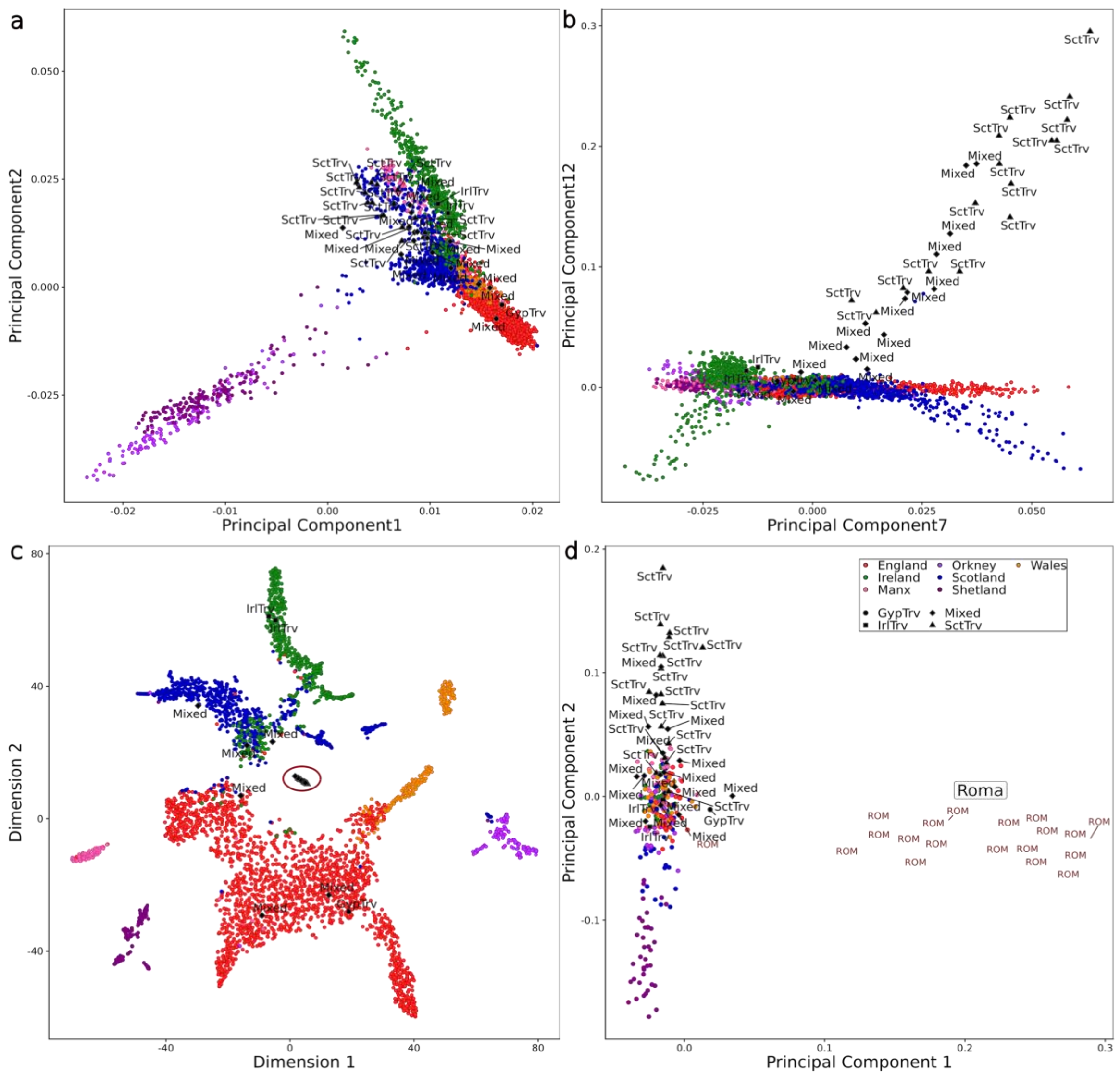

More specific details should be provided for the FineStructure analysis. I was unable to find the number of individuals and composition of each genetic cluster represented in Figure 2. Defining the clusters with a highly diverse or very limited number of individuals could bias the results and conclusion of the analyses.

We thank the reviewer for the comment. We have now included Supplementary Table 10 showing the number of individuals and composition of the fineSTRUCTURE clusters shown in Fig. 2. We have also included further text in the methods to clarify the samples included in this analysis (lines 857-859).

In conclusion, while the manuscript addresses an important and under-researched area, significant revisions are necessary to elevate it to the standard required for Nature Communications. Focusing on a clear biological question, providing deeper insights beyond mere description, and strengthening the sample characterization and comparative analyses will be crucial.

We thank the reviewer for noting that we have addressed an important and under-researched area. We are also grateful for the constructive suggestions that have helped improve the clarity of several parts of the manuscript. As outlined in our responses above and in the manuscript, the main hypothesis being tested here was whether the Scottish Travellers were genetically distinct from the Scots, who they were most closely related to and whether they were at increased risk of any Mendelian diseases because of founder effects. We have used a highly overlapping suite of methods to a *Nature Communications* paper which features in the list of the top 50 biological papers published in the journal that year, as well as other *Nature Communications* and similar papers. We note that reviewers one and three both commented that the manuscript was "a good paper, worthy of publication" which was "robustly analysed using appropriate … methods."

Reviewer #3 (Remarks to the Author):

In this paper Shanmugam and colleagues generate new genome-wide and exome sequencing data from individuals who self-identify as Scottish travellers. This is a historically marginalized and highly understudied group that has far as I understand have never been characterized genomically prior to this work.

The authors first perform some analysis using the accompanied demographic data assembled during the DNA collection process of 125 individuals, and use this to examine the extent of enrichment of certain surnames within specific areas of Scotland. Then using a subset of samples (n=48) they generate genome-wide SNP data, and use this to examine the genetic structure and demographic history of the population. They do this using a suite of methods appropriate for genome-wide SNP data such as PCA, chromosome painting, IBD-based analysis, and ancestry clustering. They contexualize the Scottish traveller samples using reference data sets from "settled" British Isle's individuals and other traveller individuals.

They also parse the Scottish Traveller population by self-reported ethnicity, such as the number of grandparents being from a particular traveller population. The noteworthy take away from this analysis is that Scottish Traveller populations largely cluster with settled Scottish individuals, while exhibiting some internal population structure and endogamy as measured by ROH and IBD-based analysis.

They show no real relationship to Roma gypsy individuals from Europe. The Scottish traveller individuals with substantial self reported grandparental ancestry also form two clusters, with cluster A showing particularly small recent Ne and high levels of ROH.

The authors then generate exome sequencing data for all 125 samples to examine the extent that Scottish travellers might possess an enhanced risk for inherited disease due to enrichment of pathogenic SNP variants due to the increased rate of endogamy and drift. They find some evidence of this for a handful of variants related to diseases, though all in recessive form. They also make use of the fact that they also have a small number of Irish traveller genomes imbedded in their collection, and look for any overlap between variants common between them.

In general this paper is well written. In general I enjoyed reading it and found it interesting. The data generation is well decribed and robustly analyzed using the appropriate population genetic methods. I have no real issues from a technical level in terms of how the data have been analyszed.

We thank the reviewer for their positive comments.

By modern standards, generation of genome-wide SNP data for 50 individuals and exome data for 125 is not particulaly cutting-edge. However, it must also be taken into account that obtaining the samples themselves is highly challenging, and the authors appear to have gone through a very thoughful process to perform thus study, likely requiring significiant effort.

We thank the reviewer for complimenting the process we went through – it was indeed a significant effort, in part because of the long history of marginalisation and persecution. Nevertheless, as we have noted in response to reviewer two, we have sampled about 3% of the Traveller community in Scotland. The crux of the matter is whether the sample

sizes are sufficient to show whether the Scottish Travellers are distinct genetically, who they show affiliations with and whether they have undergone any founder effects on pathogenic variants, and the answer to all three questions is a clear yes.

While I would love to have seen high coverage whole genome data for these samples to bring it up to date with the current state-of-the-art and allow the author's to use more powerful ARG-based analyses for the popgen and explore things like polygenic non-exomic variants, I think the fact that this is the first Scottish traveller data of any kind makes the dataset valuable regardless.

We thank the reviewer for these comments – we agree that WGS would be very interesting, however it is outside the scope of the present study and funding envelope. We hope to be able to move on from this first description to WGS and the analyses they allow in the future.

The analysis of population genetic structure and demographic history is robust. I do not have many suggested improvements of this section. I think showing the close link to the settled Scottish population is important, given their marginalized status, and may even have important implications socially. From a techinical perspective, I would like to see a visualization of the IBD results. The analysis of its inference is described, but then it is just treated as raw material for methods like IBD-Ne and the clustering analysis. I would be interested in seeing for example what an IBD network looks like amongst the population and if it visualizes the two clusters somehow.

We thank the reviewer for the positive comments and agree it is interesting to inspect the IBD network. We have therefore now included a visualisation of the Scottish Traveller IBD network as Supplementary Figure 10. As can be seen below, tight clustering is observed for samples belonging to the fineSTRUCTURE cluster Scot-TravA, and a looser clustering for samples from Scot-TravB, with inter-cluster connections as well.

Supplementary Fig. 10 Network representation of identity-by-descent sharing among Scottish Traveller samples placed in the Scottish Traveller fineSTRUCTURE genetic clusters. *Each node (vertex) in the network graph represents one Scottish Traveller individual, with each connection (edge) representing the sum total of IBD segments from 1 - 120 cM in*

length that are shared between them. Network layout was achieved with the force-directed algorithm from Fruchterman-Reingold. Pairs of relatives closer than 2nd degree have been pruned. On the left is the layout when using every edge recorded. On the right is the layout when only the five edges with the greatest sum IBD length ("weight") for each node are used. Individuals are colour-coded by their fineSTRUCTURE cluster membership, and edge width is proportional to the sum IBD length shared between two Traveller individuals. The graph was created in R, using the igraph and ggplot2 R packages. Tight clustering (high IBD sharing) can be observed for members of the Scot-TravA cluster, with looser connections between members of Scot-TravB; and inter-cluster connections as well.

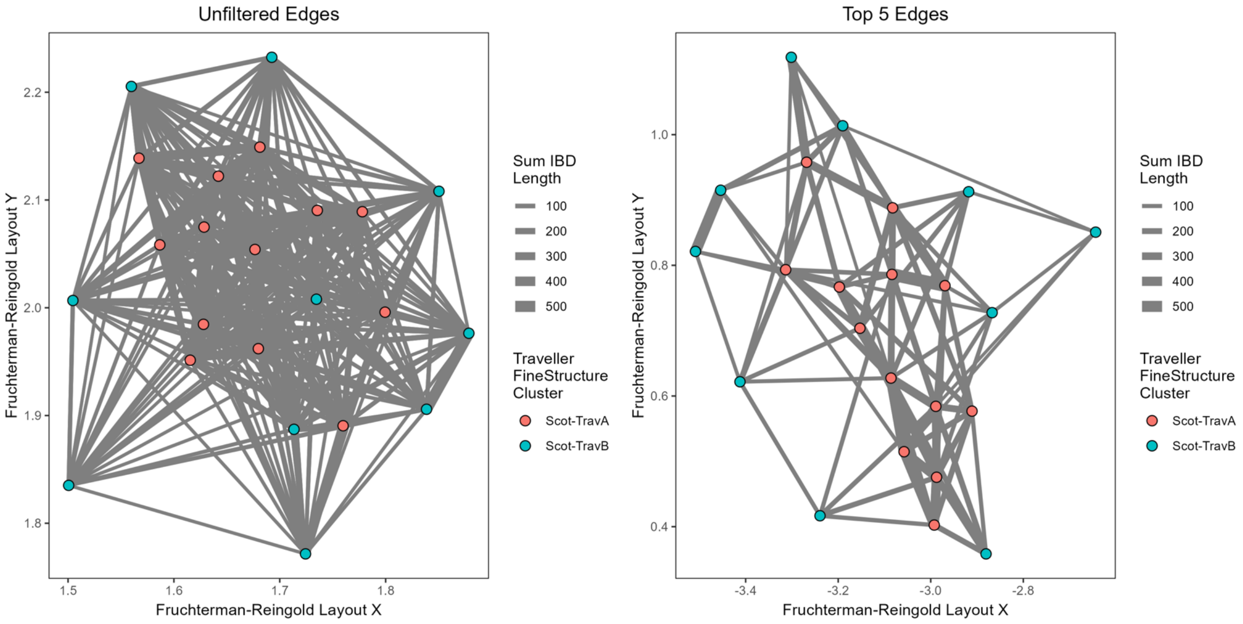

I found the pathogenic variant section less compelling. It finds some risk variants and is largely descriptive, but there is no real take away other than "this is important to know". No link is really made to what is found and what is known about about specific diseases/disorders Scottish traveller's tend to exhibit most. Do the findings appear to fit the clinical history of the population?

We agree with the reviewer that it would be very interesting to link our findings to what is known about specific diseases in the Scottish Travellers. However, we were unable to do

so, because there are no published papers about clinical history or diseases, Mendelian or otherwise, in the Scottish Travellers. The only literature consists of analyses of census data, which shows numerous health issues are more prevalent in the community (https://www.gov.scot/publications/gypsy-travellers-in-scotland-an-analysis-of-scotlands-census-2022/pages/health/). This lack of research makes our findings all the more important, as they highlight for the first time diseases that are likely to be found in the community, and which clinicians should be aware of. That is the take-away here. We now state (lines 743-746) that there is "an opportunity to engage with this population regarding a bespoke community-based reproductive carrier screening programme, similar to J-netics, a charity organisation in the UK whose purpose is to prevent and diagnose 47 predominantly Jewish recessive disorders, and raise awareness about them". We build further on this point, at lines 746-749: "In the meantime, clinicians who care for Scottish Travellers should be made aware of the predicted enrichment for the five most uplifted disorders in Table 2, and the scope for otherwise rare recessive Mendelian diseases generally to be more prevalent than in is usually the case in Scotland."
We have also added the following statement near the beginning of the Results section on clinical variants (lines 460-461), to help the reader understand the motivation: "To date, there is no scientific literature on the prevalence of Mendelian disorders among the Scottish Travellers."

Also, despite the high levels of homozygosity in the population, the pathogenic variants are usually found in singleton form, making the recessive variants largely harmless. Is this the result of the sample collection, for example only picking healthy individuals somehow? Or is there a broader evolutionary argument being made here about the purging of very deleterious dominant mutations? Given the large exome dataset, it would probably be worth examining how the allele frequency spectrum of the population looks at synonymous vs nonsynomymoys sites, and comparing the distribution of fitness effects to the "settled" Scottish individuals? Is there any evidence of a skew? This would be much more useful I think than the large section on mtDNA haplotypes, which could be shortened as it does not add much.

The recruitment didn't favour healthy people, but it is likely that cases of most of these Mendelian disorders would suffer ill health which would decrease their chances of volunteering for the study. While we didn't observe any homozygotes, the increased

frequency of the drifted variants means that without doubt there will be an increased disease burden in the population for these variants, some of which have allele frequencies of 3-4%, meaning up to ~0.1% of the population will be homozygous, over a million-fold more than in non-Finnish Europeans in gnomAD. Taking figures from Supplementary Table 9 for *CRB2* p.Gly1036AlafsTer43 as a worked example: MAF in Scottish Travellers is 0.022 versus that in NFE gnomAD of 0.000009; taking the squares to estimate the frequency of homozygotes (affecteds), gives 0.00048 vs. 7.7 x 10^{-11}, a ratio of over 6 million.

We are not making the case for evolutionary purging here and we don't believe we would have power to detect differences in synonymous: non-synonymous ratios.

Also beyond the "this is important to know", does the observed distribution of pathogenic variants mean some specific health policy intervention should be explored. There is a brief mention of the similarity to Jewish recessive screening, so a bit more detail here would be useful.

As mentioned above, we have now further clarified that the most appropriate health policy intervention would be a reproductive carrier screening programme (line 741) and added further statements regarding raising clinician awareness (lines 746-748).

Specific comments
Line 151: "All Traveller Genes participants with DNA passing QC were exome sequenced, and 48 unrelated participants with maximal Traveller ancestry had additional genome-wide SNP array genotypes generated, for joint analysis with reference populations."
Does "maximal Traveller ancestry" mean four grand parents or is it determined by genetic ancestry?

We have now clarified the meaning – we selected unrelated samples with the maximum number of grandparents available to represent each of the different ancestries. The revised text (lines 155-156) now reads: "…48 unrelated participants representing the different ancestries with four Traveller grandparents (n=30), three Traveller grandparents (n=13) or two Traveller grandparents (n=5) had additional…"

Line 551: "This is the first genetic study of the Scottish Travellers and should therefore help to clarify the debate around their origins"
I am not really sure the study tells us about the origins of Scottish Travellers. It tells us about the genetic structure, which is interesting, but is very different from "origins". At most the author's have ruled out a Roma connection. But the analysis has not revealed things like the time or place of their origins (which is probably multifactorial). I think the discussion should reflect that

We have now replaced the word “origins” with “ancestral ties”. We show that these link to Scotland, not to the Roma. We agree that the time(s) and place(s) of Traveller origins are complex. Future studies with larger samples and whole genome data may be able to shed light on these questions, if the community wished. We do note though that the mitochondrial analysis is relevant here. Although the mtDNA data do not identify any links between Scottish Travellers and the Roma (which is only detectable for the English Gypsies), it does specifically identify links with non-local, mostly Southern Mediterranean populations, such as Italy, Spain and France. The fact that these are not replicated among the local settled community (including 670 mitogenomes from the British Isles) suggests that these links might represent population ties that are unique to the Scottish Travellers.

Response to second round of review

Reviewer #1 (Remarks to the Author):

I think this paper is good and contains some important work. The data is important to publish. With regard to the audience, I still think it might be better to split into two papers, one sociology of the Scottish Travellers and then have a more concise condensed one discussing the results of the array and sequencing?

However, I understand where the authors are coming from. If the journal can afford the space the sociological element takes up then it would be fine but if space is tight I think the authors could still consider getting two papers out of this?

We thank the reviewer for the positive comments. We have been advised by the editor that there is no need to split the manuscript.

Reviewer #2 (Remarks to the Author):

The authors have made a remarkable effort to refine the manuscript; the updated presentation of the hypotheses and results represents a substantial improvement.

However, I would appreciate further clarification regarding the proposed substructure of Scottish Travellers (Groups A and B), as one of the conclusions appears slightly paradoxical. The manuscript suggests that Group B is closer to the ancestral population, while Group A is a drifted descendant. Yet, the authors also correctly note that Group B is an admixed group that has experienced recent gene flow. Given this admixture, is it possible that Group A actually represents the more 'ancestral' lineage, one that appears more distant simply because it has remained isolated and undergone significant genetic drift, rather than having its distance “reduced” by gene flow as seen in Group B? I would welcome the authors' comments on this alternative interpretation.

We thank the reviewer for the compliments and also for raising this alternative hypothesis.

Our explanation was based on the simplest scenario fitting with the observations regarding drift and admixture, and that group A could be modelled as 93% from group B, while B only derived 54% from Group A.

While it is true that Group A are more drifted and Group B are more admixed, it is difficult to disentangle which group is closer to the ancestral population. We have therefore now modified the text to show this, by adding “potentially” at line 301 and then in the discussion, at lines 617-619, adding, “Another possibility is that Scot-TravA is more similar to the original population, retaining a more distinctive signature through isolation and stronger drift, while Scot-TravB received more gene flow.”

We have also deleted lines 261-263, which are duplicative with later text and were included in error.

Reviewer #3 (Remarks to the Author):

In general the author's have addressed my comments. However, their reply to my comment regarding the allele frequency spectrum is not really sufficient:

"we don’t believe we would have power to detect differences in synonymous: non-synonymous ratios." What is this argument based on?

The authors are claiming that rare deleterious alleles are enriched in the traveller population because of their lower Ne. They are essentially making an argument for higher genetic load when comparing to the non-Finnish Europeans in gnomAD. The relative AFS for non-synonymous versus synonymous sites compared to a control population from 125 individuals with whole exome data is likely to result in some skew in the rare variant category, for example simply comparing to 1000G Europeans. In addition, the authors could also look at the distribution of pathogenic variants within and outside ROH. I would at least like to see these analyses attempted before simply dismissing them, as positive results would very much strengthen the case of increased attention on disease burden.

While there are 125 whole exome sequences, only 33 of these are for participants declaring all four grandparents to be Scottish Travellers (Table 1). As there is no easy general method to infer the Traveller or non-Traveller origins of *all* exonic variants observed in the half-Travellers, the Travellers with four Traveller grandparents are the only subset which could be used for the proposed analyses. There would thus be very little power to detect differences in population genetic quantities such as the synonymous: non-synonymous ratio or allele frequency spectrum, particularly for rarer variants, when compared to non-Finnish Europeans from the 1000 Genomes Project. For instance, many variants present at 1% allele frequency will not have been observed in the sample.

We are not claiming that rare deleterious alleles are enriched in the Traveller population. We rather have shown that at least five *individual* variants which are considered pathogenic in ClinVar are more common in the Traveller population than elsewhere and that clinicians treating this community should be made aware of this. Moreover, many individuals are carriers of multiple otherwise rare pathogenic variants, which may be important for genetic counselling in the community.

To make this clearer, we have now replaced the one instance of “deleterious” with “pathogenic” (line 949).

We feel that the correspondence between *pathogenic* (as detailed in ClinVar for individual variants with clinical evidence) and *deleterious* (predicted bioinformatically) is a large subject, beyond the scope of this manuscript. Furthermore, such a broad approach was not discussed or approved by the Traveller Genes PPI committee when setting up the project and so would go beyond the co-production all have agreed. With marginalised minority groups such as the Scottish Travellers, we have a duty to avoid potentially stigma-inducing investigations, such as those involving *genetic load*, without clear benefits and in-depth consultation with the community. In order to further avoid straying into this territory, we have removed one instance of “burden” used in a meaning similar to this.
